# Detection and evaluation of clusters within sequential data

**DOI:** 10.1007/s10618-025-01140-4

**Published:** 2025-08-14

**Authors:** Alexander Van Werde, Albert Senen–Cerda, Gianluca Kosmella, Jaron Sanders

**Affiliations:** 1 Department of Mathematics & Computer Science, TU/e, Eindhoven, The Netherlands; 2 LAAS–CNRS, IRIT–CNRS, and Université de Toulouse, Toulouse, France; 3 Department of Electrical Engineering, TU/e, Eindhoven, The Netherlands

## Abstract

Sequential data is ubiquitous—it is routinely gathered to gain insights into complex processes such as behavioral, biological, or physical processes. Challengingly, such data not only has dependencies within the observed sequences, but the observations are also often high-dimensional, sparse, and noisy. These are all difficulties that obscure the inner workings of the complex process under study. One solution is to calculate a low-dimensional representation that describes (characteristics of) the complex process. This representation can then serve as a proxy to gain insight into the original process. However, uncovering such low-dimensional representation within sequential data is nontrivial due to the dependencies, and an algorithm specifically made for sequences is needed to guarantee estimator consistency. Fortunately, recent theoretical advancements on Block Markov Chains have resulted in new clustering algorithms that can provably do just this in synthetic sequential data. This paper presents a first field study of these new algorithms in real-world sequential data; a wide empirical study of clustering within a range of data sequences. We investigate broadly whether, when given sparse high-dimensional sequential data of real-life complex processes, useful low-dimensional representations can in fact be extracted using these algorithms. Concretely, we examine data sequences containing GPS coordinates describing animal movement, strands of human DNA, texts from English writing, and daily yields in a financial market. The low-dimensional representations we uncover are shown to not only successfully encode the sequential structure of the data, but also to enable gaining new insights into the underlying complex processes.

**Supplementary Information:**

The online version contains supplementary material available at 10.1007/s10618-025-01140-4.

## Introduction

Modern data often consists of observations that were obtained from some complex process, and that became available sequentially. The specific order in which the observations occurred then often matters: future observations frequently correlate with past observations. By identifying a relation between subsequent observations within the sequential data one may hope to gain insight into the underlying complex process. The high-dimensional nature of modern data however can make understanding the sequential structure difficult. For example, on high-dimensional data, many algorithms slow down to an infeasible degree, overfitting may occur, and human interpretation becomes problematic.

In view of the challenges associated with the high dimensionality of processes and/or data, it is desirable to identify a latent structure which respects the sequential structure but has reduced dimensions. We therefore now focus on a popular class of methods for discovering latent structure in datasets: *clustering algorithms*. Clustering algorithms work by clustering together data points from a dataset that are “similar” in some sense. Let us illustrate by considering clustering in *nonsequential* data (i.e., data in which the order of the observations does not matter). If such data has a geometric structure for which a notion of distance is applicable, then one may call two points similar if their distance is small. This distance-based notion of similarity can then be leveraged with the well-known *K*-means algorithm for clustering point clouds (MacQueen 1967). Or, if the data instead has a graph structure, then it is natural to call two vertices of the graph similar if they connect to other vertices in similar ways. This second connection-based notion is then made rigorous in e.g. the Stochastic Block Model (Holland et al. 1983).

A natural notion of similarity between *sequential* observations—when the exact order of observations really does matter—may similarly be given. Consider the following informal criterion: “two observations are similar if and only if they follow after earlier observations in similar ways.” A recent model which makes this transition-based notion formal are Block Markov Chains (BMCs) (Sanders et al. 2020). Specifically, the BMC model assumes that the observations are the states of a Markov Chain (MC) in which the state space can be partitioned in such a manner that the transition rate between two states only depends on the parts of the partition in which these two states lie. Each part of the partition is also referred to as a *cluster*.

To give an example, consider the sequence of songs which a user of a music platform listens to. If they start with a song from the “Metal" genre, then the next song is likely to be from the same genre. Once they decide to switch genres, however, the user may be more likely to select the “Rock" genre than the “Disco" genre. The BMC model captures such information by allowing the transition probabilities to depend on the clusters—the music genres here—but not to depend on states within a cluster —the songs of a genre—so that the sequential dependence is entirely captured by the clusters. Actionable insight based on user data may then be derived from the BMC model, for example, by attaching user-specific clusters to recommendation systems, by using the clusters to determine the favorite genre of the user or by categorizing new songs given a small amount of user data. In a more general application area, algorithms for training agents with reinforcement learning have also recently appeared that use data to cluster the state space to improve the training sample efficiency (Zhu et al. 2021); see also Sect. 1.3.

The problem of clustering the observations in a single (possibly short) sequence of observations of a BMC was recently investigated theoretically (Zhang and Wang 2019; Sanders et al. 2020). For example, given a sample path generated by a BMC, an information-theoretic threshold below which exact clustering is impossible because insufficient data is available has been established in (Sanders et al. 2020, Theorem 1). Further, in (Sanders et al. 2020, Theorem 3) a clustering algorithm for BMCs was provided and shown to recover the underlying clusters whenever the implied conditions for recoverability are satisfied; so even when the sequence is short relative to the size of the state space. The fact that this algorithm is explicitly designed to manage in sparse regimes where the amount of data is small is a favorable property for applications where gathering large volumes of data may be expensive and laborious. Until now, however, a broad study on the performance of this clustering algorithm when applied to sequential data obtained from actual real-life processes was not provided. The purpose of the current paper is to address this important gap in the literature.

Let us remark that our goal is not to compare the performance of the BMC clustering algorithm relative to other algorithms. Indeed, the BMC algorithm is explicitly designed to manage in sparse regimes where the amount of data is small. Most model-free algorithms on the other hand, such as those based on deep learning, excel when one has access to large amounts of training data. The outcome of a direct comparison would consequently be predetermined by the choice of the amount of training data. Our goal is rather to study this new clustering algorithm’s capabilities to provide meaningful insights into real-life complex processes, and to supplement the theoretical understanding of the BMC-based algorithm with a practical viewpoint. To achieve this goal, we focus on questions such as:

1. How can the BMC model practically aid in data exploration of sequential data obtained from real-life data?
2. How can one statistically decide whether the BMC model is an appropriate model for the sequence of observations? How can it be detected that either a simpler model than a BMC would suffice, or a richer model is required?
3. Can the algorithm be expected to give meaningful results despite the sparsity and complexity of real-life data? Is the clustering algorithm robust to model violations?

### Contributions

We investigate the performance of the BMC-based algorithm using a diverse collection of datasets that come from the fields of ethology, microbiology, natural language processing, and finance. Specifically, we investigate sequences of:

- Global Positioning System (GPS) coordinates from animal movements.
- Codons in human Deoxyribonucleic Acid (DNA).
- Words in Wikipedia articles.
- Companies in the Standard and Poor’s 500 (S&P500) with the highest daily returns.

To each dataset we apply the BMC-based clustering algorithm to uncover underlying clusters. Our findings are summarized in Sect. 1.2 and confirm that the algorithm can uncover relevant latent structure in practice.

Evaluating the performance of a clustering algorithm and the appropriateness of the model in a real-life scenario can be nontrivial. For instance, unlike scenarios with synthetic data, one can not compare with a ground-truth cluster structure. To answer the second and third research questions raised above, Sect. 3.3 hence explores a set of experimental tools that incorporating insights from statistics (Bozdogan 1987; Kullback and Leibler 1951), machine learning (Lewis et al. 2004), and random matrix theory (Sanders and Senen-Cerda 2023; Sanders and Van Werde 2023). These tools are applied to the aforementioned real-life datasets in Sect. 5.1 and give us insights on the suitability of the model (both positive and negative, depending on the dataset).

Finally, we programmed a Dynamic-link library (DLL) in C++ that allows efficient simulation and analysis of trajectories of a BMC. Our source code can be found at https://gitlab.tue.nl/acss/public/detection-and-evaluation-of-clusters-within-sequential-data. We distributed this DLL with an easy-to-use Python module called *BMCToolkit* at https://pypi.org/project/BMCToolkit/. This approach of interfacing with a DLL written in C++, and careful parallelization and compilation, outperformed earlier versions of the module written entirely in Python considerably. This enabled us to tackle larger sequences with more distinct observations.

So, to summarize, we evaluated the BMC-based algorithm across diverse real-life datasets and demonstrate its practical applicability, filling a gap in the literature. Moreover, along the way, we developed experimental evaluation tools and efficient implementations that are expected to be crucial for future practical applications.

### Summary of the detected clusters

**Fig. 1:**
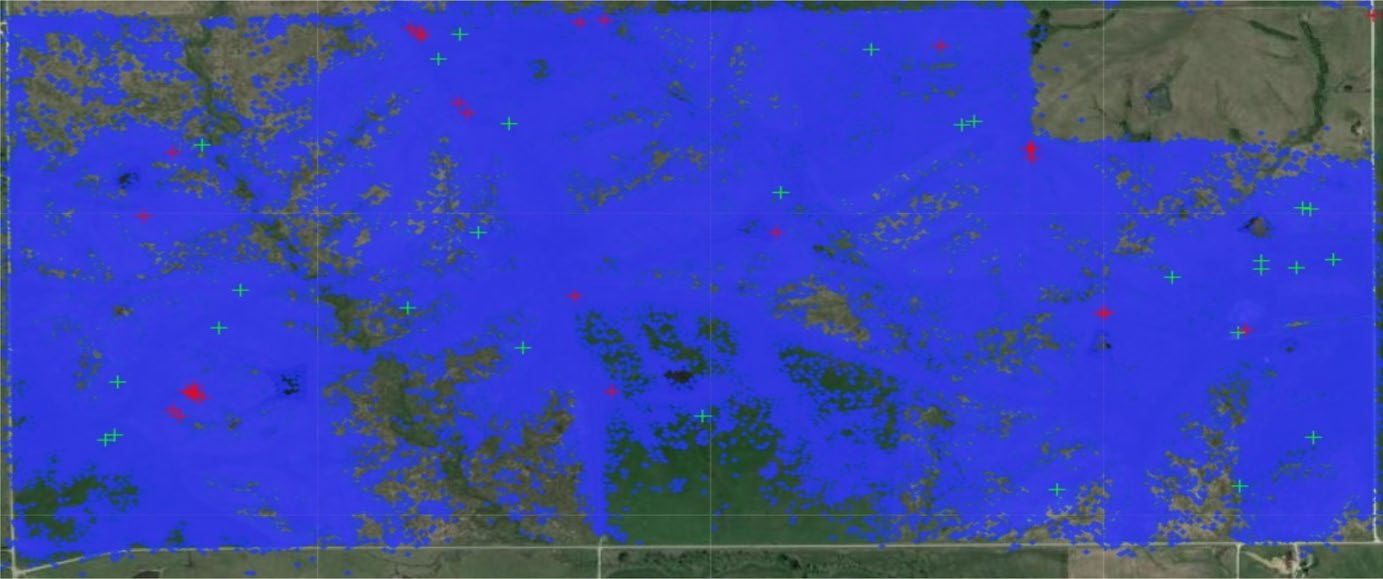
The raw GPS data from the “Dunn Ranch Bison Tracking Project” (see Stephen Blake 2017, #8019591) projected onto a satellite image. Each blue point depicts a single recorded datapoint. Note that it is not easy to extract insight from this scatter plot, and one should really aggregate the data in some useful manner. The clustering techniques that we implement do this by taking sequential information into account, resulting in the much more insightful Fig. 2 below

**Fig. 2:**
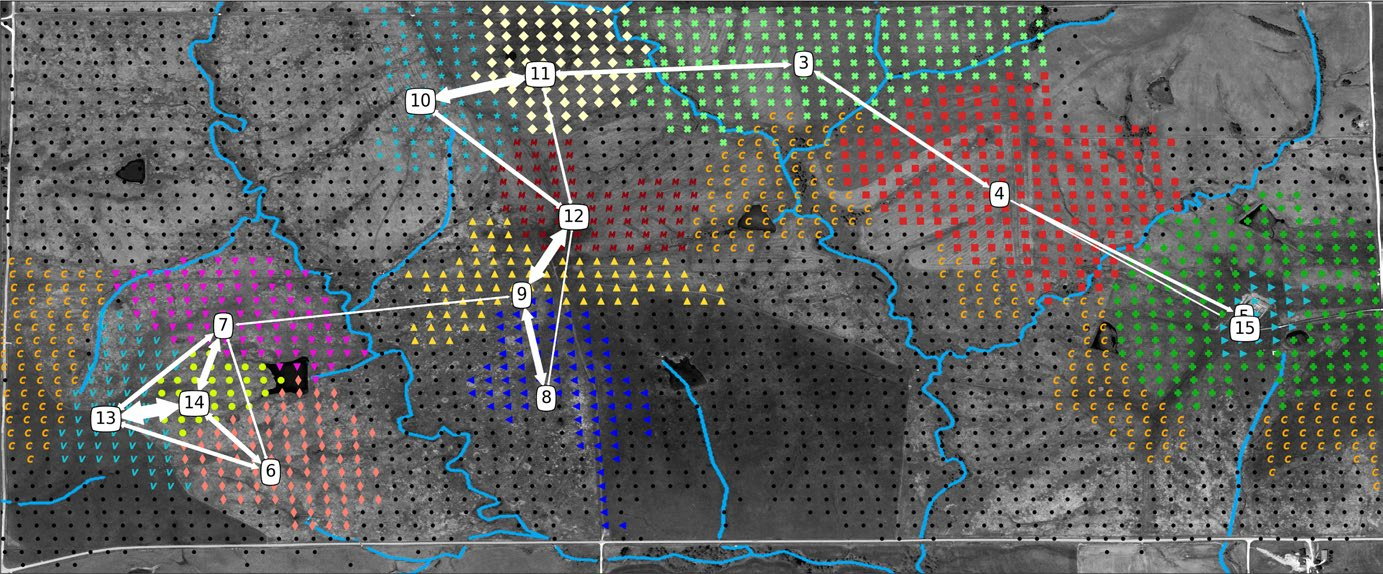
In the background: a satellite image of Dunn Ranch with rivers highlighted in blue for visualization purposes. In the foreground: the detected clusters as colored bullets, cluster centers indicated by boxes containing the cluster number, and edges between the boxes indicating the transitions between clusters with probability of at least $1\%$. Thicker arrows correspond to higher transition probabilities. Self-transitions and the clusters 1 and 2 are omitted, because they are noninformative

Our findings in the animal movement data are particularly striking. There, a scatter plot of the data yields a picture which is difficult to interpret (Fig. 1). After clustering, a picture can be displayed which provides significantly more insight (Fig. 2).

Specifically, the graph displayed by the white arrows in Fig. 2 gives insight into the global topological structure of the latent dynamics of the animal movements. Comparing to a satellite image of the area reveals that the boundaries between clusters often correspond to barriers, here rivers, which hinder animal movements. We emphasize that the algorithm does not access the satellite image: the aforementioned features are found using solely the sequential structure of the data. In other datasets, it could therefore also be possible to detect structures of different varieties such as breeding sites, human presence, territorial boundaries, roads, or pesticide-caused chemical barriers which may be relevant for animal behavioral studies (Bélisle 2005; Urban and Keitt 2001; Vuilleumier and Metzger 2006; Keeley et al. 2021) or wild-life conservation Robert McDonald and Cassady (St. Clair 2004; Taylor and Goldingay 2010; Ruby et al. 1994). Let us finally note that this paper’s model evaluation tools are found to be informative for this dataset, suggesting room for future methodological expansion.^1^

In DNA, the algorithm leads us to rediscover phenomena that are known in the genomics community as *codon–pair bias* and *dinucleotide bias* (Gutman and Hatfield 1989; Coleman et al. 2008; Kunec and Osterrieder 2016). More precisely, in Table 2 it may be observed that cluster $k = 2$ mainly contains codons ending with the nucleotide *C* whereas cluster $k = 3$ mainly contains codons starting with nucleotide *G*. Closer inspection of the transition rates between these clusters reveals that we only rarely observe transitions from cluster $k = 2$ to cluster $k = 3$: see Fig. 6a. In other words, there is a bias against a *C*–to–*G* transition on the junction between two codons. It is further interesting to note that our model evaluation tools suggest that, while not perfect, the BMC assumption seems reasonable for this dataset; see Sect. 5.1.2.

In the text data we consider a document classification task and find that a BMC-based cluster improvement algorithm performs better than plain spectral clustering; see Table 3 for the results and Sect. 3.2 for the algorithms. Recall that high performance here is not our main objective. Rather, it serves as an evaluation tool (see Sect. 3.3). If one simply desires optimal performance, not an interpretable model, then neural machine learning methods (Minaee et al. 2021) will outperform BMC-based methods on this task because large volumes of data are available in natural language processing. Our point is that because the improvement algorithm exploits the model assumptions more aggressively than the spectral algorithm, the findings suggest that the model itself brings merit. In Sect. 5.1.3, we again find that the evaluation tools are informative, uncovering model violations whose nature we can clarify.

Finally, the S&P500 dataset is distinct as it gives the least clear conclusions. The difficulty of this dataset is due to the combination of sparsity and a nuisance factor. We discuss this dataset extensively in Sects. 5.1.4 and 5.2 as an illustrative dataset for our evaluation tools in a difficult setting. To summarize: we find that a simpler model called a 0th-order BMC (see Sect. 3.1) can describe its statistical aspects, while simultaneously that there are indications that a 1st-order BMC is also suitable.

### Related literature

***Clustering in MCs and random graphs***

Algorithms for detection in BMCs have been studied in (Zhang and Wang 2019; Sanders et al. 2020) including information-theoretic limits stating when it is impossible to recover clusters in (Sanders et al. 2020), and estimation of the number of clusters was recently studied in (van Vuren et al. 2024). Other clustering algorithms and models that use spectral decompositions to uncover clusters or low-rank structures based on trajectories of MCs are studied in (Duan et al. 2019; Bi et al. 2022; Du et al. 2019; Zhu et al. 2021).

The clustering algorithm involves a spectral step that relies on random matrices constructed from sample paths of BMCs. This motivated further theoretical studies of random matrices constructed from Markovian data in (Sanders and Senen-Cerda 2023; Sanders and Van Werde 2023; Van Werde and Sanders 2023). In (Sanders and Van Werde 2023), convergence of singular value distributions in the BMC model is established in the dense regime $\ell = \Theta (n^2)$. We use and refine this result in our experiments.

Community detection in random graphs, such as those produced by the Stochastic Block Model, is a closely related area of research. The distinction with clustering in BMCs is that the vertices within a single observation of a random graph are clustered, instead of the observations within sequential data. We refer the reader to (Gao et al. 2017) for an extensive overview on cluster recovery within the context of the Stochastic Block Model, and to (Fortunato 2010) for an overview on community detection in graphs.

***Different types of clustering for sequential data***

In the reviews (Zolhavarieh et al. 2014; Aghabozorgi et al. 2015), some further lines of research that relate to both clustering and sequential data are divided into three categories. First, *whole-time-series clustering* groups the trajectories of different time-series (Aghabozorgi et al. 2015; Liao 2005; Driemel et al. 2016). Second, *clustering of subsequences of a time-series* where individual time-series are extracted via a sliding window (Lin et al. 2003; Rakthanmanon et al. 2011; Rodpongpun et al. 2012). Finally, there is *time-point clustering* which includes problems like segmenting an *n*-element sequence into *k* segments, that can come from *h* different sources; see e.g. (Gionis and Mannila 2003; Mörchen et al. 2005). These three categories are all distinct from the notion which we employ, but the final category is closest.

***State space reduction in decision theoretical problems***

Studying clustering in MCs is also motivated by the necessity for effective state space reduction techniques in decision theoretical problems. For example, in Reinforcement Learning, Markov Decision Processes, and Multi-Armed Bandit problems it is known that learning a latent space reduces regret in Multi-Armed Bandit problems (Maillard and Mannor 2014; Azar et al. 2013). State aggregation and low-rank approximation methods have been studied for Markov Decision Processes as well as Reinforcement Learning, see (Li et al. 2006) and (Ong 2015; Azizzadenesheli et al. 2016; Yang et al. 2019), respectively. The idea to cluster states in Reinforcement Learning based on the process’ trajectory was first explored in (Singh et al. 1994; Ortner 2013).

***Some related experiments in microbiology, natural language processing, ethology, and finance***

Using similar means as in the animal movement data in this paper, GPS coordinate sequences for New York City taxi trips are investigated in (Zhang and Wang 2019; Bi et al. 2022; Sanders and Van Werde 2023). The found low-dimensional representation of the taxi data also gives insight into taxi customer behavior, just as it does in this paper for the animal movement behavior. The taxi data is however quite different from the animal movement data: taxi transitions tend to be between far away entrance and drop-off locations.

MC models for the sequence of nucleotides or codons in DNA are considered in (Almagor 1983; Jorre and Curnow 1976; Robin et al. 2005). The current paper is the first time that a BMC was used for this task. MCs and hidden Markov models are often used in natural language processing; see (Manning and Schutze 1999). In (Gialampoukidis et al. 2014) the transition between the Dow Jones closing prices are described as a MC close to equilibrium. Other references for MC models in finance include (Zhang and Zhang 2009; van der Hoek and Elliott 2012; Mamon and Elliott 2007).

### Structure of this paper

We introduce the problem of clustering in sequential data in Sect. 2. We describe the BMC as well as other models that appear in our experiments in Sect. 3.1, and briefly discuss the advantages of a model-based approach. Next, we introduce the clustering algorithm in Sect. 3.2. We describe there also our C++ implementation of this clustering algorithm, which we have made publicly available as a Python library. Sect. 3.3 describes practical tools to evaluate clusters found in datasets in the absence of knowledge on the underlying ground truth. Sect. 4.1 introduces the datasets and explains our preprocessing procedures; Sects. 5.1, 5.2 then extensively evaluate the clusters detected within these datasets. Finally, Sect. 6 concludes with a brief summary of our findings.

## Problem formulation

We suppose that we have obtained an ordered sequence of $\ell \in \mathbb {N}_{+}$ discrete observations

1 $$\begin{aligned} X_{1:\ell }:= X_1 \rightarrow X_2 \rightarrow \cdots \rightarrow X_\ell \end{aligned}$$

from some complex process. The observations can be real numbers or abstract system states; as long as the observations come from a finite set. We assume specifically that there exists a number $n \in \mathbb {N}_{+}$ such that $X_t \in [n]:= \{ 1, \ldots , n \}$ for all $t \in [\ell ]$. Here, *n* can be interpreted as the number of distinct, discrete observations that are possible.

Given such ordered sequence of observations, we wonder whether there exists a map $\sigma _n: [n] \rightarrow [K]$ with $1 \le K\le n$ an integer, such that the ordered sequence

2 $$\begin{aligned} \sigma _n(X_{1:\ell }):= \sigma _n(X_1) \rightarrow \sigma _n(X_2) \rightarrow \cdots \rightarrow \sigma _n(X_\ell ) \end{aligned}$$

captures dynamics of the underlying complex process. Observe that $\sigma _n$ defines *clusters*:

3 $$\begin{aligned} \mathcal {V}_k:= \bigl \{ i \in [n] \mid \sigma _n(i) = k \bigr \} \end{aligned}$$

for $k \in [K]$. Furthermore, $\mathcal {V}_k \cap \mathcal {V}_l = \emptyset$ whenever $k \ne l$ and $\cup _{k=1}^K\mathcal {V}_k = [n]$.

The clusters $\mathcal {V}_1, \ldots , \mathcal {V}_K$ are particularly interesting when $K\ll n$. In such a case the *clustered* process $\{ \sigma _n(X_t) \}_{ t }$ lives in a much smaller observation space than the original process $\{ X_t \}_{ t }$. The reduction may then prove to be beneficial for computational tasks since the time complexity of some algorithms depends on the size of the observation space. If (2) furthermore indeed captures the dynamics of the complex process, then it is not unreasonable to expect that the clusters $\mathcal {V}_k$ could themselves be meaningful thus allowing for human interpretation of the data.

## Preliminaries

### Models

#### Main model: BMC

**Fig. 3:**
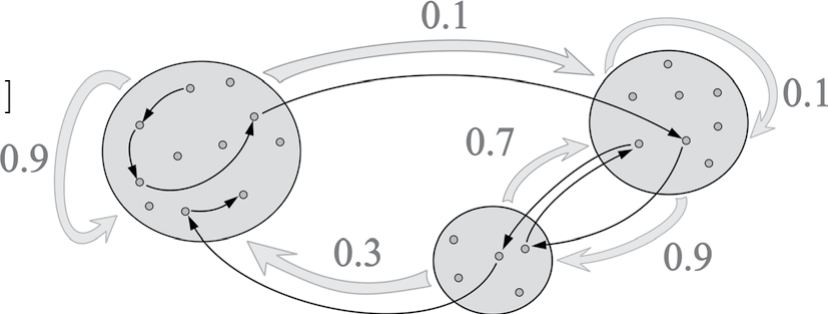
A visualization of a BMC with $K=3$ clusters and $p = [[0.9,0.1,0],[0,0.1,0.9],[0.3,0.7,0]]$. The thick arrows visualize to the cluster transition probabilities $p_{k,l}$, while the thin arrows visualize the transitions of a sample path $\{ X_t \}_{ t }$. Figure courtesy of (Sanders and Van Werde 2023)

Formally, a *1st-order BMC* is a discrete-time stochastic process $\{ X_t \}_{ t \ge 0 }$ on a state space $\mathcal {V}:= [n]$ that satisfies not only the MC property

$$\begin{aligned} \mathbb {P} [ X_{t+1} = j \mid X_{t} = i, \ldots , X_0 = i_0 ] = \mathbb {P} [ X_{t+1} = j \mid X_t = i ] \ \forall j,i,i_{t-1},\ldots ,i_0 \in [n]; \end{aligned}$$

but also that there exists a cluster assignment map $\sigma _n: [n] \rightarrow [K]$ and a stochastic matrix $p\in \mathbb {R}^{K\times K}$ with

4 $$\begin{aligned} P_{i,j}:= \mathbb {P} [ X_{t+1} = j \mid X_t = i ] = \frac{p_{\sigma _n(i), \sigma _n(j)}}{ \# \mathcal {V}_{\sigma _n(j)} } \end{aligned}$$

with $\mathcal {V}_k$ defined as in (3). Fig. 3 depicts a BMC on $K= 3$ clusters.

The BMC model can be viewed as an ideal case for the setup of (2). The reduced process $\{ \sigma _n(X_t) \}_{ t }$ not only captures *some* part of the dynamics of the true process but rather *all* the order-dependent dynamics. Indeed, for any $t>1$ it holds that conditional on $\sigma _n(X_t) = k$ the observation $X_t$ is chosen uniformly at random in the cluster $\mathcal {V}_k$. The previous state $X_{t-1}$ hence influences the next cluster $\sigma _n(X_t)$ but does not provide any further information about the precise element in $\mathcal {V}_{\sigma _n(X_t)}$.

If *p* defines an ergodic MC, then the BMC has a unique *state equilibrium distribution* $\Pi \in [0,1]^n$. This distribution has the symmetry property that $\Pi _j$ only depends on the cluster assignment $\sigma _n(j)$:

5 $$\begin{aligned} \Pi _j&:= \lim _{t \rightarrow \infty } \mathbb {P} [ X_t = j \mid X_0 = i_0 ] \nonumber \\&= \frac{1}{\# \mathcal {V}_{\sigma _n(j)}} \lim _{t\rightarrow \infty } \mathbb {P} [ \sigma _n(X_t) = \sigma _n(j) \mid \sigma _n(X_0) = \sigma _n(i_0) ] =: \frac{ \pi _{\sigma _n(j)} }{ \# \mathcal {V}_{\sigma _n(j)} }. \end{aligned}$$

Here, $\pi \in [0,1]^K$ is the equilibrium distribution of the MC with transition matrix *p*.

#### Other models for experimentation

Recall that one of our goals is to develop tools for evaluating whether the BMC model is appropriate. In this setting it is often useful to compare with alternative models. The models that we have used are collected here for easy reference.

0***th-order BMCs***

Let $K\in [n]$ and consider an arbitrary probability distribution $\eta : [K] \rightarrow [0,1]$. A *0th-order BMC* is then a BMC with cluster transition matrix $p_{k,l}:= \eta _l$ for all $k,l \in [K]$. The 0th-order BMC will serve as a benchmark to assert whether the structures we find are due to the sequential nature of the process and do not admit a simpler explanation.

Namely, observe that in a 0th-order BMC each next sample $X_{t+1}$ is independent of the previous sample $X_t$. A 0th-order BMC therefore generates sequences of independent and identically distributed random variables. This is contrary to a 1st-order BMC, which generates a sequence of dependent random variables. The probability of a specific observation does depend on the cluster of the observation, and specifically is identical for every observation within that cluster.

*r* ***th-order*** ***MCs***

Conversely, it could occur that sequential dependencies are not limited to the single previous observation. We hence also consider models with higher-order dependencies.

Consider a discrete-time stochastic process $\{ Y_t \}_{t=1}^\ell$ (not necessarily a MC) that satisfies $Y_t \in [n]$ for some $n \in \mathbb {N}_{+}$. We say that $\{ Y_t \}_{t \ge 1}$ is an *rth-order MC* if and only if for all $t \in [\ell - r]$, all $i^r = (i_1, \ldots , i_r) \in [n]^r$ and $j \in [n]$,

6 $$\begin{aligned} \mathbb {P} [ Y_{t+1} = j&\mid Y_t = i_r , Y_{t-1} = i_{r-1} , \ldots , Y_{t-r+1} = i_1, Y_{t-r} = s_{t-r}, , \ldots , Y_{1} = s_{1} ] \nonumber \\ = \mathbb {P} [ Y_{t+1} =&j \mid Y_t = i_r , Y_{t-1} = i_{r-1} , \ldots , Y_{t-r+1} = i_1 ] =: P^r_{i^r,j} \end{aligned}$$

for some transition matrix $P^r \in [0,1]^{n^r \times n}$. By imposing that the entry $P^r_{i^r,j}$ may only depend on the cluster assignments $\sigma _n(j),\sigma _n(i_1),\ldots ,\sigma _n(i_r)$ one gets a model with longer dependencies which still has a ground-truth notion of clusters, called an *rth order BMC*.

Given such cluster assignments, Sect. 3.3 provides methods to evaluate what order is the best fit for provided sequential data. So, in practice, these methods do require the identification of such cluster assignments first. If one would simply apply the clustering algorithm for 1st-order BMCs to a BMC of much higher order (a task for which the algorithm was not explicitly designed), then one must be aware of a few limitations. Specifically, if *n* is large and $r > 1$, then the spectral step can become computationally infeasible in practice as the empirical frequency matrix has size $n^r \times n$. Further, even after clustering, the number of parameter grows exponentially with *r*, so choosing a model with large time dependence risks overfitting the data if its amount does not scale accordingly. Nonetheless, if one is mainly concerned with goodness–of–fit and not necessarily with interpretability, then a moderately higher order *r* can be suitable: see Sect. 5.2 for our findings with real-world data.

***Perturbed BMCs*** Finally, we consider an alternative model which concerns the scenario where a BMC captures the dynamics only partially. Specifically, a *perturbed BMC* mixes a 1st-order BMC on [*n*] that has transition matrix $P_{\text {BMC}}$ with a generic 1st-order MC on [*n*] that has transition matrix $\Delta$ by consideration of the MC with transition matrix

7 $$\begin{aligned} P_{\text {Perturbed}}:= (1-\varepsilon )P_{\text {BMC}} + \varepsilon \Delta . \end{aligned}$$

The parameter $\varepsilon \in [0,1]$ measures how much the dynamics are affected by the non-BMC part $\Delta$. Whenever we use a perturbed BMC, we specify $\Delta$ on the spot.

#### Concerning model misspecification

In practice, it is unlikely that the complex process $\{ X_t \}_t$ is exactly a BMC. One may hence wonder about the dangers of model misspecification:

1. Is the clustering algorithm robust to violations of the model assumption?
2. When concerned with a downstream task, does the BMC model provide any benefit when compared to models with fewer assumptions?

In this regard we would like to point out that the data which we consider is not only complex but oftentimes also sparse. Let us illustrate the principle by a numerical experiment on synthetically generated datasets.

To model a violation of the model assumptions while retaining a sensible notion of ground-truth communities we considered the perturbed BMC model as defined in Sect. 3.1.2. The precise setup can be found in (Van Werde et al. 2023, Supplement 2).

**Fig. 4:**
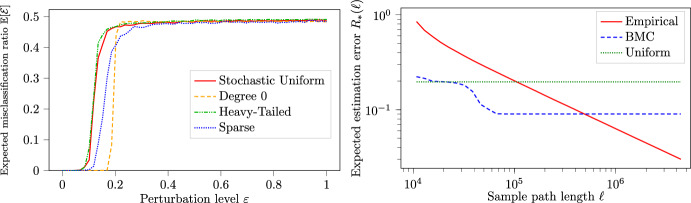
**a** The fraction of misclassified states in terms of $\varepsilon$ for various perturbation models $\Delta$. Here, $\ell _n = \lfloor 30 n\ln (n)\rfloor$ and $n=500$. **b** Estimation error $R_{*}(\ell ):= \mathbb {E}[ \Vert P - \hat{P}_{*}(\ell ) \Vert _{} ]$ in terms of $\ell$ for three different estimators and data from a perturbed BMC with $\varepsilon = 0.05$ and $n=1000$. In red: the empirical estimator $\hat{P}_{\text {Empirical}}$ which is the maximum likelihood estimator for a Markov chain with no additional assumptions. In blue: the BMC estimator $\hat{P}_{\text {BMC}}$. In green: the trivial estimator $\hat{P}_{\text {Uniform},ij}:=1/n$ which does not even use the data

Concerning (1), we find that for small perturbation levels $\varepsilon$ it is still possible to exactly recover the underlying clusters; see Fig. 4a.

Concerning (2), we consider the scenario where the goal is to estimate the transition kernel *P* of the Markov chain given a sample path of length $\ell$; see Fig. 4b. We find that clustering worsens performance when $\ell$ is large because a lack of expressivity: the true kernel *P* is not exactly a BMC-kernel. On the other hand, when $\ell$ is small, clustering improves performance because the simplified model makes the estimator less prone to overfitting. The answer to (2) is thus that it can be advantageous to rely on the BMC model assumption when data is sparse.

### Clustering algorithm

In this section we describe the clustering algorithm from (Sanders et al. 2020) which was designed to infer the map $\sigma _n$ from the sample path of a BMC. The reason we use this particular clustering algorithm is that it has a mathematical guarantee that it can recover the clusters of BMCs accurately *even if* the number of observations $\ell$ is small compared to the number of possible transitions $n^2$. This is useful for our purposes because observations are generally noisy and few in practice.

The clustering algorithm in (Sanders et al. 2020) first constructs an *empirical frequency matrix* $\hat{N}$ element-wise from the sequence of observations $X_{1:\ell }$: for $i,j \in [n]$,

8 $$\begin{aligned} \hat{N}_{ij}:= \sum _{t=1}^{\ell -1} \mathbbm {1} [ X_t = i, X_{t+1} = j ] . \end{aligned}$$

Depending on the *sparsity* of the frequency matrix characterized by the ratio $\ell /n^2$, regularization is applied by *trimming*: all entries of rows and columns of $\hat{N}$ corresponding to a desired number of states with the largest degrees, which we denote by $\Gamma$, are set to zero. The clustering algorithm then executes two steps on the resulting *trimmed frequency matrix* $\hat{N}_\Gamma$:

Step 1. Use a spectral algorithm to find an initial approximate cluster assignment.
Step 2. Iteratively improve the assignment with a cluster improvement algorithm.

We provide pseudocode for these algorithms in (Van Werde et al. 2023, Supplement 1).

Given some initial guess, here provided by a spectral algorithm, the cluster improvement algorithm consists of local optimization of a log-likelihood function by a hill climbing procedure. The state space [*n*] and the number of clusters $K$ are kept fixed which means that the free parameters are the cluster transition matrix $p \in \{ q \in [0,1]^{K\times K}: \forall k, \sum _l q_{k,l} = 1 \}$ and the cluster assignment map $\sigma _n: [n] \rightarrow [K]$. Given an observation sequence $X_{1:\ell }$, the log-likelihood of the BMC model is given by

9 $$\begin{aligned} \hat{\mathcal {L}} ( X_{1:\ell } \mid p, \sigma _n ):= \sum _{t=1}^{\ell -1} \ln { \frac{ p_{X_t,X_{t+1}} }{ \# \mathcal {V}_{\sigma _n(X_{t+1})} } }. \end{aligned}$$

The reason to use this two-step procedure instead of direct likelihood maximization is that finding the global maximizer of (9) is numerically infeasible. That hill climbing, which is computationally tractable, succeeds at exactly (resp. accurately) recovering the true parameters when initialized with a spectral clustering is formally established in (Sanders et al. 2020) in the asymptotic regime where $\ell = \omega (n\log n)$ (resp. $\ell = \omega (n)$).

### Methods for evaluating clusters and models

To interpret clustering results and assess model adequacy in the absence of a known ground truth clustering, we require principled evaluation methods tailored to sequential data. We use multiple methods and here provide short summaries; the details are given in (Van Werde et al. 2023, Supplement *Methods for evaluating clusters and models*).

***Performance on a downstream task.*** Clustering can serve as a means of dimensionality reduction when applying computational methods to sequences of observations $X_{1:\ell }$ with a large number of distinct states *n*. A clustering $\sigma _n: [n] \rightarrow [K]$ reduces the effective size of the state space to $K\ll n$, enabling more efficient or more robust downstream computations. To evaluate whether the clustering preserves relevant information, we consider a downstream task $T(X_{1:\ell })$ with an associated quality measure *Q*, such as prediction accuracy. Let $Q_{\text {pre-reduction}}:= Q(T(X_{1:\ell }))$ and $T(\sigma _n(X_{1:\ell }))$ denote the task output after clustering, with quality $Q_{\text {reduced}}:= Q(T(\sigma _n(X_{1:\ell })))$. The comparison between $Q_{\text {pre-reduction}}$ and $Q_{\text {reduced}}$ provides a concrete proxy for how much useful information is retained through clustering. In some cases, $Q_{\text {reduced}}$ may even exceed $Q_{\text {pre-reduction}}$ due to noise reduction in the clustered sequence. This method enables the empirical comparison of different clusterings and motivates clustering when the downstream task is numerically intensive or sensitive to overfitting.

***Model selection with validation data.*** To compare two candidate models $\mathbb {P}$ and $\mathbb {Q}$ for an observed sequence $x_{1:\ell }$, we consider a rescaled log-likelihood ratio

10 $$\begin{aligned} \hat{D}(x_{1:\ell }; \mathbb {P}, \mathbb {Q}):= \frac{1}{\ell } \ln \frac{\mathbb {P}[X_{1:\ell }= x_{1:\ell }] }{\mathbb {Q}[X_{1:\ell } = x_{1:\ell }]}. \end{aligned}$$

This ratio estimates the Kullback–Leibler (KL) divergence rate difference and quantifies how much more likely an observed path is under model $\mathbb {P}$ than model $\mathbb {Q}$. To reduce the bias, we use a holdout method. Specifically, we will split the trajectory into two parts: the first half $x_{1:\lfloor \ell /2 \rfloor }$ will be used for training, and the second half $x_{\lfloor \ell /2 \rfloor +1:\ell }$ for validation. We then use the holdout-based estimate

11 $$\begin{aligned} \hat{D}( x_{\lfloor \ell /2 \rfloor +1:\ell }; \hat{\mathbb {P}}^{X_{1:\lfloor \ell /2 \rfloor }}, \hat{\mathbb {Q}}^{X_{1:\lfloor \ell /2 \rfloor }} ), \end{aligned}$$

which reduces the amount of bias when compared to the estimator a standard KL divergence estimator.

**Model selection with only training data.** When validation data is unavailable or data is sparse, we assess model complexity using information criteria rather than held-out performance. Specifically, we estimate the order *r* of a $K$-state BMC (recall Sect. 3.1.2) from the clustered sequence $Y_{1:\ell } = \sigma _n(X_{1:\ell })$, and compare *r*th-order models via the Consistent Akaike Information Criterion (CAIC) (Bozdogan 1987): for model $\hat{\mathbb {Q}}^{r,\textrm{MLE}}$,

12 $$\begin{aligned} \textrm{CAIC}(\hat{Q}^{r,\textrm{MLE}}) := -2 \ln { \bigl ( \mathcal {L}( Y_{1:\ell } \mid \hat{Q}^{r,\textrm{MLE}} ) \bigr ) } + 2 \textrm{DF}(K,r) \bigl ( 1 + \ln { ( \ell - r ) } \bigr ) ; \end{aligned}$$

see (Van Werde et al. 2023, Equation (13)) for the details. Here, $\textrm{DF}(K,r)$ denotes the degrees of freedom in an *r*th-order MC constrained to have fixed parameters $K$ and *r*. Each candidate model $\hat{\mathbb {Q}}^{r,\textrm{MLE}}$ is fit by maximum likelihood to obtain a transition matrix $\hat{Q}^{r,\textrm{MLE}}$, and evaluated using a penalized log-likelihood that accounts for model complexity via $\textrm{DF}(K, r) = K^r(K- 1)$. The selected order $r^{\textrm{CAIC}}$ minimizes the CAIC and balances goodness–of–fit with parsimony. This approach allows us to detect under- or overfitting while avoiding bias due to overparameterization in the absence of explicit data splitting.

***The shape of spectral noise for identification of alternative models.*** Theory in the BMC model predicts that the leading $K$ singular values of the empirical frequency matrix $\hat{N}$ reflect the signal, while the remaining $n-K$ singular values can be interpreted as noise (Sanders and Senen-Cerda 2023; Sanders and Van Werde 2023). The dependence of this noise profile on the structure of the BMC is characterized in (Sanders and Van Werde 2023). We can use this as a model evaluation tool: we can visualize the empirical spectral noise as a histogram and compare with theory.

However, we found that the spectrum of $\hat{N}$ can be misleading as it tends to be dominated by the effect of an inhomogeneous equilibrium distribution which is common in real-world data. To address this, we instead examine the *empirical normalized Laplacian* $\hat{L}$, defined element-wise by

13 $$\begin{aligned} \hat{L}_{ij} := {\left\{ \begin{array}{ll} \frac{\hat{N}_{ij}}{\sqrt{\sum _{k=1}^n \hat{N}_{ik}}\sqrt{\sum _{k=1}^n \hat{N}_{kj}}} & \text {if } \hat{N}_{ij} \ne 0, \\ 0 & \text {otherwise.} \\ \end{array}\right. } \end{aligned}$$

We characterize the spectral noise profile for this matrix in the supplementary materials (Van Werde et al. 2023, Proposition 1) and expect it to be more robust to equilibrium imbalances. This provides a complementary, unsupervised tool for diagnosing model mismatch and identifying that richer structures may be present without an explicit alternative model.

## Experimental setup

### Data sets and preprocessing

We here introduce the data sets and our preprocessing; see Table 1 for a summary. The empirical frequency matrices resulting from this preprocessing, and examples of preprocessed trajectories are made available in the supplementary materials.

***Sequence of animal positional data***

We use data from the “Dunn Ranch Bison Tracking Project” (Stephen Blake 2017, #8019591) that provides GPS animal movement data as a sequences of latitude-longitude coordinates; recall Fig. 1. For example, the data of one animal starts as follows:

$$\begin{aligned} ( 40.4749, -94.1129) \rightarrow ( 40.4748, -94.1130) \rightarrow ( 40.4749, -94.1129) \rightarrow \ et\, cetera . \end{aligned}$$

The study provides data from 24 animals which we concatenated to a single observation sequence. As preprocessing, we also excluded some outlier GPS coordinates outside a rectangular ${3.2\,\mathrm{\text {k}\text {m}}} \times {1.7\,\mathrm{\text {k}\text {m}}}$ region caused by malfunctions of the tracking device.

If we assume that every GPS coordinate yields a distinct state of a BMC, then clustering would be infeasible because there would be as many states as observations. We therefore combine GPS coordinates by binning over a grid of squares with width ${0.04\,\mathrm{\text {k}\text {m}}}$, chosen by ad-hoc parameter tuning; see (Van Werde et al. 2023, Supplement 5.2) for details. After preprocessing and binning, the sequence becomes

$$\begin{aligned} X_1 = \text {Bin 0} \rightarrow X_2 = \text {Bin 1} \rightarrow X_3 = \text {Bin 0} \rightarrow X_4 = \text {Bin 0} \rightarrow et\, cetera . \end{aligned}$$

We finally eliminated self-jumps such that resting animals do not disturb the findings. We end up with $n=3155$ states and a sequence of length $\ell =193134$.

***Sequence of codons in DNA***

A string of DNA can be viewed as a sequence composed of four possible nucleotides, denoted A, T, C, and G. These are processed in protein synthesis in three-letter words called *codons*. For instance, the codon ACG corresponds to addition of the amino acid threonine as the next building block of a protein. Given a sequence of nucleotides like

$$\begin{aligned} \text {TTTGTAGTTAGATCTCCTCTATCC} et\, cetera , \end{aligned}$$

it is hence natural to focus on the associated sequence of codons:

$$\begin{aligned} X_1 = \text {TTT} \rightarrow X_2 = \text {GTA} \rightarrow \cdots \rightarrow X_8 = \text {TCC} \rightarrow et\, cetera . \end{aligned}$$

We consider data from the OCA2 gene in human DNA (National Library of Medicine 2021). The specific gene is merely illustrative: the clustering algorithms can be applied to any gene, and we expect similar results. We find $\ell = 16 \times 10^4$ transitions and a state space of size $n = 64$.

***Sequence of words in texts*** A cleaned corpus based on the Wikipedia datadump of October 2013 was downloaded from (Wilson 2015). Further preprocessing was standard: we removed all punctuation and numbers, reduced to a root word with the Natural Languages Toolkit’s PorterStemmer.stem() (Bird et al. 2009, Section 3.6) and pruned the 100 most used words and words with fewer than 1000 occurrences. For example, a paragraph such as

$$\begin{aligned} \text {Clustering observations can be very useful! } \end{aligned}$$

is converted into the sequence

$$\begin{aligned} X_1 = \text {cluster} \rightarrow X_2 = \text {observ} \rightarrow \cdots \rightarrow X_{6} = \text {use}. \end{aligned}$$

Each *s*th Wikipedia page results in a sequence that is relatively short. The corresponding frequency matrix $\hat{N}^{s}$, recall (8), is hence excessively sparse. We therefore compute and work instead with $\hat{N} := \sum _s \hat{N}^{s} .$ The diagonal of the matrix is further set to zero because self-transitions are common and not particularly informative for the purpose of clustering. Pruning these removes a potential bias towards homophilic clusters. We end up with a vocabulary of $n=16994$ words and $\ell \approx 2 \cdot 10^8$ transitions.

***Sequence of companies with the highest daily return***

Daily pricing data for every company in the S&P500 index was downloaded from (Alpha Vantage Co 2021). The data did not span the same time range, so we only retained the 300 companies with the most complete data. We determined the times $t_-^{i}$ and $t_i^+$ of the first and final data entry of each constituent consider the time range from $t_0 := \max _{i \le 300}t_-^{i}$ to $t_0 +\ell := \min _{i \le 300} t_+^{i}$. It turned out that $t_0 = \text {2001--07--26}$ and $t_0 + \ell = \text {2021--10--22}$. Days without data, such as weekends when the market is closed, were ignored.

Let $O_t^{i}$ and $C_t^i$ denote the opening price and closing price of company *i*’s stock on day *t*, respectively. We considered the company with the highest daily return:

14 $$\begin{aligned} X_t \in \operatorname {argmax}\limits _{i \le 300} C_t^i / O_t^i \end{aligned}$$

The resulting sequence of company tickers starts with

$$\begin{aligned} X_{t_0} = \text {ADI} \rightarrow X_{t_0+1} = \text {AES} \rightarrow X_{t_0 + 2} = \text {PVH} \rightarrow \cdots . \end{aligned}$$

We again eliminate self-jumps and end up with $\ell \approx 24\times 10^2$ transitions on a state space of size $n=300$.

**Table 1:** Summary of the used datasets. The final two columns are only approximations showing the order of magnitude

Dataset	#States *n*	#Transitions $\ell$	Visits per state $\ell /n$	Sparsity $\ell /n^2$
Codons in DNA	64	$16\times 10^4$	2500	40
Animal movements	3155	$19\times 10^4$	60	0.02
Words in text	16994	$2\times 10^8$	10000	0.7
Companies S&P500	300	$2\times 10^3$	8	0.3

### Implementation description for BMCToolkit

To tackle large sequences of observations, we programmed a DLL in C++ that can simulate and analyze trajectories of BMCs. Among other functionalities, the DLL is able to calculate both cluster and state variants of the equilibrium distribution, frequency matrix, and transition matrix of a BMC; to compute the difference between two clusters and the spectral norm; to estimate the parameters of a BMC from a sample path; to execute the spectral clustering algorithm and the cluster improvement algorithm; to generate sample paths and trimmed frequency matrices; and to relabel clusters according to the size or the equilibrium probability of a cluster.

The DLL utilizes *Eigen*, a high-level DLL for linear algebra, matrix, and vector operations; and the *ARPACK*, a DLL for large-scale eigenvalue problems built on top of Eigen. The mathematical components of BMCToolkit were validated through functional testing using Microsoft’s Native Unit Test Framework. The performance of the numerical components of BMCToolkit were finally benchmarked using *Benchmark*, Google’s microbenchmark support library. Our source code can be found at https://gitlab.tue.nl/acss/public/detection-and-evaluation-of-clusters-within-sequential-data.

We also created a Python module called *BMCToolkit*, and made it available at https://pypi.org/project/BMCToolkit/. This Python module distributes the DLL mentioned above and includes an easy-to-use Python interface. When compiling BMCToolkit, we made sure to instruct the Microsoft Visual C++ compiler to activate the *OpenMP* extension to parallelize the simulation across Central Processing Units and so that Eigen could parallelize matrix multiplications (/openmp); to apply maximum optimization (/O2); to enable enhanced Central Processing Unit instruction sets (/arch:AVX2); and to explicitly target 64-bit x64 hardware.

## Results

We now evaluate how well the BMC model can capture the structure of the sequential data introduced in Sect. 4.1 and if it can yield useful insights. Specifically, the detected clusters and our findings are discussed in Sect. 5.1, and we study what order of the MC best fits the data in Sect. 5.2.

### Detected clusters within the data

#### Animal movement data

We here investigate the GPS animal movement data from the *Dunn Ranch Bison Tracking Project*; recall Sect. 4.1.

***Subjective evaluation***

The results of the clustering algorithm are depicted in Fig. 2. It is subjectively evident that the clusters give more insight than the scatter plot in Fig. 1.

Observe that the clustering algorithm picks up on geographical features: all clusters are connected regions, except for the largest two clusters 1 (black dots) and cluster 2 (orange *c*’s). Clusters 1 and 2 contain the low degree states which explains their geographical spread. For the other clusters geographical boundaries are visible. For example, cluster 3 is bounded from below by creeks and cluster 4 lies between two creeks. On satellite imagery one can see a fence north of 7 and the part of 2 that is bordering 7 and in fact, the northern border of these two clusters follows that line.

Let us emphasize that the fact that the clusters respect the underlying geography and barriers is a nontrivial observation: the clustering algorithm identifies states by numbers and does *not* use geographical information on the state labeling. The labels of the states are in fact arbitrary to the algorithm, states labeled e.g. 10 and 11 need not be close to each other geographically. Hence, geographically mixed clusters would also have been a valid outcome of the algorithm.

Let us note that the average rate of transitions within each cluster is 0.79. The transitions shown on the map thus do not represent the majority of transitions, but only the transitions between different clusters that occur with probability of at least 0.01. The cluster transitions matrix is given in (Van Werde et al. 2023, Supplement 6.3).

***Comparing the histogram of singular values to the limiting distribution of singular values of the inferred BMC***

**Fig. 5:**
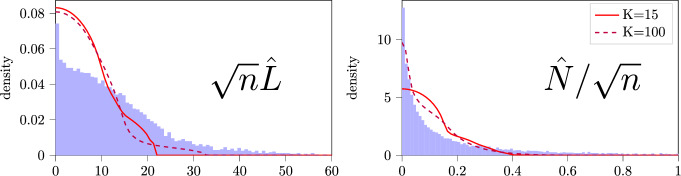
Density-based histogram of singular values for $\sqrt{n}\hat{L}$ and $\hat{N}/\sqrt{n}$ for the animal movement data in blue bars and the theoretical predictions associated with the improvement clustering with $K= 10$ as the red line and with $K=100$ as the purple dashed line

Fig. 5 next compares the spectral noise of (8) and (13) to the theoretical predictions for BMCs (see Van Werde et al. 2023, Proposition 1). Observe that with $K= 15$ clusters, the theoretical prediction captures the general shape of the distribution, but is inaccurate for the smallest and largest singular values especially. With more clusters, $K=100$, the theoretical prediction for the distribution of singular values is found to predict the distribution of singular values better across the entire range. The prediction however remains imperfect. The peak at zero is probably linked to the fact that there are many states with a low degree.

***Conclusion***

We conclude that a BMC is a useful model for describing animal movement data. In fact, surprisingly, the clustering algorithm manages to deduce underlying geographical information (such as regions, barriers, and movement patterns) from the mere time dependency within the observation sequence. Because of this visuo-spatial ability, the algorithm may have a potential use as a tool for spatial recognition.

At the same time however, we also conclude that a BMC does not describe the underlying complex process in its entirety. For example, the distribution of singular values depicted in Fig. 5 is not predicted perfectly. This is likely caused by the symmetry assumption between states within a BMC, which is at odds with the geographical structure of the data. Indeed, if we cut the region into more but smaller clusters and thus reduce the amount of symmetry within the BMC modeling the observation sequence, the BMC’s prediction of the distribution of singular values improves.

#### Sequence of codons in DNA

We consider the sequence of codons occurring in the gene OCA2 in human DNA. The detected clusters are displayed in Table 2.

**Table 2:** The detected clusters of codons in a short sequence of human DNA. Observe that many codons in $k = 2$ end with *C*, and that all codons in $k = 3$ start with *G*

*k*	Codons within detected cluster *k*
1	AAA, AAG, TGT, AGT, CCT, TCT, ACT, CAG, ATT, ATG, CAT, TAT, AAT, TTG, CTT, TGA, CTG, CAA, TGG, ATA, TTA, AGG, TAA, ACA, TCA, CCA, AGA
2	CAC, GCC, CCC, TCC, ACC, GTC, CTC, TTC, ATC, TGC, AGC, TAC, AAC, GGC, TAG, CTA, GAC
3	GTG, GAG, GGT, GCA, GAA, GTA, GGA, GAT, GGG, GTT, GCT
4	CGA, CGC, ACG, TCG, CCG, GCG, CGT, CGG
5	TTT

**Fig. 6:**
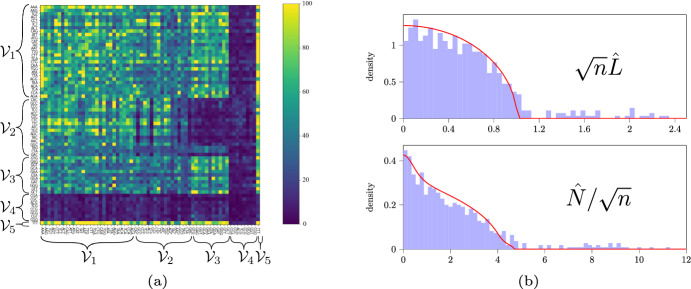
**a** The frequency matrix $\hat{N}$ when the codons are sorted by the five detected clusters. **b** Average density-based histogram of singular values for $\sqrt{n}\hat{L}$ and $\hat{N}/\sqrt{n}$ for the DNA sequential data in blue bars and the theoretical predictions associated with the improved clustering as the red line. Not displayed is that each observation of $\hat{N}/\sqrt{n}$ also has a single singular value near 40 and each observation of $\sqrt{n}\hat{L}$ has a single singular value near 8. These extremal singular values are considered to be part of the signal, and consequently not relevant for measuring the spectral noise

***Possible detection of codon–pair bias*** The frequency matrix, displayed after clustering, reveals an interesting pattern; see Fig. 6a. We observe that all rows and columns associated with the second-to-last cluster $\mathcal {V}_4$ have low density. This means that the states in $\mathcal {V}_4$ have small equilibrium distribution. More interesting is the low-density block in the rows and columns corresponding to the transitions from $\mathcal {V}_2$ to $\mathcal {V}_3$. It appears we have rediscovered a phenomenon known as *codon–pair bias* in biology (Gutman and Hatfield 1989; Coleman et al. 2008; Kunec and Osterrieder 2016).

There is some evidence that codon–pair bias is nothing more than a consequence of *dinucleotide bias* (Kunec and Osterrieder 2016). Here, the term dinucleotide bias refers to the fact that the two-letter pair $\text {CG}$ is used infrequently regardless of its position. This dinucleotide bias can also explain the clusters observed in Fig. 6a. Indeed, inspection of the clusters $\mathcal {V}_1, \ldots , \mathcal {V}_5$ reveals that nearly all codons in $\mathcal {V}_2$ end with the nucleotide $\text {C}$ whereas all codons in community $\mathcal {V}_3$ begin with nucleotide $\text {G}$. There are a few exceptions, the codons TAG and CTA in $\mathcal {V}_2$, but visual inspection of $\hat{N}$ suggests that these may have been misclassified. Thus, transitions from $\mathcal {V}_2$ to $\mathcal {V}_3$ would give rise to the two nucleotides $\text {CG}$ on the interface. Also remark that the two leftmost vertical low-density streaks in the block associated with $\mathcal {V}_2$ correspond to codons GCC and GTC which simultaneously begin with a $\text {G}$ and end with a $\text {C}$. Finally, all codons in $\mathcal {V}_4$ contain the two nucleotides $\text {CG}$. It thus appears that all low-density regions in the figure could be explained through dinucleotide bias. We refer to (Alexaki et al. 2019) and the references therein for further discussions of codon–pair bias, dinucleotide bias and their applications.

***Comparing the histogram of singular values to the limiting distribution of singular values of the inferred BMC***

It appears from the reasonable clusters in Fig. 6a that a BMC could be an appropriate model for this dataset. Let us now additionally verify whether the shape of the spectral noise is consistent with a BMC. Note that the matrices $\hat{N}$ and $\hat{L}$ are only $64 \times 64$. Consequently, they only have 64 singular values. To get a clearer picture we split the observation sequence into ten equally sized pieces and for each subpath we compute the singular values. The averaged histogram over these ten observations is compared to the theoretical BMC-prediction associated to the clusters in Fig. 6b.

We observe a good match to the theory for both $\hat{N}$ and $\hat{L}$. Particularly interesting is the peak near zero and the triangular tail in the interval [4, 5]. The theoretical there matches the observed distribution for $\hat{N}/\sqrt{n}$. Such features would not be predicted in a simpler model without communities such as a matrix with i.i.d. entries. One would then instead expect a quarter-circular law with density proportional to $\mathbbm {1} [ x\in (0,c) ] \sqrt{c^2 - x^2}$ for some $c>0$. This quarter-circular law is observed in the empirical Laplacian $\hat{L}$ suggesting that the main feature in the spectral noise of $\hat{N}$ is due to the equilibrium distribution. There are also some singular values which escape the support of the limiting singular value distribution. These are most-likely associated to the signal $\mathbb {E}[\hat{N}]$ and should consequently not be viewed as a part of the spectral noise.

***Conclusion***

It appears that the clustering algorithm was able to detect the phenomena of dinucleotide bias in DNA. The spectral noise is consistent with a BMC and a simpler model generating a random matrix with independent and identically distributed entries would not have sufficed to predict $\hat{N}$’s singular values.

#### Sequence of words on wikipedia

The clustering algorithm discussed in Sect. 3.2 was executed for $K= 50, 100, 200, 400$, both with and without the improvement algorithm. Ten improvement iterations were done whenever we used the latter algorithm. A complete list of the clusters for $K=200$ with improvement is given in (Van Werde et al. 2023, Supplement 6.2).

***Subjective evaluation*** At a first glance, the found clusters appear meaningful. For instance, a small cluster with six elements has a distinctly football-related theme: $\mathcal {V}_{125}$ contains the words *champion*, *cup*, *premier*, *coach*, *footbal* and *championship*. The medium-sized clusters $\mathcal {V}_{50}$, $\mathcal {V}_{51}$, and $\mathcal {V}_{52}$ respectively contain words related to public professions, units, and warfare. That is, $\mathcal {V}_{50}$ includes stemmed words such as *founder*, *deputi*, *formeli*, *mayor*, *bishop*, *meanwhil*, *successor*, $\mathcal {V}_{51}$ includes *tonn*, *usd*, *capita*, *lb*, and $\mathcal {V}_{52}$ includes *cavalri*, *jet*, *helicoptr*, *rifl*, *warfar*, *battalion*, and *raid*. The second-largest cluster $\mathcal {V}_2$ predominantly contains names, including *alexandr*, *albrecht*, *gideon*, and *jarrett*.

We further observe that the improvement algorithm yields more balanced clusters: before the improvement algorithm the largest three clusters have sizes 9192, 1279 and 1126, respectively, while after improvement the sizes are 2848, 1943 and 1600.

***Performance on a downstream task*** To evaluate the quality of the clusters more objectively, we investigate the performance achieved on a downstream task as discussed in Sect. 3.3.

We specifically consider a document classification task where the goal is to predict the label *l*(*d*) of a document *d* given some training dataset. The considered datasets are described in (Van Werde et al., 2023, Supplement 6.2). For instance, the AG News dataset contains news articles with four possible labels: *World*, *Sports*, *Business*, and *Sci/Tech*.

Given a clustering, one can translate each document into a $K$-dimensional vector by counting the number of occurrences of each cluster in the document; see (Van Werde et al. 2023, Supplement 5.1). Thereafter, a logistic regression model is trained to learn a mapping from the $K$-dimensional vectors to the labels. Aside from spectral and improvement clusters we also consider a random clustering in which every word is assigned a cluster uniformly at random. There were some datasets in which neither spectral nor improvement clustering significantly outperformed the random clustering. We consider these tests inconclusive, but report on them in (Van Werde et al. 2023, Supplement 6.2) for completeness. The performance on the remaining datasets is displayed in Table 3.

Observe that improvement clustering typically outperforms plain spectral clustering. Further, in the *AG News*, *Yahoo!* and *Wiki* datasets the performance increases with the dimensionality. The gain in performance from spectral and improvement clustering as opposed to random clustering is there comparable with an increase of dimensionality by a factor 4. On the other hand, for *Books* and *CMU* it appears that the performance decreases with the dimensionality, although this pattern is less clear. A possible explanation is that *Books* and *CMU* have less training data so that overfitting may occur when the dimensionality is large.

**Table 3:** Performance of clustering before and after improvement as measured by accuracy in the downstream task of document classification as compared to a random clustering. Bold added for the best-performing method

$K$	Algorithm	AG News	Yahoo!	Wiki	Book	CMU
50	Random	48.3%	27.4%	56.9%	31.0%	67.4%
50	Spectral	66.0%	39.8%	71.1%	44.4%	69.5%
50	Improved	**68.5%**	**40.1%**	**71.5%**	**44.7%**	**71.8%**
100	Random	55.5%	33.3%	68.4%	30.0%	67.4%
100	Spectral	72.7%	47.2%	**81.6%**	45.2%	70.0%
100	Improved	**76.8%**	**49.0%**	80.1%	**46.3%**	**70.7%**
200	Random	64.0%	41.7%	80.8%	28.2%	66.8%
200	Spectral	78.2%	51.7%	85.6%	**44.4%**	68.7%
200	Improved	**80.7%**	**54.7%**	**86.5%**	43.4%	**69.0%**
400	Random	72.8%	49.4%	87.8%	28.9%	66.8%
400	Spectral	81.5%	56.3%	88.0%	42.1%	67.9%
400	Improved	**83.1%**	**58.6%**	**89.0%**	**44.4%**	**68.4%**

***Comparing the histogram of singular values to the limiting distribution of singular values of the inferred BMC***

**Fig. 7:**
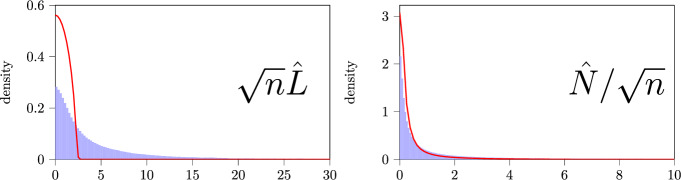
Density-based histogram of singular values for $\sqrt{n}\hat{L}$ for the words sequential data in blue bars and the theoretical predictions associated with the improvement clustering with $K= 200$ as the red line. Not visible in this figure is that both empirical distributions have long tails. Still $9\%$ of the singular values of $\hat{N}/\sqrt{n}$ exceed 10 and $1\%$ of the singular values of $\sqrt{n}\hat{L}$ exceed 30

One may be tempted to deduce from the reasonable clusters and the performance in Table 3 that the BMC model is appropriate for this dataset. The structure in the spectral noise is however not as one would expect. Consider Fig. 7 for a comparison of the empirical singular value distribution with the theoretical predictions. Observe that there is a good match for $\hat{N}$ but a discrepancy for $\hat{L}$.

The fact that $\hat{N}$ yields a good match can be explained as being due to a strongly inhomogeneous equilibrium distribution from Zipf’s law. The empirical Laplacian $\hat{L}$ removes this dominant effect after which it may be observed that the empirical distribution has a heavy tail which is not present in the BMC-based prediction. In (Van Werde et al. 2023, Supplement 6.4) we demonstrate by a numerical example that the discrepancy which is observed in Fig. 7 agrees precisely with the type of discrepancy which is observed for a heavy-tailed perturbation of the BMC. The fact that the entries of the matrices $\hat{N}$ and $\hat{L}$ are heavy-tailed may also be verified by direct inspection.

***Conclusion*** The clustering algorithm found clusters that we judge to be meaningful. The performance on a downstream document classification task further indicated that the improvement algorithm based on the BMC-assumption improved the quality of the clusters. The spectral noise indicated that there is some heavy-tailed component in which can not be accounted for by BMCs. It is hence conceivable that a different model could incorporate the heavy-tailedness and extract even better clusters.

#### Companies with the highest daily returns

We finally turn to the sequence of companies with the highest daily returns. This analysis was particularly delicate to conduct and we ultimately arrive at the conclusion that a 0th-order BMC could already be sufficient to explain the found clusters.

This conclusion may appear disappointing: it means that the clusters may not encode order-dependent dynamics. It is however important for a practitioner to be able to arrive at this conclusion when appropriate. The fact that the evaluation methods from Sect. 3.3 are able to suggest a 0th-order BMC is correspondingly a good feature: the method would not be informative in the alternative scenario where one always concludes in favor of the 1st-order BMC. The main goal of this section is hence to demonstrate how the methods can be used in a difficult, sparse, regime.

There are two main reasons why this dataset is difficult to analyze. First, the data is sparse; recall from Table 1 that $\ell / n^2 \approx 0.03$ and $\ell /n \approx 8$. This sparsity makes recovery of the clusters a hard problem, even if the data-generating-process is truly a BMC, and moreover makes evaluation of the found clusters more difficult since the associated confidence bounds are large. Second, it turns out that the data contains a strong 0th-order component which could potentially serve as a nuisance factor, concealing a 1st-order BMC component even if it exists.

***Subjective evaluation of the clusters***

After some *ad hoc* experimentation, we fix $K= 3$. The S&P500’s factsheet labels every constituent with a sector; see (Van Werde et al. 2023, Supplement 6.3). We can use this labeling to obtain “fingerprints” of clusters.

**Fig. 8:**
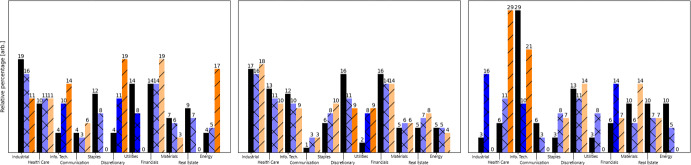
The fraction of constituents in each sector for models $\hat{\mathbb {P}}, \hat{\mathbb {Q}}_1$ and $\hat{\mathbb {Q}}_2$ as the black, blue and orange bars respectively. The left, middle, and right plots correspond to the 1st largest, 2nd largest, and 3rd largest detected cluster, respectively. A bar’s color is saturated when the difference in relative percentage exceeds $5\%$ when compared to the black bars

The black bars in Fig. 8 show the relative percentages of constituents in each sector for the clusters found after the improvement algorithm. Observe the absence of most utilities constituents within the 2nd and 3rd cluster; more than twice as many are assigned to the 1st cluster than may be expected in a random assignment. Industrial and health care constituents are also mostly absent within the 3rd cluster. Similarly, note the negligible number of consumer discretionary constituents within the 1st cluster; most are assigned to the 2nd and 3rd cluster. Finally, consider that the 3rd cluster consists for $29\%$ out of information technology constituents. These contents suggest that the clusters are not entirely random. The subsequent experimentation aims to determine what type of information has been encoded in the clusters.

As a subjective way to evaluate the meaning of the clusters, let us inspect the relative cluster sizes $\hat{\alpha }_k := \# \hat{\mathcal {V}}_k/n$, cluster equilibrium distribution $\hat{\pi }$, and cluster transition matrix $\hat{p}$ of the associated BMC:

$$\begin{aligned} \hat{\alpha }^{\textrm{T}} \approx \begin{pmatrix} 0.45 \\ 0.45 \\ 0.10 \\ \end{pmatrix}, \; \hat{\pi }^{\textrm{T}} \approx \begin{pmatrix} 0.49 \\ 0.10 \\ 0.41 \\ \end{pmatrix}, \; \hat{p} \approx \begin{pmatrix} 0.50 & 0.10 & 0.40 \\ 0.54 & 0.11 & 0.35 \\ 0.46 & 0.10 & 0.44 \\ \end{pmatrix}. \end{aligned}$$

Note that the rows of $\hat{p}$ are close to but not quite equal; it namely holds that $\hat{p}_{kl} \approx \hat{\pi }_l$ for every *k*, *l*. This observation may suggest a strong 0th-order BMC component. One can however not immediately conclude that all the deviations from constant columns are due to noise: the data is sparse relative to $n^2$ but not when compared to $K^2 = 9$.

***Comparing against alternative models***

**Fig. 9:**
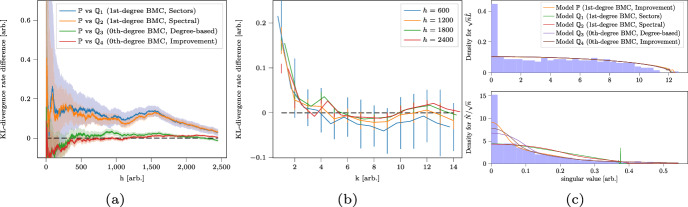
**a** The KL divergence rate difference estimator $D(X_{ \lfloor \ell / 2 \rfloor + 1: \lfloor \ell / 2 \rfloor + h }; \hat{\mathbb {P}}^{X_{1:\lfloor \ell / 2 \rfloor }}, \hat{\mathbb {Q}}_i^{X_{1:\lfloor \ell / 2 \rfloor }})$ on the validation data with 95% confidence bounds estimated using (Van Werde et al. 2023, Supplement 3, Eq. (13)) with mixing time (arbitrarily) guessed to be 20 days. **b** The KL divergence rate difference estimator $\hat{D}(X_{\lfloor \ell / 2 \rfloor + 1:\lfloor \ell / 2 \rfloor + h}, \hat{\mathbb {P}}^{X_{1:\lfloor \ell / 2 \rfloor }}, \hat{\mathbb {Q}}_{3,k}^{X_{1:\lfloor \ell / 2 \rfloor }})$ for different sample path lengths $h \in \mathbb {N}_{+}$, and as a function of *k* with 95% confidence bounds using (Van Werde et al. 2023, Supplement 3, Eq. (13)). **c** The top and bottom figures display the singular values of $\sqrt{n}\hat{L}$ and $\hat{N}/\sqrt{n}$ respectively. Both figures exclude the $K=3$ leading singular values

Recall that, using validation data, we can compare the performance of different models by the KL divergence rate difference estimator (11). Consider the following models:

$\hat{\mathbb {P}}$: A 1st-order BMC with $K = 3$ clusters found by the spectral algorithm followed by the improvement algorithm.
$\hat{\mathbb {Q}}_1$: A 1st-order BMC with $K = 11$ clusters given by the sector labels.
$\hat{\mathbb {Q}}_2$: A 1st-order BMC with $K = 3$ clusters, found by the spectral algorithm.
$\hat{\mathbb {Q}}_3$: A 0th-order BMC with $K = 3$ clusters, found by sorting along the state’s sample equilibrium distribution and determining clusters of equal probability mass.
$\hat{\mathbb {Q}}_4$: A 0th-order BMC with $K = 3$ clusters, found by the spectral algorithm followed by an improvement algorithm (modified for a 0th-order BMC).

One may also wonder about the effect of the number of parameters. By keeping the number of clusters fixed, it namely follows that the 0th-degree models $\hat{\mathbb {Q}}_2,\hat{\mathbb {Q}}_3$ have fewer parameters than the 1st-degree models $\hat{\mathbb {P}},\hat{\mathbb {Q}}_4$. Hence, consider the following model for any $k\ge 1$:

$\hat{\mathbb {Q}}_{3,k}$: A 0th-order BMC with *k* clusters, found by sorting according the state’s sample equilibrium distribution and determining *k* clusters of equal probability mass.

Note that the degrees of freedom $\textrm{DF}_1(n, K)$ within a 1st-order BMC with fixed parameters $(n, K)$ equals $\textrm{DF}_1(n,K) = n + K(K-1)$, whereas the degrees of freedom $\textrm{DF}_0(n, K)$ within a 0th-order BMC constrained with fixed parameters $(n,K)$ equals $\textrm{DF}_0(n,K) = n + K- 1$. The model $\hat{\mathbb {P}}$ therefore has $n+6$ degrees of freedom whereas $\hat{\mathbb {Q}}_{3,k}$ has $n + k-1$ degrees of freedom. In particular, the degrees of freedom for $\hat{\mathbb {P}}$ and $\hat{\mathbb {Q}}_{3,7}$ are comparable. The remaining difference is that $\hat{\mathbb {P}}$ allows for more inhomogeneity within the columns of the transition matrix and less in the rows, whereas $\hat{\mathbb {Q}}_{3,7}$ allows no inhomogeneity within the columns but more in the rows. Observe in Fig. 9(a) that the difference in KL divergence rate on the validation data is positive when comparing $\hat{\mathbb {P}}$ against $\hat{\mathbb {Q}}_1$, $\hat{\mathbb {Q}}_2$, barely positive when comparing against $\hat{\mathbb {Q}}_3$, and near-zero when comparing against $\hat{\mathbb {Q}}_4$. The 0th-degree models $\hat{\mathbb {Q}}_3$, $\hat{\mathbb {Q}}_4$ perform comparable to the 1st-degree model $\mathbb {P}$.

Regarding the comparison with $\hat{\mathbb {Q}}_{3,k}$ we may observe in Fig. 9b that the sign of the KL divergence rate difference is probably positive for $k = 1, 2, 4$, possibly positive for $k = 3, 11, 12$ but not much, possibly negative for $k = 6, 7, 8$ but not much, and inconclusive for $k = 5, 9, 10$. The downward trend for small *k* suggests that a strictly positive number of free parameters are necessary to accurately represent the data. Judging from the case $k \approx 7$, in which case the number of degrees of freedom in both models are equal, it appears that the specific freedoms allowed in $\hat{\mathbb {P}}$ give a performance comparable to that attained by the freedoms allowed in $\hat{\mathbb {Q}}_{3,7}$.

***Comparing the histogram of singular values to the limiting distribution of singular values of the inferred BMC***

Fig. 9c depicts histograms of singular values and theoretical predictions for the models $\hat{\mathbb {P}}$, $\hat{\mathbb {Q}}_1$, $\hat{\mathbb {Q}}_2$, $\hat{\mathbb {Q}}_3$, $\hat{\mathbb {Q}}_4$’s. All theoretical predictions were calculated from training data, while the histograms were calculated from validation data.

All theoretical predictions give a fair description of the laws. Models $\hat{\mathbb {P}}$, $\hat{\mathbb {Q}}_4$ outperform models $\hat{\mathbb {Q}}_1$, $\hat{\mathbb {Q}}_2$, $\hat{\mathbb {Q}}_3$ when it comes to describing the distribution of singular values of $\hat{N}_{\text {validation}} / \sqrt{n}$. Observe that the empirical observations for $\sqrt{n}\hat{L}_{\text {validation}}$ as well as the predictions associated to $\hat{\mathbb {P}}$, $\hat{\mathbb {Q}}_1$, $\hat{\mathbb {Q}}_2$, $\hat{\mathbb {Q}}_3$, $\hat{\mathbb {Q}}_4$ all appear to be quarter-circular. This quarter-circular law is consistent with our suspicion of a strong 0th-degree model component: in a 0*th*-degree BMC, the limiting law of $\sqrt{n}\hat{L}$ is known to be quarter-circular. The peak at zero in the empirical observations is likely due to the sparsity.

***Conclusion***

In all considered performance measures we saw that the 1st-degree BMC model $\mathbb {P}$ performed approximately equally well as the 0th-degree models $\hat{\mathbb {Q}}_3$, $\hat{\mathbb {Q}}_4$. The consideration of the models $\hat{\mathbb {Q}}_{3,k}$ suggested that one further requires a certain number of parameters to achieve sufficient model expressivity.

The sparsity of the data makes it difficult to come to a definitive conclusion. Still, one generally prefers models with fewer parameters. Hence, in our opinion, a 0th-order BMC would be a suitable model for this dataset.

### Detected orders within the data

We investigate what order of BMC best fits the clustered data $Y_t = \sigma _n(X_t)$ using the information criteria described briefly in Sect. 3.3 (and in detail in (Van Werde et al. 2023, *Methods for evaluating clusters and models*)). We focus on the DNA, GPS, and the S&P500 dataset. The Wikipedia data is omitted due to its impractical size, and because it does not consist of a single sample path but rather a number of small sample paths.

#### Results

We compute (12) for $r = 0,1,2,3,4$ of the following models:

$\hat{\mathbb {Q}}^{r, \textrm{MLE}}$: : The Maximum-Likelihood Estimator of an *r*th-order MC estimated from the observation sequence $Y_{1:\ell }$.

The result are in Table 4. We see that the magnitude of the CAIC in Table 4 depends strongly on the observation sequence and the number of clusters. For the GPS coordinates, the differences are notable for most orders due to the large number of clusters $K=15$, where higher orders become highly penalized. For DNA, the criterion suggests that orders $r \in \{ 1, 2\}$ are optimal. For the S&P500, on the other hand, orders $r \in \{0,1\}$ appear to be the best. We expect a large variance in Table 4 and some over or underfitting the order is possible. The criterion indicates nonetheless that the transitions of the found clusters, except maybe for the S&P500 dataset, can be better approximated by a nonzero order Markovian process. We will now support this conclusion empirically with the error models for the DNA and S&P500 datasets.

**Table 4:** The CAIC in (12) for the different datasets. Note that the relative difference between the values pertaining to different orders is often small. For example, the differences are less than $0.1\%$ between orders 1, 2 for the DNA data, and between orders 0, 1 for the stock market data. This is not the case, however, with the animal data

*r*	DNA	incr. ($\%$)	GPS($\times 10^3$)	incr. ($\%$)	S&P500	incr. ($\%$)
0	432650	n.a	960.63	n.a	**9853**	**n.a.**
1	431502	$-$0.27	626.54	$-$34.8	9860	+0.07
2	**431263**	− **0.06**	**571.49**	− **40.5**	9940	+0.81
3	435228	+0.69	1121.90	+16.8	10253	+3.1
4	458512	+5.3	9789.27	+1019	11162	+8.9

To further support the accuracy of the CAIC, in (Van Werde et al. 2023, Section 6) an accuracy study of the criterion is conducted under a generative model based on the datasets. This study suggests that the criterion is less prone to overfit or choose a model with more parameters. Hence, the nonzero orders estimated in Table 4 hint that a high-order Markov structure in the data exists that a model such as the BMC can approximate.

We finally remark that using information criteria for the unclustered observation sequences $X_{1:\ell }$ provides no useful insights due to the large dimensionality of the models. In particular, the CAIC criteria for the unclustered observation sequences for order $r \in \{0, 1\}$ can be seen in Table 5. As the data shows, the CAIC criteria just picks the model with smallest number of parameters. This is even more extreme in the GPS and S&P500 datasets, where on top of large model dimension we have sparse data.

**Table 5:** The CAIC in (12) for the sequence $X_1$, $\ldots$, $X_{\ell }$ for different datasets

*r*	DNA	GPS	S&P500
0	**1339**.**5** $\times 10^3$	**2943** $\times 10^3$	**54**.**27** $\times 10^3$
1	1361.9 $\times 10^3$	$\approx$ 1 $\times 10^8$	882 $\times 10^3$

#### Conclusion

We found that model selection is feasible if we use the clustered sequence $Y_{1:\ell } = \sigma _n(X_{1:\ell })$ obtained after the clustering algorithm, because this reduces the amount of free parameters of the models considerably.

For the DNA and GPS datasets, the CAIC selects a nonzero order MCs. For the S&P500, the data was too sparse for selecting a specific order with certainty. However, there are indications that the values obtained in the CAIC for the S&P500 dataset are consistent with a 1st-order BMC model with a strong 0th-order MC baseline.

## Conclusions

We have found that using a BMC model for exploratory data analysis in unlabeled observation sequences does in fact produce useful insights. Although there is no guarantee that there are clusters or that a cluster structure is actually revealing of a ground truth model we can still evaluate the clusters and associated models. The animal movement example uncovered features which could not have been extracted from only the GPS coordinates. The DNA example uncovered known, nontrivial and biologically relevant structure. In the text-based example, the improvement algorithm enhanced performance on down-stream tasks and the spectral noise identified the heavy-tailed nature of some model violations. For the daily best performing stocks in the S&P500, we saw that a 0th-order BMC can describe its statistical aspects, but there are indications that a 1st-order BMC is also a suitable model.

## Supplementary Information

Below is the link to the electronic supplementary material.

Supplementary file 1 (pdf 706 KB)

## Data Availability

References to data sources, and processed data, are provided within the manuscript and supplementary information files.

## Footnotes

1 Inspired by the current paper’s findings, the first and final author have recently considered a different model for a stochastic process with a natural notion of barriers and studied the theory of the associated recovery problem as well as applications to animal movement data (Van Werde and Sanders 2024). Notably, however, the barrier recovery algorithms from that paper would not as easily give insight into the global topological structure of the dynamics. For such global structure, a clustering-based approach is more insightful.
