# Supplementary material for: Detection and evaluation of clusters within sequential data
Source: Data Min Knowl Discov. 2025 Aug 14;39(6):69. doi: 10.1007/s10618-025-01140-4 (PMC12354125; doi:10.1007/s10618-025-01140-4)
Supplement: Supplementary file 1 — (pdf 706 KB) [file 10618_2025_1140_MOESM1_ESM.pdf]

Supplementary material of “Detection and evaluation of clusters within sequential data”

Alexander Van Werde[1*], Albert Senen–Cerda[1,2], Gianluca Kosmella[1,3], Jaron Sanders[1]

[1*]Dept. of Mathematics & Computer Science, TU/e, The Netherlands.
[2]LAAS–CNRS, IRIT–CNRS, and Université de Toulouse, France.
[3]Dept. of Electrical Engineering, TU/e, The Netherlands.

This supplemententary material provides supporting information, such as algorithmic descriptions, experimental details, and certain proofs, for [1].

Section 1 provides pseudocode for the clustering algorithm of [2] that we implemented. Section 2 describes perturbed Block Markov Chain (BMC) models and provides details regarding the simulation experiment which was described in [1, Section 3.3]. The concentration inequality which was used to construct the confidence intervals in [1, Fig. 9], is described in Section 4. Section 5 proves [1, Proposition 1]. Tools which were used in the experiments and preprocessing are described in Section 7. Finally, some raw data and extra material describing our findings is provided in Section 8.

1 Pseudo-code describing the clustering procedure

The following pseudocode summarizes the clustering procedure that we have implemented. This pseudocode appeared first in [2], and we repeat it here for clarity and your convenience.

Algorithm 1 Spectral clustering algorithm, courtesy of [2].

Input: n, K and $\hat{N}$
Output: New cluster assignment $\hat{\mathcal{V}}'_1, \dots, \hat{\mathcal{V}}'_K$

$\hat{N}_\Gamma \leftarrow \text{Trim}(\hat{N})$
$\hat{R} \leftarrow K$-rank approximation of $\hat{N}_\Gamma$
$\hat{\mathcal{V}}_1, \dots, \hat{\mathcal{V}}_K \leftarrow K\text{-means}([\hat{R}, \hat{R}^T])$

The spectral clustering in Algorithm 1 is used to obtain a good initial estimate for the clusters. A k-means algorithm is used along a K-rank approximation of $\hat{N}$ (or $\hat{N}_\Gamma$ for the trimmed version of $\hat{N}$) to yield an initial guess for the clusters. It can be proved, however, that this step yields a number of misclassified states that is sublinear in n but not of constant order [3]. A second step is then required to attain exact recovery. In Algorithm 2, we see that a procedure similar to a likelihood ratio maximization is used to improve the cluster assignment. With this extra step it can be proven that the misclassified states will be order constant in expectation.

Algorithm 2 Cluster improvement algorithm (for 1st-order BMCs), courtesy of [2]

Input: n, K, ℓ, $\hat{N}$ and initial cluster assignment guess $\hat{\mathcal{V}}_1, \dots, \hat{\mathcal{V}}_K$.
Output: New cluster assignment $\hat{\mathcal{V}}'_1, \dots, \hat{\mathcal{V}}'_K$

for $a \leftarrow 1$ to K **do**
  $\hat{\pi}_a \leftarrow \hat{N}_{\hat{\mathcal{V}}_a,[n]}/\ell$, $\hat{\alpha}_a \leftarrow \#\hat{\mathcal{V}}_a/n$
  $\hat{\mathcal{V}}'_a \leftarrow \emptyset$
  for $b \leftarrow 1$ to K **do**
    $\hat{p}_{a,b} \leftarrow \hat{N}_{\hat{\mathcal{V}}_a,\hat{\mathcal{V}}_b}/\hat{N}_{\hat{\mathcal{V}}_a,[n]}$
  end for
end for
for $x \leftarrow 1$ to n **do**
  $c \leftarrow \text{argmax}_{l\in[K]} \sum_{k=1}^{K} \left(\hat{N}_{x,\hat{\mathcal{V}}_k} \ln(\hat{p}_{l,k}) + \hat{N}_{\hat{\mathcal{V}}_k,x} \ln(\hat{p}_{k,l}/\hat{\alpha}_l)\right) - \frac{\ell}{n}\frac{\hat{\pi}_l}{\hat{\alpha}_l}$
  $\hat{\mathcal{V}}'_c \leftarrow \hat{\mathcal{V}}'_c \cup \{x\}$
end for

2 Robustness of the clustering procedure to model violations

Recall that the asymptotic consistency of the clustering procedure has been theoretically studied in [3] under the assumption that the data-generating process is a BMC. In this section we aim to study the robustness of the clustering procedure to violations of this model assumption. That is, we investigate the performance of the clustering procedure when the data-generating process is not actually a BMC. We study two main measures of performance. First, in Section 2.1, we consider the number of misclassified states. Second, in Section 2.2, we consider the approximation error in a parameter estimation problem where the objective is to estimate the true transition matrix P of a Markovian data-generating process which need-not be a BMC.

The first measure of performance requires that the notion of misclassification is sensible even though the data-generating process is not a BMC. To this end we restrict ourselves to models where communities are still well-defined. More precisely, we consider the perturbed BMC model which was defined in [1, Section 3.2] and assign as ground-truth communities those of the BMC-kernel which was used to construct the perturbed model. Recall that the definition of a perturbed BMC requires to specify the nature of the perturbation kernel Δ. The following kernels are used for this purpose to model different types of model violations:

(i) *Uniform Stochastic*: The matrix Δ is sampled uniformly at random in the set of stochastic matrices. This is accomplished by sampling each row independently from a
Dirichlet$(1/n, \ldots, 1/n)$ distribution.

(ii) *Degree* 0: Fix some $\pi_1, \ldots, \pi_n > 0$ with $\sum_{i=1}^n \pi_i = 1$ and let $\Delta_{ij} = \pi_j$ for all $i, j \in [n]$. We construct the π_i by sampling independent exponential random variables $e_1, \ldots, e_n \sim \text{Exponential}(1)$ and normalizing $\pi_i = e_i / (\sum_{j=1}^n e_j)$.

(iii) *Heavy Tailed*: Let X be a random matrix whose entries X_{ij} are i.i.d. positive random variables with a heavy-tailed distribution. The kernel Δ is then found by normalizing the rows in order to achieve a stochastic matrix $\Delta := \text{diag}\big((\sum_j X_{ij})^{-1}\big)_{i=1}^n X$. We sample the heavy-tailed entries X_{ij} from a Zipf distribution with exponent $s = 3/2$.

(iv) *Sparse*: Consider constants $d > 0$ and $c > 0$ and construct a random matrix $X = A + cJ$ where A is the adjacency matrix from a directed Erdös–Rényi random graph with average outgoing degree d and J is a constant matrix $J_{ij} = 1/n$. The kernel Δ is then found by rescaling the rows in order to achieve a stochastic matrix $\Delta = \text{diag}\big((\sum_j X_{ij})^{-1}\big)_{i=1}^n X$. We take $d = 5$ and $c = 0.1$.

In our subsequent experimentation we take $n = 2m$ to be an even integer. The BMC which is perturbed is chosen to have two equally-sized clusters ($K = 2$) and cluster transition matrix given by

$$p = \begin{pmatrix} 0.6 & 0.4 \\ 0.4 & 0.6 \end{pmatrix}.$$

2.1 Misclassification ratio for perturbed BMCs

This section concerns the number of misclassified states when clustering on a perturbed BMC model. Recall that we chose the BMC model to have two equally-sized clusters which means that we may pick the cluster assignment map to be given by $\sigma_n(i) = 1 + \mathbb{1}[i > n/2]$. Let $\hat{\sigma_n} : [n] \to \{1, 2\}$ be an estimated cluster assignment which is output by the clustering procedure. Then, the misclassification ratio $\mathcal{E}$ is defined as

$$\mathcal{E} := \frac{1}{n} \min_{\rho \in S_2} \#\{v \in [n] : \sigma_n(v) \neq (\rho \circ \hat{\sigma_n})(v)\}. \tag{1}$$

Here S_2 denotes the set of permutations of $\{1, 2\}$.

Recall from [1, Equation 9] that the parameter ε of the perturbed BMC measures the fraction of transitions which are affected by the perturbation. In other words, ε measures the strength of the perturbation. The estimated expected misclassification ratio $\mathbb{E}[\mathcal{E}]$ is displayed as a function of the perturbation level ε for a numerical experiment in [1, Fig. 4(a)]. Up to $\varepsilon \approx 0.1$ the algorithm succeeds in recovering the exact cluster assignment for all four models. The exact number will naturally depend on the parameters of the BMC which was perturbed and will consequently be different in different contexts. At any rate, we conclude from this experiment that the algorithm appears to be robust with regards to small to medium-sized model violations.

The observation that some model violations can be tolerated may be understood theoretically in terms of the construction of the algorithm. This robustness is namely natural at the level of the spectral step of the algorithm. Consider that in a perturbed BMC one has the following decomposition:

$$\begin{aligned}\hat{N}_{\text{Perturbed}} &= \mathbb{E}[\hat{N}_{\text{BMC}}] + (\mathbb{E}[\hat{N}_{\text{Perturbed}}] - \mathbb{E}[\hat{N}_{\text{BMC}}]) + (\hat{N}_{\text{Perturbed}} - \mathbb{E}[\hat{N}_{\text{Perturbed}}]) \\ &=: \mathbb{E}[\hat{N}_{\text{BMC}}] + E_{\text{Perturbation}} + E_{\text{Noise}}.\end{aligned}$$

The sampling noise E_{Noise} is small in operator norm relative to $\mathbb{E}[\hat{N}_{\text{BMC}}]$ when the sample path is sufficiently long. It may further be expected that $E_{\text{Perturbation}}$ is small in operator norm whenever the perturbation level ε is small. Now recall that spectral step in the algorithm relies on singular value decomposition to compute a rank-K approximation. The purpose of this rank-K approximation, when the process is truly a BMC, is to separate the sampling noise E_{Noise} from the low-rank signal $\mathbb{E}[\hat{N}_{\text{BMC}}]$. In a small perturbation of a BMC the singular value decomposition will however also regard $E_{\text{Perturbation}}$ as an error term. Consequently, for small perturbations, the spectral step has the beneficial effect that it separates the perturbative error $E_{\text{Perturbation}}$ from the low-rank signal $\mathbb{E}[\hat{N}_{\text{BMC}}]$.

2.2 Bias–variance tradeoff for parameter estimation in a perturbed BMC

It may occur in some cases that one is not interested in the clusterings themselves but rather views them as a means to an end. Consider the scenario where one desires to estimate the transition kernel of a Markovian process which need not be a BMC.

Assume that one has prior reason to suspect that there could be some underlying clusters in the data but also that there could be parts of the dynamics which do not respect the clusters. In such a case a perturbed BMC would be a suitable model for the data. Let us emphasize that one is here not intrinsically interested in the BMC-component P_{BMC} but rather desires to estimate the ground-truth $P_{\text{True}} := (1-\varepsilon)P_{\text{BMC}} + \varepsilon\Delta$. It could however be the case that one can exploit the underlying clusters to improve the performance of estimation.

Assume that one knows the number of underlying clusters K and has access to a sample path $X_0^\varepsilon, \dots, X_\ell^\varepsilon$ of length ℓ of a perturbed BMC. Let $\hat{N}$ also denote the associated empirical frequency matrix. A natural general-purpose estimator for the transition matrix, which does not rely on the existence of clusters, is given by the empirical transition matrix $\hat{P}(\ell)$. The entries of the empirical transition matrix are given by

$$\hat{P}_{\text{Empirical}}(\ell)_{ij} := \begin{cases} \frac{\hat{N}_{ij}}{\sum_{k=1}^{n} \hat{N}_{ik}}, & \text{if } \hat{N}_{ij} \neq 0 \\ 0, & \text{if } \hat{N}_{ij} = 0. \end{cases} \tag{2}$$

Another estimator may be found by first computing a clustering $\hat{\mathcal{V}}_1, \dots, \hat{\mathcal{V}}_K$. One can then hope that, since $P_{\text{True}} \approx P_{\text{BMC}}$ for $\varepsilon \approx 0$, it would be sufficient to consider an estimator $\hat{P}_{\text{BMC}}$ for P_{BMC} whose entries are given by

$$\hat{P}_{\text{BMC}}(\ell)_{ij} := \begin{cases} \frac{1}{\#\hat{\mathcal{V}}_{\hat{\sigma_n}(j)}} \frac{\sum_{x\in\hat{\mathcal{V}}_{\hat{\sigma_n}(i)}, y\in\hat{\mathcal{V}}_{\hat{\sigma_n}(j)}} \hat{N}_{x,y}}{\sum_{m=1}^{K}\sum_{x\in\hat{\mathcal{V}}_{\hat{\sigma_n}(i)}, y\in\hat{\mathcal{V}}_m} \hat{N}_{x,y}}, & \text{if } \sum_{x\in\hat{\mathcal{V}}_{\hat{\sigma_n}(i)}, y\in\hat{\mathcal{V}}_{\hat{\sigma_n}(j)}} \hat{N}_{x,y} \neq 0 \\ 0, & \text{if } \sum_{x\in\hat{\mathcal{V}}_{\hat{\sigma_n}(i)}, y\in\hat{\mathcal{V}}_{\hat{\sigma_n}(j)}} \hat{N}_{x,y} = 0. \end{cases} \tag{3}$$

Finally, for comparison we also consider the following trivial estimator which does not even use the data

$$\hat{P}_{\text{Uniform}}(\ell)_{ij} = \frac{1}{n}.$$

We measure the performance of these estimators as a function of the length of the sample path using the expected estimation error:

$$R_*(\ell) := \mathbb{E}[\|P_{\text{True}} - \hat{P}_*(\ell)\|] \quad \text{where } * \in \{\text{Empirical}, \text{BMC}, \text{Uniform}\}. \tag{4}$$

Here, $\|\cdot\|$ denotes the operator norm $\|M\| = \sup_{\|v\|_2=1} \|Mv\|_2$.

We conduct a numerical experiment with a state space of size $n = 1000$ and a heavy-tailed perturbation model of perturbation strength $\varepsilon = 0.05$. Estimated values of the expected estimation error $R_*(\cdot)$ as a function of the length ℓ of the sample path are displayed in [1, Fig. 4(b)]. A number of different regimes may be identified. First, the regime where the sample path is very short meaning that $\ell \approx 10^4$. Here the empirical estimator $\hat{P}_{\text{Empirical}}$ and the BMC estimator $\hat{P}_{\text{BMC}}$ are both unable to outperform the trivial estimator $\hat{P}_{\text{Uniform}}$. The empirical estimator even performs significantly worse than the trivial estimator in this regime. Second, the regime where sample path is medium-sized meaning that $\ell \approx 10^5$. Here the clustering procedure succeeds and $\hat{P}_{\text{BMC}}$ becomes the best-performing estimator. Finally, the regime where the sample path grows long meaning that $\ell > 10^6$. Here the empirical estimator becomes

the best-performing estimator. These different regimes can be understood in terms of a bias–variance tradeoff. Namely, consider that for short to medium-sized sample paths the BMC estimator $\hat{P}_{\text{BMC}}$ has significantly less variance than the empirical estimator $\hat{P}_{\text{Empirical}}$ due to depending on fewer parameters. This decreased variance is the dominant consideration for the approximation error in this regime. On the other hand, for long sample paths both estimators $\hat{P}_{\text{BCM}}$ and $\hat{P}$ have low variance and the bias incurred by the approximation $P_{\text{True}} \approx P_{\text{BMC}}$ becomes dominant.

3 Methods for evaluating clusters and models

We next discuss methods which can aid in evaluating clusters and models for sequential data obtained from real-world processes. These methods have to account for the fact that, since we are dealing with real-world nonsynthetic data, we do not know the true process which generated the data. In particular, we do not have access to a ground-truth clustering.

3.1 Performance on a downstream task

One reason to cluster observations of sequential data, is that the clusters provide a tool for dimensionality reduction in subsequent statistical analyzes or optimization procedures. For instance, the running time of a numerical method which aims to execute some computational task on a sequence of observations may grow considerably with the number of distinct observations n. In such a case it is clear that one has to reduce n or otherwise use a different algorithm. Reducing n can also help to reduce overfitting, and aid in interpretability.

On the other hand, clustering naturally removes some information from the dataset. Thus, in a good clustering, the data should retain as much useful information as is possible. The meaning of "amount of useful information" is here ambiguous and depends on the context. There are cases, however, where the notion can be made concrete. For instance, suppose that one has a measure of quality $Q_{\text{pre-reduction}} := Q(T)$, evaluating performance of a downstream task $T := T(X_{1:\ell})$ applied to the sequence of observations. For example, if the algorithm is estimating parameters of some parametric model, then $Q_{\text{pre-reduction}}$ may be the accuracy of prediction on a validation dataset. One can now use this measure of quality $Q_{\text{pre-reduction}}$ as a proxy for the notion of useful information in a clustering. Given a clustering $\sigma_n : [n] \to [K]$ that reduces the number of distinct observations to some $1 \leq K \ll n$, one can apply the numerical solution method to obtain a solution $\tilde{T} := T(\sigma_n(X_{1:\ell}))$. The quantity $Q_{\text{reduced}} := Q(\tilde{T})$ then allows us to determine the quality of the clusters.

Using Q to determine the amount of useful information in clusters can help compare the quality of a number of different clusters which are output by different clustering algorithms. It can also happen that $Q_{\text{reduced}} > Q_{\text{pre-reduction}}$ due to the reduction of noise within the sequence of grouped observations. This effect may occur regardless of whether the task is numerically challenging. When the task is numerically challenging, then the dimension reduction (from n to K) by the map σ_n means that we can expect improved performance over methods that do not cluster data when fixing the computational budget.

In the following Sections 3.2 to 3.4 we discuss methods which can also reveal whether the BMC model is appropriate, and do not require some data-specific measure of quality.

3.2 Model selection with validation data

Section 3.1 mentioned that prediction of validation data can serve as a measure of quality Q. We now expand on this idea.

3.2.1 Rescaled log-likelihood ratio

Assume we observe sequential data $X_{1:\ell}$ generated by some ground-truth probability distribution $\mathbb{T}$ on $[n]^{\ell+1}$. The law $\mathbb{T}$ can in principle be arbitrarily complex; for example, the Markov property need not be satisfied. Note that, for nonsynthetic data, we typically do not have access to the ground-truth $\mathbb{T}$. Suppose however that we do have two candidate models $\mathbb{P}$ and $\mathbb{Q}$ which are also defined on $[n]^{\ell+1}$. We then want to determine whether $\mathbb{P}$ or $\mathbb{Q}$ is a better model based on the observed sequential data $X_{1:\ell}$.

For this purpose, we consider a log-likelihood ratio. Namely, given $x_{1:\ell} \in [n]^{\ell+1}$, consider the quantity

$$\hat{D}(x_{1:\ell}; \mathbb{P}, \mathbb{Q}) := \frac{1}{\ell} \ln \frac{\mathbb{P}[X_{1,\ell} = x_{1:\ell}]}{\mathbb{Q}[X_{1:\ell} = x_{1:\ell}]} \tag{5}$$

and its expectation

$$D(\mathbb{T}; \mathbb{P}, \mathbb{Q}) := \mathbb{E}_{\mathbb{T}}[\hat{D}(X_{1:\ell}; \mathbb{P}, \mathbb{Q})]. \tag{6}$$

Then, if $D(\mathbb{T}; \mathbb{P}, \mathbb{Q}) > 0$ we consider $\mathbb{P}$ to be a better approximation of the ground truth $\mathbb{T}$ and if $D(\mathbb{T}; \mathbb{P}, \mathbb{Q}) < 0$ we consider $\mathbb{Q}$ to be a better approximation. In practice we can not compute the expectation $\mathbb{E}_{\mathbb{T}}$ and instead consider the sign of the empirical estimator $\hat{D}(X_{1:\ell}; \mathbb{P}, \mathbb{Q})$.

In our experiments it is often the case that $\mathbb{P}$ and $\mathbb{Q}$ are Markov Chains (MCs) on $[n]$ whose transition matrices $P, Q \in [0,1]^{n \times n}$ are known. In this case one can alternatively express (5) as

$$\hat{D}(x_{1:\ell}; \mathbb{P}, \mathbb{Q}) = \frac{1}{\ell} \sum_{t=1}^{\ell-1} \ln \frac{P_{x_t, x_{t+1}}}{Q_{x_t, x_{t+1}}}. \tag{7}$$

Confidence bounds for the estimation of $D(\mathbb{T}; \mathbb{P}, \mathbb{Q})$ by $\hat{D}(X_{1:\ell}; \mathbb{P}, \mathbb{Q})$ in this MC-setting are provided in Section 4. It is there additionally assumed that $\mathbb{T}$ is a MC, possibly time-inhomogeneous, whose mixing time is known.

3.2.2 Information-theoretic interpretation for $D(\mathbb{T}; \mathbb{P}, \mathbb{Q})$

Let us briefly note that (6) has an information-theoretic interpretation. Namely, observe that

$$D(\mathbb{T}; \mathbb{P}, \mathbb{Q}) = \frac{1}{\ell}(\mathrm{KL}(\mathbb{T}; \mathbb{Q}) - \mathrm{KL}(\mathbb{T}; \mathbb{P})) \tag{8}$$

where KL denotes the Kullback–Leibler divergence

$$\mathrm{KL}(\mathbb{T};\mathbb{P}) := \mathbb{E}_{Z_{1:\ell}\sim\mathbb{T}}\Big[\ln\Big(\frac{\mathbb{T}(X_{1:\ell}=Z_{1:\ell})}{\mathbb{P}(X_{1:\ell}=Z_{1:\ell})}\Big)\Big]. \tag{9}$$

One can interpret the quantity $\mathrm{KL}(\mathbb{T};\mathbb{P})$ as the expected amount of discriminatory information revealing that $\mathbb{P}$ is not quite the ground-truth probability distribution underlying the sample path $X_{1:\ell}$; see [4]. In many cases, such as when the ground-truth $\mathbb{T}$ is an ergodic Markov chain, it further holds that (9) grows linearly in terms of amount of data ℓ.

Correspondingly, by (8), one can view $D(\mathbb{T};\mathbb{P},\mathbb{Q})$ as measuring the rate of growth for discriminatory information revealing that $\mathbb{P}$ is a better approximation for the ground truth $\mathbb{T}$ than $\mathbb{Q}$. To emphasize this perspective we may refer to $\hat{D}(X_{1:\ell};\mathbb{P},\mathbb{Q})$ as the *Kullback–Leibler divergence rate difference estimator*.

3.2.3 Estimation when the models are inferred from the data

Our experiments routinely determine two different candidate models that we wish to compare, from the same one sample sequence available to us. Let us emphasize this fact by referring to these candidate models as

$$\hat{\mathbb{P}}^{X_{1:\ell}} \quad \text{and} \quad \hat{\mathbb{Q}}^{X_{1:\ell}}. \tag{10}$$

Observe now that these two candidate models are a function of the observed data $X_{1:\ell}$. Substituting (10) into (5) could consequently result in a biased estimator and typically favor models with many parameters; the "optimal" model would be the degenerate probability distribution assigning probability 1 to the observed $X_{1:\ell}$.

To reduce the bias, we use a holdout method. Specifically, we will split the trajectory into two parts: the first half $X_{1:\lfloor \ell/2\rfloor}$ will be used for training, and the second half $X_{\lfloor \ell/2\rfloor+1:\ell}$ for validation. The estimator

$$\hat{D}(X_{\lfloor \ell/2\rfloor+1:\ell};\hat{\mathbb{P}}^{X_{1:\lfloor \ell/2\rfloor}},\hat{\mathbb{Q}}^{X_{1:\lfloor \ell/2\rfloor}}) \tag{11}$$

will then significantly reduce the amount of bias when compared to the estimator obtained by substituting (10) into (5).

Note that (11) can be viewed as a measure of quality in the language of Section 3.1. Eq. (11) namely compares whether $\hat{\mathbb{P}}^{X_{1:\lfloor \ell/2\rfloor}}$ or $\hat{\mathbb{Q}}^{X_{1:\lfloor \ell/2\rfloor}}$ better predicted the validation data.

3.3 Model selection with only training data

As discussed in Section 3.2, the Kullback–Leibler-divergence rates can provide a good rule–of–thumb for assessing what models are most interesting but are biased towards models with more parameters if one does not split the data into training and validation data. Splitting the data is however sometimes undesirable. Namely, if the data is sparse, the estimated models will become even less accurate. In order to overcome this

issue, we will use information criteria that compensate the bias incurred and use it to assess the order of the cluster process.

3.3.1 Problem setting: order of a BMC

Suppose that a sequence $X_{1:\ell}$ was in fact generated by some rth-order BMC, but that the order $r \in \{0, 1, \ldots\}$ is unknown. We will use techniques for model selection to try and determine r from the cluster sequence $Y_{1:\ell} = \sigma_n(X_{1:\ell})$.

There are two reasons for using $Y_{1:\ell}$ instead of $X_{1:\ell}$. First, the parametric models for higher order MCs without clusters have a comparable number of free parameters as the sequence length ℓ itself, so estimators for the order will behave poorly. If we look at the cluster chain instead, the number of degrees of freedom will depend on the cluster number K instead of the number of states n, and fortunately $K \ll n$. Secondly, we can also study the robustness of the model selection procedure depending on the clustering algorithm.

3.3.2 Order selection by minimizing an information criterion

The parameter that determines the rth-order BMC model for $Y_{1:\ell}$ is a transition matrix Q^r; recall [1, Equation 9]. Note here that the chain $Y^r_{1:\ell-r}$ will be constructed from the chain of clusters $Y_{1:\ell} = \sigma_n(X_{1:\ell})$ for a fixed cluster assignment σ_n.

To estimate Q^r one can consider the log-likelihood

$$\mathcal{L}(Y_{1:\ell} \mid Q^r) := \sum_{t=r}^{\ell-r-1} \ln Q^r_{Y_{t-r+1:t}, Y_{t+1}}. \tag{12}$$

The maximum-likelihood estimator associated with (12) is namely given by

$$(\hat{Q}^{r,\mathrm{MLE}})_{i^r,j} := \begin{cases} 0 & \text{if } \sum\limits_{t=r}^{\ell-r-1} \mathbb{1}[Y_{t-r+1:t} = i^r] = 0, \\ \dfrac{\sum\limits_{t=r}^{\ell-r-1} \mathbb{1}[Y_{t-r+1:t}=i^r, Y_{t+1}=j]}{\sum_{t=r}^{\ell-r-1} \mathbb{1}[Y_{t-r+1:t}=i^r]} & \text{otherwise.} \end{cases} \tag{13}$$

Here i^r, j run over all possible sequences in $[K]^r$ and $[K]$ respectively. We denote $\hat{\mathbb{Q}}^{r,\mathrm{MLE}}$ for the law of an rth-order MC with K states and transition matrix $\hat{Q}^{r,\mathrm{MLE}}$.

To determine what order r is the true underlying order of the data one would like to compare $\hat{\mathbb{Q}}^{r,\mathrm{MLE}}$ and $\hat{\mathbb{Q}}^{s,\mathrm{MLE}}$ for some $s \neq r$. As has been remarked in [1, Section 3.1.2.], using (5) for this purpose would give a biased estimator. Problems with bias in model selection are well-known in the statistics literature and to avoid this issue, the so-called *information criteria* were developed [5–8], where to a log-likelihood a penalty term is added to correct the bias.

In our setting we need a penalty term that is sensitive to sparse data and is also consistent. For this purpose, we have chosen the Consistent Akaike Information Criterion (CAIC) [6]: for model $\hat{\mathbb{Q}}^{r,\mathrm{MLE}}$,

$$\mathrm{CAIC}(\hat{Q}^{r,\mathrm{MLE}}) := -2\ln\big(\mathcal{L}(Y_{1:\ell} \mid \hat{Q}^{r,\mathrm{MLE}})\big) + 2\mathrm{DF}(K,r)\big(1 + \ln(\ell - r)\big). \tag{14}$$

Here, $\mathrm{DF}(K,r)$ the degrees of freedom in an rth-order MC constrained to have fixed parameters K and r. Specifically,

$$\mathrm{DF}(K,r) = K^r(K-1) \tag{15}$$

where the factor $(K-1)$ is due to the fact that the rows of Q^r are constrained to add up to one. We will utilize the CAIC to select the right order as follows. From the collection of models $\hat{\mathbb{Q}}^{0,\mathrm{MLE}}$, $\hat{\mathbb{Q}}^{1,\mathrm{MLE}}$, $\hat{\mathbb{Q}}^{2,\mathrm{MLE}}$, $\ldots$, we may determine the order r^{CAIC} that minimizes the CAIC:

$$r^{\mathrm{CAIC}} := \mathrm{argmin}_{r\in\{0,1,2,\ldots\}}\, \mathrm{CAIC}(\hat{Q}^{r,\mathrm{MLE}}). \tag{16}$$

Note that lower-dimensional models are favored since the degrees of freedom $\mathrm{DF}(K,r)$, and thus the penalty terms in (14), increase exponentially in K,r.

In order to evaluate how robust the CAIC criterion is, we will estimate the over- and underfit error probabilities with error models and draw conclusions on the selected orders.

3.4 The shape of spectral noise for identification of alternative models

The methods in Sections 3.1 to 3.2 allow us to compare a BMC to alternative models. The selection of a good alternative can however be difficult when a more complex model than a BMC is desirable. The method described here can aid in the selection of an alternative model.

The method is based on a result from [9] which describes the histogram of the singular values of $\hat{N}$ in the asymptotic regime $n \to \infty$ under the condition that $\ell = \Theta(n^2)$. The results in [10] can further be interpreted as the statement that the K nonzero singular values of $\mathbb{E}[\hat{N}]$ correspond to the K largest singular values of $\hat{N}$. In other words, all singular values except these leading few may be interpreted as being due to the noise $\hat{N} - \mathbb{E}[\hat{N}]$. The histogram of the nonleading singular values may thus be interpreted as the *shape of the spectral noise*.

These results and their interpretation can guide the selection of a good model. One can namely identify clusters in the data and visually compare the associated BMC-prediction with the observed histogram. If there is a good match, then this may indicate that a BMC suits the data well. If there is a discrepancy, then the nature of the discrepancy can be informative of the properties that the alternative model should have. It will for instance be shown in Section 5.4 that a long tail can sometimes be explained using a heavy-tailed perturbation.

We have, however, found that a strongly inhomogeneous equilibrium distribution in the data can dominate the spectral noise in $\hat{N}$. So long as the clustering respects the equilibrium distribution it then follows that the observations will indeed resemble the theory. This is an issue since it follows that, in the case of an inhomogeneous equilibrium distribution, the spectral noise of $\hat{N}$ may not be particularly informative. In such a case one can consider a different random matrix.

The *empirical normalized Laplacian* $\hat{L}$ associated to the observation sequence is element-wise given by

$$\hat{L}_{ij} := \begin{cases} \frac{\hat{N}_{ij}}{\sqrt{\sum_{k=1}^{n} \hat{N}_{ik}}\sqrt{\sum_{k=1}^{n} \hat{N}_{kj}}} & \text{if } \hat{N}_{ij} \neq 0, \\ 0 & \text{otherwise.} \end{cases} \tag{17}$$

We argue in Section 5.3 that the variance of the entries of $\hat{L}$ is approximately independent of the equilibrium distribution. Consequently, we expect that the spectral noise of $\hat{L}$ will not be dominated by a possibly inhomogeneous equilibrium distribution. A proposition describing the limiting histogram of singular values is proved in Section 5.2. The precise statement is technical but a summary may be found in Proposition 1.

Proposition 1. *Let $X_{1:\ell}$ be a sample path of a BMC. If $\ell = \Theta(n^2)$, then for almost every $a, b \in \mathbb{R}$ the fraction of singular values in $[a, b]$, i.e., $n^{-1}\#\{i : s_i(\sqrt{n}\hat{L}) \in [a, b]\}$ converges in probability as $n \to \infty$. The limit may be computed explicitly in terms of the parameters of the BMC.*

With Proposition 1, we can characterize the spectral noise of $\hat{L}$ in a BMC and use the spectrum as a tool for data exploration, expectedly even in the presence of an inhomogeneous equilibrium distribution.

4 Confidence bounds when estimating $D(\mathbb{T}; \mathbb{P}, \mathbb{Q})$

We here state a concentration inequality from which we deduce the confidence interval in (27). Recall that these confidence intervals are used in [1, Fig. 9]. The proof is based on a result from [11] whose assumptions we first verify.

Assume that the true process $\{X_t\}_{t\geq 0}$ generating the sequential data $X_1, \ldots, X_\ell$ is a MC, which need not be time-homogeneous. Let us refer to $\{X_t\}_{t\geq 0}$'s law as $\mathbb{T}$. The mixing time of $\{X_t\}_{t\geq 0}$ is defined as

$$\tau_{\text{mix}} := \min\{t \geq 1 : \overline{d}(t) \leq \tfrac{1}{2}\}, \tag{18}$$

where

$$\overline{d}(t) := \max_{1\leq i\leq \ell-t} \sup_{x,y\in[n]} \mathrm{d}_{\mathrm{TV}}\big(\mathbb{T}[X_{i+t} = \cdot \mid X_i = x], \mathbb{T}[X_{i+t} = \cdot \mid X_i = y]\big). \tag{19}$$

Here, d_{TV} denotes the *total variation distance*:

$$\mathrm{d}_{\mathrm{TV}}\big(\mathbb{T}[X_{i+t} = \cdot \mid X_i = x], \mathbb{T}[X_{i+t} = \cdot \mid X_i = y]\big) \tag{20}$$

$$:= \tfrac{1}{2} \sum_{z\in[n]} \big|\mathbb{T}[X_{i+t} = z \mid X_i = x] - \mathbb{T}[X_{i+t} = \cdot \mid X_i = y]\big|. \tag{21}$$

We claim that the MC of transitions $\{E_{X,t}\}_{t\geq 0}$, where $E_{X,t} := (X_t, X_{t+1})$, then has mixing time at most $\tau_{\text{mix}} + 1$. Indeed, observe that for any $t \geq \tau_{\text{mix}} + 1$, $x_1, x_2, y_1, y_2 \in$

$[n]$ and $1 \le i \le \ell - t - 1$,

$$
\begin{aligned}
&\tfrac{1}{2} \sum_{z_1, z_2 \in [n]} |\mathbb{P}[E_{X,i+t} = (z_1, z_2) \mid E_{X,i} = (x_1, x_2)] - \mathbb{P}[E_{X,i+t} = (z_1, z_2) \mid E_{X,i} = (y_1, y_2)]| \\
&= \tfrac{1}{2} \sum_{z_1, z_2 \in [n]} \mathbb{P}[X_{i+t+1} = z_2 \mid X_{i+t} = z_1] \qquad (22)\\
&\qquad\qquad \times \big|\mathbb{P}[X_{i+t} = z_1 \mid X_{i+1} = x_2] - \mathbb{P}[X_{i+t} = z_1 \mid X_{i+1} = y_2]\big| \\
&= \tfrac{1}{2} \sum_{z_1 \in [n]]} \big|\mathbb{P}[X_{i+t} = z_1 \mid X_{i+1} = x_2] - \mathbb{P}[X_{i+t} = z_1 \mid X_{i+1} = y_2]\big| \le \tfrac{1}{2}. \qquad (23)
\end{aligned}
$$

Here, the Markov property was used to conclude (22). The fact that $\mathbb{P}(X_{i+t=1} = \cdot \mid X_{i+t} = z_1)$ defines a probability distribution, together with the assumption that $t \ge \tau_{\text{mix}} + 1$ and the property that $\overline{d}(t)$ is nonincreasing in t, was used to arrive at (23).

Now suppose that we are given two MCs with fixed transition matrices P and Q, whose laws we will refer to as $\mathbb{P}$ and $\mathbb{Q}$, respectively. Assume furthermore that $\max_{i,j\in[n]} |\ln(P_{i,j}/Q_{i,j})| \le \delta$ for some $\delta > 0$. For any two sample paths $X_1, \dots, X_\ell$ and $Y_1, \dots, Y_\ell$, it then holds that

$$|\hat{D}(X_1, \dots, X_\ell; P, Q) - \hat{D}(Y_1, \dots, Y_\ell; P, Q)| \le \frac{2\delta}{\ell} \sum_{t=1}^{\ell-1} \mathbb{1}[E_{X,t} \ne E_{Y,t}]. \qquad (24)$$

Consequently, [11, Corollary 2.10] applied to the MC $\{E_{X,t}\}_{t\ge 0}$ yields the desired concentration inequality:

$$\mathbb{P}\big(|\hat{D}(X_0, \dots, X_\ell; \mathbb{P}, \mathbb{Q}) - D(\mathbb{T}; \mathbb{P}, \mathbb{Q})| > t\big) \le 2 \exp\Big(\frac{-t^2 \ell^2}{18\delta^2(\tau_{\text{mix}} + 1)}\Big). \qquad (25)$$

In conclusion: if we are given two MCs with fixed transition matrices P and Q for which $\max_{i,j\in[n]} |\ln P_{i,j}/Q_{i,j}| > 0$, together with an estimate for τ_{mix}, we can then construct for $z \in [0,1]$ a $100(1-z)\%$ confidence intervals of size

$$c_z := \frac{1}{\ell} \max_{i,j\in[n]} \Big|\ln \frac{P_{i,j}}{Q_{i,j}}\Big| \sqrt{18(\tau_{\text{mix}} + 1) \ln \frac{2}{z}}. \qquad (26)$$

This is to say that

$$\mathbb{P}\Big[D(\mathbb{T}; \mathbb{P}, \mathbb{Q}) \in \big[\hat{D}(X_0, \dots, X_\ell; \mathbb{P}, \mathbb{Q}) - c_z, \hat{D}(X_0, \dots, X_\ell; \mathbb{P}, \mathbb{Q}) + c_z\big]\Big] \ge 1 - z. \qquad (27)$$

5 Shape of the spectral noise

Recall that it was stated in [1, Section 7.4] that the spectral noise in $\hat{N}$ can be dominated by an inhomogeneous equilibrium distribution. It was further claimed that the Laplacian $\hat{L}$ does not suffer from this issue. The main goal in this section is to argue that this claim is true.

Some preliminary notation and concepts are introduced in Section 5.1 after which a theoretical result concerning the limiting singular value distribution of $\hat{L}$ is established in Section 5.2. A model with an inhomogeneous equilibrium distribution is introduced in Section 5.3. The claim that $\hat{L}$ can also detect violations to the model assumptions in the presence of an inhomogeneous equilibrium distribution is verified in Section 5.4 by a simulation experiment.

5.1 Preliminaries

The *empirical singular value distribution* ν_M of a matrix $M \in \mathbb{R}^{n\times n}$ with singular values $s_1(M) \geq \ldots \geq s_n(M)$ is the probability measure on $\mathbb{R}_{\geq 0}$ defined by

$$\nu_M(A) := \frac{1}{n}\#\{i \in [n] : s_i(M) \in A\} \tag{28}$$

for every measurable set $A \subseteq \mathbb{R}$. A sequence of random probability measures $\{\mu_n\}_{n\geq 1}$ on the real line is are said to *converge weakly in probability* to a probability measure μ if for every continuous bounded function $f : \mathbb{R} \to \mathbb{R}$ it holds that $\int f \mathrm{d}\mu_n$ converges weakly in probability to $\int f \mathrm{d}\mu$. The *symmetrization* of a probability measure μ on the positive real line $\mathbb{R}_{\geq 0}$ is the probability measure μ_{sym} on $\mathbb{R}$ given by

$$\mu_{\text{sym}}(A) := \tfrac{1}{2}\big(\mu(\{a : a \in A, a \geq 0\}) + \mu(\{-a : a \in A, a \leq 0\})\big) \tag{29}$$

for any measurable $A \subseteq \mathbb{R}$. Note that μ can be recovered from its symmetrization since for any measurable $A \subseteq \mathbb{R}_{\geq 0}$ it holds that

$$\mu(A) = 2\mu_{\text{sym}}(A \setminus \{0\}) + \mu_{\text{sym}}(\{0\}). \tag{30}$$

The *Stieltjes transform* of a probability measure μ is the analytic function $s : \mathbb{C}^+ \to \mathbb{C}^-$ given by $s(z) = \int 1/(z-x)\mathrm{d}\mu(x)$. Here, $\mathbb{C}^+ := \{z \in \mathbb{C} : \mathrm{Im}(z) > 0\}$ denotes the upper half-plane and $\mathbb{C}^- := \{z \in \mathbb{C} : \mathrm{Im}(z) < 0\}$ denotes the lower half-plane. The Stieltjes inversion formula [12, Theorem B.8] allows one to recover μ from its Stieltjes transform: for any continuity points $a < b$ of μ,

$$\mu([a,b]) = -\frac{1}{\pi}\lim_{\varepsilon\to 0^+}\int_a^b \mathrm{Im}(s(x+\sqrt{-1}\varepsilon))\mathrm{d}x. \tag{31}$$

5.2 Limiting law of singular value distribution of the Laplacian $\hat{L}$

Fix some positive integer $K \geq 1$ and a transition matrix $p \in \mathbb{R}^{K\times K}$ of an ergodic MC on $[K]$. Denote $\pi \in [0,1]^K$ for the equilibrium distribution of the MC associated to p. For every $n \geq 1$ consider a partition $\mathcal{V}_1 \cup \ldots \cup \mathcal{V}_K = [n]$ of the state space into K nonempty groups $\mathcal{V}_i$. The subsequent results are concerned with the asymptotic regime where $n \to \infty$. We here assume that there are $\alpha_1, \ldots, \alpha_K > 0$ such that $\#\mathcal{V}_i = \alpha_i n + o(n)$ and $\sum_{i=1}^K \alpha_i = 1$.

Proposition 2. *Let $\hat{L}$ be the empirical normalized Laplacian associated to a sample path $X_1, \ldots, X_\ell$ of the above BMC. Assume that as n tends to infinity it holds that $\ell = \lambda n^2 + o(n^2)$. Then, the empirical singular value distribution $\nu_{\sqrt{n}\hat{L}}$ converges weakly in probability to a compactly supported probability measure ν on $\mathbb{R}_{\geq 0}$. Moreover, the symmetrization ν_{sym} has Stieltjes transform $s(z) = \sum_{i=1}^{K} \alpha_i (a_i(z) + a_{K+i}(z))/2$ where $a_1, \ldots, a_{2K}$ are the unique analytic function from $\mathbb{C}^+$ to $\mathbb{C}^-$ such that the following system of equations is satisfied*

$$a_i(z)^{-1} = z - \sum_{j=1}^{K} \lambda^{-1} \pi(j)^{-1} \alpha_j p_{ij} a_{K+j}(z), \tag{32}$$

$$a_{i+K}(z)^{-1} = z - \sum_{j=1}^{K} \lambda^{-1} \pi(i)^{-1} \alpha_j p_{j,i} a_j(z) \tag{33}$$

for $i = 1, \ldots, K$.

The proof of Proposition 2 is similar to the proof of [13, Theorem 1.2] which is there given below [13, Proposition 4.7]. The intermediate [13, Lemma 4.4(ii)] should however be replaced by Lemma 3 below, and the role of [13, Equation (22)] is taken over by Lemma 4 below.

Lemma 3. *Let $\Pi_X \in [0,1]^n$ denote the equilibrium distribution of the BMC, and define*

$$\hat{Q} := \mathrm{diag}((\ell+1)\Pi_X)^{-1/2} (\hat{N} - \mathbb{E}[\hat{N}]) \,\mathrm{diag}((\ell+1)\Pi_X)^{-1/2}. \tag{34}$$

Assume that $\nu_{\sqrt{n}\hat{Q}}$ converges weakly in probability to some probability measure ν on $\mathbb{R}_{\geq 0}$. Under the assumptions of Proposition 2, it then holds that $\nu_{\sqrt{n}\hat{L}}$ converges weakly in probability to ν.

Proof. Consider the following notation:

$$\begin{aligned}
C_n &:= \mathrm{diag}((\ell+1)\Pi_X)^{-1/2} \mathbb{E}[\hat{N}] \,\mathrm{diag}((\ell+1)\Pi_X)^{-1/2}, \\
D_{n,l} &:= \mathrm{diag}\Big(\Big(\sum_{k=1}^{n} \hat{N}_{ik}\Big)_{i=1}^{n}\Big)^{-1/2} \mathrm{diag}((\ell+1)\Pi_X)^{1/2}, \\
D_{n,r} &:= \mathrm{diag}((\ell+1)\Pi_X)^{1/2} \,\mathrm{diag}\Big(\Big(\sum_{k=1}^{n} \hat{N}_{kj}\Big)_{j=1}^{n}\Big)^{-1/2}.
\end{aligned} \tag{35}$$

Observe that $\hat{L} = D_{n,l} \hat{Q} D_{n,r} + C_n$. Furthermore, $\max_{i=1}^{n} |(\ell+1)^{-1} \Pi_{X,i}^{-1} \sum_{k=1}^{n} \hat{N}_{ik} - 1|$ converges to zero in probability by [13, Corollary 6.11]. Since $x \mapsto 1/\sqrt{x}$ is continuous in the neighborhood of 1 and the operator norm of a diagonal matrix is the maximal value on its diagonal, it follows that $\|D_{n,l} - \mathrm{Id}\|_{\mathrm{op}}$ converges to zero in probability.

Note that transitions coming into state i are almost in bijection with the outgoing transitions out of state i. The only possible exceptions occur when $i = X_1$ or $i = X_\ell$.

This is to say that for every i

$$\Big|\sum_{k=1}^{n} \hat{N}_{ik} - \sum_{k=1}^{n} \hat{N}_{kj}\Big| \leq 2. \tag{36}$$

Hence, using that $(\ell+1)\Pi_{X,i} = \Theta(n)$ and the fact that we already know that $\max_{i=1}^{n} |(\ell+1)^{-1}\Pi_{X,i}^{-1} \sum_{k=1}^{n} \hat{N}_{ik} - 1|$ converges to zero in probability, it follows that $\max_{i=1}^{n} |(\ell+1)^{-1}\Pi_{X,i}^{-1} \sum_{k=1}^{n} \hat{N}_{ki} - 1|$ converges to zero in probability. By the continuity of $1/\sqrt{x}$ near 1 we may now also conclude that $\|D_{n,r} - \mathrm{Id}\,\|_{\mathrm{op}}$ converges to zero in probability.

By two applications of [13, Lemma 6.8.(iii)] we conclude that $\nu_{\sqrt{n}D_{n,l}\hat{Q}D_{n,r}}$ converges weakly in probability to ν.

Further, by the fact that the BMC starts in equilibrium it holds that $\mathrm{rank}(\mathbb{E}[\hat{N}]) \leq K$. Hence, using the general fact that $\mathrm{rank}(AB) \leq \mathrm{rank}(A)$ for any two matrices A, B of compatible size, we find that

$$\mathrm{rank}(\sqrt{n}C_n) \leq \mathrm{rank}(\mathbb{E}[\hat{N}]) \leq K. \tag{37}$$

An application of [13, Lemma 6.8.(ii)] now yields the desired result, since $\nu_{\sqrt{n}\hat{L}} = \nu_{\sqrt{n}(D_{n,l}\hat{Q}D_{n,r}+C_n)}$. ☐

Lemma 4. *Under the assumptions of Proposition 2 and with notation as in Lemma 3 it holds that as n tends to infinity*

$$\max_{ij=1,\dots,n} \left|\mathrm{Var}[\hat{Q}_{ij}] - \lambda^{-1}\pi(\sigma_n(j))^{-1} p_{\sigma_n(i)\sigma_n(j)}\right| = o(1). \tag{38}$$

Proof. This is immediate from [13, Corollary 4.6] using the fact that $\mathrm{Var}[cX] = c^2\,\mathrm{Var}[X]$ for any real random variable X and scalar $c \in \mathbb{R}$. ☐

5.3 Inhomogeneous equilibrium distribution: Degree-corrected Block Markov Chain (DC-BMC)

In order to allow for an inhomogeneous equilibrium distribution we consider the following model which is inspired by the analogous degree-corrected stochastic block model for communities in graphs with inhomogeneous degrees. Let $K \geq 1$ be a positive integer, consider a transition matrix $p \in \mathbb{R}^{K\times K}$ for an ergodic MC on $[K]$ and equip the state-space with a group-assignment map $\sigma_n : [n] \to [K]$. As was the case for BMCs we define the groups $\mathcal{V}_1, \dots, \mathcal{V}_K$ by $\mathcal{V}_i = \{v \in [n] : \sigma_n(v) = i\}$. Assume moreover that every group $\mathcal{V}_i$ is equipped with a probability distribution $\mu_i : \mathcal{V}_i \to [0,1]$. Then, a MC X_t on $[n]$ is called a DC-BMC if

$$\mathbb{P}(X_{t+1} = j \mid X_t = i) = p_{\sigma_n(i)\sigma_n(j)}\mu_{\sigma_n(j)}(j). \tag{39}$$

Recall that in a BMC it holds that conditional on $\sigma_n(X_t) = k$ for $t > 1$ the observation X_t is chosen uniformly at random in the cluster $\mathcal{V}_k$. In a DC-BMC it instead holds that

conditional on $\sigma_n(X_t) = k$ the observation X_t is chosen from the cluster $\mathcal{V}_k$ according to the probability measure μ_i.

Note that the usual BMC is recovered when all μ_i are taken to be the uniform measures on their respective groups $\mathcal{V}_i$. Furthermore, by taking a larger number of groups $\widetilde{K} = MK$ one can still approximate a DC-BMC model by a BMC-model. This is to say that one can use the additional clusters to separate each true group $\mathcal{V}_i$ of the DC-BMC model into M subgroups $\widetilde{\mathcal{V}}_{i,1}, \cdots, \widetilde{\mathcal{V}}_{i,M}$ such that μ_i is approximately constant on every $\widetilde{\mathcal{V}}_{i,j}$.

We expect that the limiting measure for $\nu_{\sqrt{n}\hat{L}}$ in a DC-BMC is equal to the limiting measure of a BMC with the same cluster transition matrix p and the same cluster ratios α_i provided that $\max_{i=1,\ldots,n} \mu_{\sigma_n(i)}(i) = \Theta(1/n)$ and $\min_{i=1,\ldots,n} \mu_{\sigma_n(i)}(i) = \Theta(1/n)$. If this conjecture is true then the limiting measure does not depend at all on the μ_i since these do not occur in Proposition 2. The insensitivity to the μ_i allows to ensure that the spectral noise in $\hat{L}$ is not dominated by an inhomogeneous equilibrium distribution. The main reason for this conjecture is that the proof of Proposition 2 implicitly relies on a universality principle of [13] which states that the limiting singular value distribution in a (sufficiently well-behaved) random matrix only depends on the variance of its entries. We will namely subsequently argue that the variance profile of $\hat{L}$ is approximately independent of distributions μ_k; see (46).

Denote π for the cluster equilibrium distribution of the Markov chain associated to p and note that the state equilibrium distribution of a DC-BMC is then given by $\Pi_{X,i} = \pi(\sigma_n(i))\mu_{\sigma_n(i)}$. Correspondingly, up to approximation errors on the order of $\sqrt{\ell}$,

$$\sum_{k=1}^{n} \hat{N}_{i,k} \approx \#\{t = 1, \ldots, \ell : X_t = i\} \approx \ell \Pi_{X,i} = \ell\pi(\sigma_n(i))\mu_{\sigma_n(i)}. \tag{40}$$

Therefore, by the continuity of $x \mapsto \sqrt{x}$ it may be expected that

$$\sqrt{\sum_{k=1}^{n} \hat{N}_{ik}} \approx \sqrt{\ell\pi(\sigma_n(i))\mu_{\sigma_n(i)}(i)} \qquad \text{and} \qquad \sqrt{\sum_{k=1}^{n} \hat{N}_{kj}} \approx \sqrt{\ell\pi(\sigma_n(j))\mu_{\sigma_n(j)}(j)}. \tag{41}$$

The variance of a sum of independent random variables is equal to the sum of the variances. If we write $\hat{N}_{i,j} = \sum_{t=1}^{\ell-1} \mathbb{1}[X_t = i, X_{t+1} = j]$ then these summands are not independent but nonetheless we do expect the variance to approximately distribute over the sum. Therefore, it is expected that

$$\operatorname{Var}[\hat{N}_{i,j}] \approx (\ell - 1) \operatorname{Var}[\mathbb{1}[X_t = i, X_{t+1} = j]] = (\ell - 1)\pi(\sigma_n(i))\mu_{\sigma_n(i)}(i)p_{\sigma_n(i)\sigma_n(j)}\mu_{\sigma_n(j)}(j). \tag{42}$$

By combining (41) and (42) it follows that

$$\operatorname{Var}[\hat{L}_{ij}] \approx \operatorname{Var}\left[\frac{\hat{N}_{ij}}{\sqrt{\ell\pi(\sigma_n(i))\mu_{\sigma_n(i)}(i)}\sqrt{\ell\pi(\sigma_n(j))\mu_{\sigma_n(j)}(j)}}\right] \tag{43}$$

$$\approx \frac{\ell\pi(\sigma_n(i))p_{\sigma_n(i)\sigma_n(j)}\mu_{\sigma_n(i)}(i)\mu_{\sigma_n(j)}(j)}{(\ell\pi(\sigma_n(i))\mu_{\sigma_n(i)}(i))(\ell\pi(\sigma_n(j))\mu_{\sigma_n(j)}(j))} \tag{44}$$

$$= \ell^{-1}\pi(\sigma_n(j))^{-1}p_{\sigma_n(i)\sigma_n(j)} \tag{45}$$

$$\approx (\lambda n^2)^{-1}\pi(\sigma_n(j))^{-1}p_{\sigma_n(i)\sigma_n(j)} \tag{46}$$

Observe that this agrees with the variance profile which was used in Lemma 4.

5.4 Simulation experiment

We here measure the sensitivity of the spectral noise in $\hat{L}$ and $\hat{N}$ to violations of the model assumptions in the presence of an inhomogeneous equilibrium distribution by means of a perturbation to a DC-BMC model, defined in Section 5.3. The experiment is done by means of a simulation.

For the DC-BMC model we take $K = 2$ and we consider clusters of size $\#\mathcal{V}_1 = \#\mathcal{V}_2 = 1000$. The cluster transition matrix p is defined by $p_{11} = p_{22} = 0.8$ and $p_{12} = p_{21} = 0.2$. The probability measures μ_i are found for $i = 1, 2$ by sampling a vector of i.i.d. exponentially distributed random variables of rate 1 and normalizing this vector to have L^1-norm equal to 1.

We may further consider a perturbation of this DC-BMC. Let Δ be a heavy-tailed transition matrix as defined in [1, Section 3.2] and denote $P_{\text{perturbed}} := 0.95P_{\text{DC-BMC}} + 0.05\Delta$. Recall that the DC-BMC component $P_{\text{DC-BMC}}$ can be approximated with a BMC with more groups but note that such an approximation is not possible for Δ. Consequently, we may think of the decomposition for $P_{\text{perturbed}}$ as splitting the ground truth model into a main part which can be approximated with a BMC and a second part which requires a different explanation.

In the subsequent experiment we consider observation sequences $\{X_t\}_{t=1,\ldots,\ell}$ and $\{Y_t\}_{t=0,\ldots,\ell}$ with length $\ell = 2000^2$ from the DC-BMC-model and the perturbed model respectively. The singular value densities of the $\hat{N}$-matrix constructed from X and Y are displayed in Fig. 1 (a). Also displayed in Fig. 1 is the theoretical prediction corresponding to a BMC found by executing the clustering algorithm with $\widetilde{K} = 4$ clusters. Recall that taking $\widetilde{K} > K$ allows for the algorithm to split the groups to ensure that μ_i is roughly constant. We observe that the empirical densities associated to the DC-BMC-model and the perturbed model look quite similar apart from the fact that the perturbed model has a longer tail. The theoretical prediction associated to the BMC further provides an acceptable match for the DC-BMC model but there is also some small part of the tail of the DC-BMC model which escapes the theoretical prediction. Here the issue regarding the sensitivity of $\hat{N}$ becomes apparent: there are at least two plausible explanations why in empirical data some part of the tail may escape the support of the theoretical density. A first explanation is the presence of a perturbation Δ which we view as a violation of the model assumptions. A second

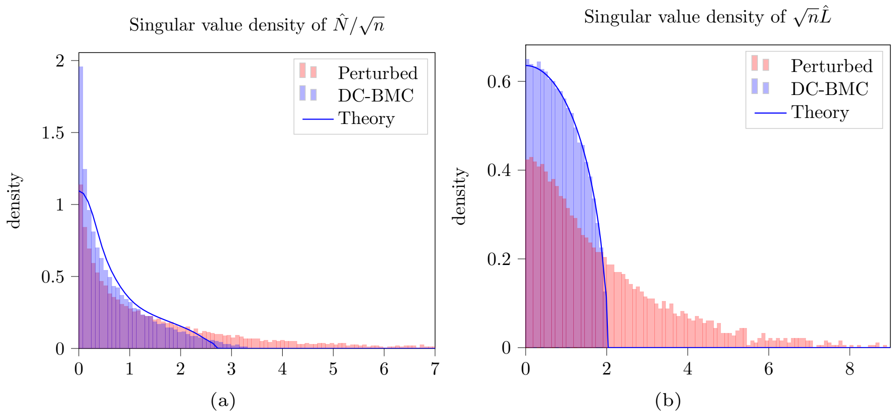

Fig. 1 (left) The singular value density of $\hat{N}$ for a simulated DC-BMC (blue bars) as compared to the theory (blue line) and a perturbed model (red bars). (right) The singular value density of $\hat{L}$.

explanation is that the ground truth is a DC-BMC and one should take $\widetilde{K}$ to be larger. These two explanations are difficult to distinguish from the spectral noise in $\hat{N}$. In the current example one may argue that the amount of the tail which escapes the theoretical density is larger in the perturbed model. Such a judgement regarding the size of the tail is however undesirable since it is vague and subjective.

The singular value density of $\hat{L}$ for the two sample paths $X_{1:\ell}$ and $Y_{1:\ell}$ is displayed in Fig. 1 (b). Here we observe that the empirical densities of the DC-BMC model and the perturbed model are severely different. The theoretical prediction associated to the BMC moreover provides a good match to the DC-BMC model as was expected by the conjecture of Section 5.3. We conclude that the spectral noise in the Laplacian $\hat{L}$ is more sensitive to violations of the model assumptions than the spectral noise in $\hat{N}$, particularly in the presence of an inhomogeneous equilibrium distribution.

6 Evaluation of the CAIC criterion

In [1], the CAIC is used to yield estimators for the order of the data-generating process for the DNA, Animal movement and Stock market datasets, respectively. In order to probe how accurate these estimators are, we consider a generative model for data with similar empirical transition laws as those of the datasets. Under several perturbation levels, we study both the over- and underfit probabilities of the order, and inspect how robust the estimator is under model perturbation.

We consider the empirical transition law $\hat{\mathbb{P}}^{r,\mathrm{MLE}}$ from the original data $X_{1:\ell}$ on the full state space $[n]$. With $\hat{\mathbb{P}}^{r,\mathrm{MLE}}$ for $r \in \{0,1\}$ we consider two perturbed data-generating models and investigate the clustered process Y_ε^r. The models are:

$\mathbb{W}_\varepsilon^1$: A perturbed 1st-order BMC with probability distribution $\hat{\mathbb{P}}^{1,\mathrm{MLE}}$ and a heavy-tailed 0th-order perturbation. In contrast to the general perturbed models described in [1, Section 3], the perturbation here is a 0th-order MC.

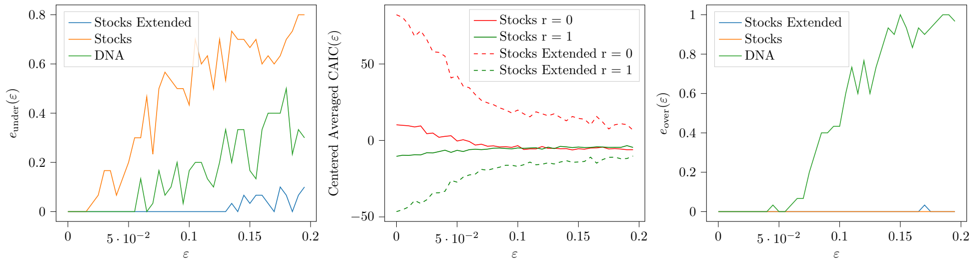

(a) (b) (c)

Fig. 2 (a) Underfit probability $e_{\text{under}}(\varepsilon)$ as function of ε for the DNA, S&P500, and extended S&P500 datasets assuming that the data-generating process is $\mathbb{W}^1_\varepsilon$. (b) Centered average of CAIC for datasets assuming the data-generating process is $\mathbb{W}^1_\varepsilon$. We remark that the empirical variance is an order of magnitude too large to be represented in the plot ($\text{Var}(\text{CAIC}(Y_{1:\ell}(\varepsilon)) \simeq O(10^2)$). Despite this very large variance, the selection process is robust for small error ε. (c) Overfit error probability $e_{\text{over}}(\varepsilon)$ as function of ε assuming the data-generating process is $\mathbb{W}^0_\varepsilon$. In all tests the number of repetitions was $R = 30$.

$\mathbb{W}^0_\varepsilon$: A perturbed 0th-order BMC with probability distribution $\hat{\mathbb{P}}^{0,\text{MLE}}$ and a heavy-tailed 1st-order perturbation.

Denote $Y^{r,\varepsilon}_{1:\ell} = \sigma_n(X^\varepsilon_{1:\ell})$ for the cluster process with $X^\varepsilon_{1:\ell} \sim \mathbb{W}^r_\varepsilon$ for $r \in \{0,1\}$. We will study the robustness of the CAIC criterion by examining how often it over- and underfits when selecting $s \in \{0,1\}$ for the models $\hat{\mathbb{Q}}^{s,\text{MLE}}$ with the clustered sequence $Y^{r,\varepsilon}_{1:\ell}$. The overfit error probability is the probability that the criterion selects a 1st-order process when the underlying generating process is $\mathbb{W}^0_\varepsilon$:

$$e_{\text{over}}(\varepsilon) := \mathbb{P}_{X^\varepsilon_{1:\ell} \sim \mathbb{W}^0_\varepsilon}(\text{argmin}_{r\in\{0,1\}}\text{CAIC}(Y^{r,\varepsilon}_{1:\ell}) = 1), \tag{47}$$

One reason why this can occur is that the perturbation may not respect the cluster structure, which can cause the clustered process $Y^{r,\varepsilon}_{1:\ell}$ to have higher-order dependencies even if $X^\varepsilon_{1:\ell}$ is 0th order. The underfit error probability is defined as

$$e_{\text{under}}(\varepsilon) := \mathbb{P}_{X^\varepsilon_{1:\ell} \sim \mathbb{W}^1_\varepsilon}(\text{argmin}_{r\in\{0,1\}}\text{CAIC}(Y^\varepsilon_{1:\ell}) = 0), \tag{48}$$

that is, the probability we select a 0th-order process while the actual underlying data-generating process $\mathbb{W}^1_\varepsilon$ is 1st-order.

We focus on the Deoxyribonucleic Acid (DNA) and Standard and Poor's 500 (S&P500) datasets. Because the S&P500 dataset is the least clear dataset, we also consider a synthetic observation sequence. This synthetic observation sequence is generated using the same model $\mathbb{W}^r_\varepsilon$ as is obtained for the stock market, but will be five times as long: 5ℓ with ℓ the length of original path of the S&P500 dataset. This "extended stock market model" gives a synthetic proxy to study the effect of sparsity on the criterion robustness *as if* we have access to more data.

Fig. 2 displays the error probabilities and centered CAIC values. We see that both the underfit $e_1(\varepsilon)$ and overfit error $e_2(\varepsilon)$ are small for small ε. The overfit error is, however, considerable larger for the DNA dataset than for the S&P500 dataset. This supports the claim that the CAIC chooses the model with fewest parameters for the

same amount of information and is hence less prone to overfit when the data is sparse. The underfit error is on the contrary small for the DNA dataset, also for $\varepsilon \in [0.1, 0.2]$. Taken together, this all suggests that order selection via information criteria is robust to small perturbations.

The case of the S&P500 dataset is especially interesting. In the results in [1, Table 4], the criterion chooses $r = 0$ whereas in the $\mathbb{W}^1_\varepsilon$ model in Fig. 2(a)–(b), the criterion selects $r = 1$ up to $\varepsilon \sim 0.1$. Afterwards, deviating from the BMC model by just 1 out of 10 jumps in the S&P500 dataset will make the criterion behave similarly as in [1, Table 4]. This is also supported by Fig. 2(b), where the difference between the criterion for $r = 0$ and $r = 1$ in the S&P500 dataset takes values in $[0, 10]$, which we coincidentally also see in [1, Table 4]. This suggests that there may be a 1st-order Markovian structure in the S&P500 dataset but also a strong 0th-order component. Alternatively, the data may simply be too sparse for order selection. The latter hypothesis is also supported by the synthetically extended dataset, where model selection has fewer problems.

7 Extra tools for some of the different data sets

7.1 The cf-idf vectorization method

Let $\mathcal{V}$ denote the vocabulary, which is a set of words, and fix a clustering $\sigma_n : \mathcal{V} \to [K]$. In order to turn documents into vectors we make use of a straightforward modification of the common *term frequency-inverse document frequency* document vectorization method, we refer to the modification as *cluster frequency-inverse document frequency* (Cluster Frequency–Inverse Document Frequency). Let $\mathcal{D}$ denote a collection of documents. Every document $d \in \mathcal{D}$ is here viewed as a sequence of words meaning that $d \in \prod_{t=1}^{\ell_d} \mathcal{V}$ for some $\ell_d > 0$. For every cluster $k \in [K]$ and document $d \in \mathcal{D}$ we define

$$\begin{aligned} \text{cf}(k, d) &:= \ln\big(1 + \#\{t = 1, \dots, \ell_d : \sigma_n(d_i) = k\}\big), \\ \text{idf}(k, \mathcal{D}) &= \ln\Big(\frac{\sum_{k=1}^{K}(1 + \sum_{d\in\mathcal{D}} \#\{t = 1, \dots, \ell_d : \sigma_n(d_i) = k\}}{1 + \sum_{d\in\mathcal{D}} \#\{t = 1, \dots, \ell_d : \sigma_n(d_i) = k\}}\Big), \\ \text{cf-idf}(k, d) &:= \text{cf}(k, d) \cdot \text{idf}(k, \mathcal{D}). \end{aligned}$$

Observe that cf-idf$(\cdot, d)$ assigns a K-dimensional vector to any document $d \in \mathcal{D}$. A word which contributes multiple times in a document also yields a higher contribution of the corresponding cluster to cf(k, d). Finally, words which are not in the vocabulary of the clustering are not counted at all in our processing.

7.2 Algorithm for creating a grid for animal movement data

Given a desired grid side length x (in kilometers), we can calculate the (regional) latitudinal and longitudinal degree corresponding to that distance x (assuming the earth to be a near perfect sphere). Latitudinal differences amount to the same distance in kilometers. One degree of latitude is 1/360th of the earths circumference (40 075 km) so one degree of latitude is equivalent to 110.574 km. Accordingly, x km are represented by $x/110.574$ degrees of latitude. One degree of longitude however represents a different

amount of kilometers, depending on the latitude: one degree of longitude is $|111.320 \cdot \cos_{\text{dd}}(\text{latitude})|$ km and x km are represented by $|x/111.320 \cdot \cos_{\text{dd}}(\text{latitude})|$ degrees of longitude, at a specific latitude. Here $\cos_{\text{dd}}$ is the cosine acting on decimal degrees. We are making use of a small angle approximation, which breaks down near the poles. The process of creating squares and assigning the units y_i to them is done in the following Algorithm 3.

Algorithm 3 Global Positioning System data to transitions between squares of side length x km

Input: Grid size x, Global Positioning System data $(y_{i1}, y_{i2}, y_{i3})_{i=1,\dots,n}$.
Output: Sequence of transitions between states $s = (s_1, \dots, s_n)$

$j_{\text{lat}} = \lfloor y_{11} \cdot 110.574/x \rfloor$
$j_{\text{long}} = \lfloor y_{12} \cdot 111.320 \cdot |\cos_{\text{dd}}(j_{\text{lat}} \cdot 110.574/x)|/x \rfloor$
$S_1 := [j_{\text{lat}} \cdot \frac{x}{110.574}, (j_{\text{lat}} + 1) \cdot \frac{x}{110.574}) \times [j_{\text{long}} \cdot |\frac{x}{111.320 \cdot \cos_{\text{dd}}(j_{\text{lat}} \cdot 110.574/x)}|, (j_{\text{long}} + 1) \cdot |\frac{x}{111.320 \cdot \cos_{\text{dd}}(j_{\text{lat}} \cdot 110.574/x)}|)$
$\mathcal{S} \leftarrow \{S_1\}$
$s \leftarrow (1)$
for $\text{index}_{\text{coord}} \leftarrow 1$ to n **do**
  for $\text{index}_{\text{square}} \in \{1, 2, \dots \#\mathcal{S}\}$ **do**
    added $\leftarrow$ False
    if $(y_{\text{index}_{\text{coord}}1}, y_{\text{index}_{\text{coord}}2}) \in S_{\text{index}_{\text{square}}}$ **then**
      $s \leftarrow (s_1, \dots, s_{\text{index}_{\text{coord}}-1}, \text{index}_{\text{square}})$
      added $\leftarrow$ True
    end if
    if added = False **then**
      $j_{\text{lat}} = \lfloor y_{\text{index}_{\text{coord}}1} \cdot 110.574/x \rfloor$
      $j_{\text{long}} = \lfloor y_{\text{index}_{\text{coord}}2} \cdot 111.320 \cdot |\cos_{\text{dd}}(j_{\text{lat}} \cdot 110.574/x)|/x \rfloor$
      $S_{\#\mathcal{S}} := [j_{\text{lat}} \cdot \frac{x}{110.574}, (j_{\text{lat}} + 1) \cdot \frac{x}{110.574}) \times [j_{\text{long}} \cdot |\frac{x}{111.320 \cdot \cos_{\text{dd}}(j_{\text{lat}} \cdot 110.574/x)}|, (j_{\text{long}} + 1) \cdot |\frac{x}{111.320 \cdot \cos_{\text{dd}}(j_{\text{lat}} \cdot 110.574/x)}|)$
      $\mathcal{S} \leftarrow \{S_1, \dots, S_{\#\mathcal{S}}\}$
      $s \leftarrow (s_1, \dots, s_{\text{index}_{\text{coord}}-1}, \#\mathcal{S})$
    end if
  end for
end for

The algorithm assigns all the Global Positioning System points to squares. This procedure may not work well if the animal is moving close to the poles, because the small angle approximation is not justified anymore. This procedure is also not particularly well suited for regions where the earth is not behaving like a sphere, for example if the animal in moving on mountains.

8 Raw data of, and extra material on, some of the datasets

8.1 Transition matrix for bison clusters

Below is the cluster transition matrix for the improvement clustering depicted in [1, Fig. 2], the numbers are rounded to the second decimal place.

$$\begin{pmatrix}
0.82 & 0.05 & 0 & 0.01 & 0.02 & 0.01 & 0.01 & 0.01 & 0.02 & 0.02 & 0 & 0.01 & 0.01 & 0 & 0 \\
0.07 & 0.76 & 0.02 & 0.03 & 0.05 & 0 & 01 & 0 & 0.01 & 0 & 0 & 0.02 & 0.02 & 0 & 0.01 \\
0.01 & 0.05 & 0.87 & 0.03 & 0 & 0 & 0 & 0 & 0 & 0 & 0.03 & 0 & 0 & 0 & 0 \\
0.02 & 0.06 & 0.03 & 0.85 & 0.03 & 0 & 0 & 0 & 0 & 0 & 0 & 0 & 0 & 0 & 0.01 \\
0.04 & 0.07 & 0 & 0.02 & 0.77 & 0 & 0 & 0 & 0 & 0 & 0 & 0 & 0 & 0 & 0.1 \\
0.04 & 0 & 0 & 0 & 0 & 0.88 & 0.02 & 0 & 0 & 0 & 0 & 0 & 0.03 & 0.02 & 0 \\
0.03 & 0.01 & 0 & 0 & 0 & 0.01 & 0.86 & 0 & 0.01 & 0 & 0 & 0 & 0.04 & 0.03 & 0 \\
0.06 & 0 & 0 & 0 & 0 & 0 & 0 & 0.84 & 0.07 & 0 & 0 & 0.01 & 0 & 0 & 0 \\
0.09 & 0.01 & 0 & 0 & 0 & 0 & 0.01 & 0.06 & 0.72 & 0.01 & 0 & 0.1 & 0 & 0 & 0 \\
0.06 & 0 & 0 & 0 & 0 & 0 & 0 & 0 & 0.01 & 0.79 & 0.1 & 0.03 & 0 & 0 & 0 \\
0.01 & 0 & 0.04 & 0 & 0 & 0 & 0 & 0 & 0 & 0.09 & 0.83 & 0.02 & 0 & 0 & 0 \\
0.03 & 0.06 & 0 & 0 & 0 & 0 & 0 & 0.01 & 0.1 & 0.04 & 0.02 & 0.73 & 0 & 0 & 0 \\
0.03 & 0.06 & 0 & 0 & 0 & 0.04 & 0.05 & 0 & 0 & 0 & 0 & 0 & 0.77 & 0.05 & 0 \\
0 & 0 & 0 & 0 & 0 & 0.05 & 0.07 & 0 & 0 & 0 & 0 & 0 & 0.14 & 0.72 & 0 \\
0.02 & 0.03 & 0 & 0.01 & 0.31 & 0 & 0 & 0 & 0 & 0 & 0 & 0 & 0 & 0 & 0.63
\end{pmatrix}$$

8.2 Groups of words for improvement with 200 groups

8.2.1 Document classification datasets

We here describe the datasets which are used to construct [1, Table 2] and report on some other datasets where our findings are inconclusive in Table 1.

AG News.

This dataset provided by [14] consists of tuples (x, y, z) where x is the title of a news article, y is a description of the news article and z is an assigned class. There are here four possible classes which z can take as values namely *World*, *Sports*, *Business* and *Sci/Tech*. For each such class the dataset contains precisely 30 000 training samples and 1 900 testing samples. In our processing we concatenated x and y into a single string and the task is to predict the class label z based on this string.

Yahoo!.

This dataset provided by [14] contains questions and answers from *Yahoo! answers*. The dataset consists of tuples (x, y_1, y_2, z) where x is a question, y_1, y_2 are answers to this question and z is category to which the question belongs. It can here also occur that the question has fewer than two answers in which case y_1 or y_2 is the empty string. There are ten possible classes which z can take as values namely *Society & Culture*, *Science & Mathematics*, *Health*, *Education & Reference*, *Computers & Internet*, *Sports*, *Business & Finance*, *Entertainment & Music*, *Family & Relationships* and *Politics & Government*. For each such class the dataset contains precisely 140 000 training

samples and 5 000 testing samples. In our processing we concatenated x, y_1 and y_2 into a single string and the task is to predict the class label z based on this string.

Wiki.

This dataset comes from the DBPedia ontology project [15] and the precise version used here is constructed by [14]. The dataset consists of tuples (x, y, z) where x is a title of a Wikipedia page, y is the abstract of the page and z is the category to which the page belongs. There are 14 possible classes which z can take as values. For each such class the dataset contains precisely 40 000 training samples and 5 000 testing samples. In our processing we did not use the title x so the task is to predict the class label z based on the abstract y.

Book.

This dataset is constructed based on books from Project Gutenberg and their genres are assigned on GoodReads, the dataset was obtained from [16]. The dataset contains tuples (x, y, z) where x is the tile of a book, y is the full text of this book and z contains a set of genres. We only retained those data points for which z is a set with a single element from one of the following six categories: *cookbooks*, *fantasy*, *horror*, *politics*, *religion* or *science-fiction*. We further randomly selected 2 000 training samples which left 387 samples for testing. In our processing we did not use the title x so the task is to predict the genre z based on the text y.

CMU.

The CMU Book Summary Dataset contains plot summaries for books which are extracted from Wikipedia by [17]. The dataset contains tuples (x, z) with x a plot summary and z the category to which the book belongs. We retain all datapoints whose category z occurs at least 50 times which leaves us with two genres namely *Fantasy* and *Science-Fiction*. We randomly select 1 138 datapoints for training which leaves us with 380 testing samples. The task is to predict the genre z based on the summary x.

20news.

This dataset contains newsgroup postings for 20 different newsgroups which are collected by [18]. The dataset is accessed using the function `fetch_20newsgroups` from `sklearn.datasets`. The dataset contains tuples (x, z) where x is a message sent to the newsgroup and z is the label of the newsgroup. There are 20 possible classes which z can take as values. There are 11 314 training samples and 7 532 testing samples. The task is to predict the newsgroup z given the message x.

Spam.

This dataset contains text messages which are either legitimate or spam, collected by [19–21]. The dataset is accessed from [22] and contains tuples (x, z) where x is a text message and z is a label indicating if the message is spam. The possible values for z are spam or ham. The task is to predict z given the message x. Unfortunately, due to a mistake, the experiment is executed without splitting the dataset in training and testing samples. This means that the 4 179 available samples are used both during

training and testing. Splitting in testing and training would however not change the inconclusive conclusion from Table 1 and another experiment with a test-train split is not executed.

Reuters.

The Reuters RCV1 corpus [23] consists of a collection of news stories and was accessed using `nltk.download(´reuters´)`. The dataset contains tuples (x, z) with x a news article and z the category to which it belongs. There are 58 possible values for z. There are 6 577 training samples and 2 570 testing samples. The task is to predict the category z based on the text in x.

Table 1 Results for performance on document classification where neither method significantly outperformed a random clustering.

Algorithm	20news	Spam	Reuters
Random $K = 50$	23.2%	86.4%	65.4%
Spectral $K = 50$	23.0%	86.1%	63.0%
Improved $K = 50$	25.2%	86.1%	63.3%
Random $K = 100$	31.0 %	86.7%	67.7%
Spectral $K = 100$	31.1%	86.9%	66.2%
Improved $K = 100$	33.6%	87.2%	68.9%
Random $K = 200$	38.0%	87.2%	68.4%
Spectral $K = 200$	36.2%	87.0%	69.0%
Improved $K = 200$	40.2%	87.2%	70.8%
Random $K = 400$	44.7%	87.6%	87.8%
Spectral $K = 400$	41.4%	87.7%	88.0%
Improved $K = 400$	43.9%	87.7%	89.0%

8.2.2 Detected groups

Here are the detected groups when using the cluster improvement algorithm:

$\mathcal{V}_1 =$ mauser, blackout, wari, yak, sprite, puff, nightlif, capitalis, vhf, shroud, athena, featurelength, workflow, fright, grasshopp, misunderstood, aeroplan, farreach, prequel, ascii, veterinarian, heyday, metalwork, timeout, nod, cavern, nf, utilitarian, chevi, ting, aphrodit, unsatisfactori, pieti, inund, heist, cl, fullfledg, autopsi, intang, deregul, hyphen, hdtv, gild, majest, nasti, discretionari, computer, ambival, invinc, ide, rt, radiohead, brainwash, slur, teaser, pl, indepth, outag, pak, rebirth, pun, barksdal, crypt, outtak, crosscultur, blaster, spit, simplist, amt, bn, bd, bikini, dea, miscarriag, reenact, makeshift, synergi, uninterrupt, surrealist, toolkit, pervas, shini, dismal, dizzi, spire, dm, lancia, hadrian, viacom, foal, hippi, bonnet, subplot, cfa, poseidon, inhuman, ecstasi, drawer, subaru, diminut, til, amiga, beggar, yoke, twinengin, redefin, stomp, giraff, elisa, preambl, servitud, ridership, thoroughbr, miser, lingua, medusa, unreal, gl, delinqu, garuda, equit, earmark, tesco, §, nb, bogi, dod, sx, impromptu, balconi, fastbal, ingam, coerciv, adjunct, carib, embezzl, disrespect, smuggler, bitch, freestand, slipper, netscap, textual, vp, glam, highestr, falsifi, facetofac, shadi, yamaha, cradl, sceptic, londonbas, weari, utopian, sigmund, contenti, counterfeit, medley, vigilant, weakest, superimpos, fg, retel, solstic, vibrant, tapestri, martian, illustri, lander, reevalu, kneel, involuntari, jug, hl, dingo, eel, mn, wealthier, earner, affection, learnt, mermaid, tempest, nz, rhapsodi, astrophys, wiki, vaudevil, wager, leaflet, dazzl, approx, bloodsh, ode, rung, shovel, lorri, indiscrimin, proudli, xs, ata, pastim, bane, dar, unidentifi, usda, climber, idiom, sabl, gogo, purs, klan, threeday, withhold, remington, cling, shouldnt, agoni, delin, applaus, plagiar, toughest, meta, mingl, profan, tc, interdict, requiem, shutdown, participl, synth, jab, meme, misl, gaia, hightech, buoy, contriv, despis, sceneri, mimick, labyrinth, larval, fsa, panelist, tangibl, ns, geophys, simplif, geforc, selfhelp, mv, amulet, hurdl, midsiz, delimit, underag, animos, hegel, goofi, afi, preoccupi, sinner, cheetah, ict, waiver, panda, timet, ate, wand, kar, levit, foreshadow, voucher, batsmen, strabo, interscop, styliz, charisma, exploratori, hyundai, kitten, reaper, redress, tabul, terrif, vindic, warranti, hitch, manx, blink, hiro, boomerang, fantasia, hieroglyph, gloss, galley, jumper, remit, industrialis, idiot, safari, crunch, linger, wrc, macaqu, emplac, biker, xxx, retort, uh, fao, carp, endus, playground, michelin, viewership, meteor, fw, sf, toon, greenpeac, flamingo, sleepi, ordinarili, overton, fd, gamer, pluto, shill, primal, rattl, tg, kawasaki, northrop, ser, unseen, cola, backfir, tricki, confound, slick, cfr, purportedli, knuckl,

erp, amish, shrew, cad, deterr, sco, ju, spaceship, godzilla, bitten, supervillain, intrud, thunderbird, boogi, mammoth, hg, tt, annot, sway, quip, splinter, videotap, vj, namepl, scarf, mf, teleport, panorama, treacher, downplay, aircrew, pedigre, brahman, loki, gmt, cordial, gal, souvenir, asiat, limp, virtuou, rebuk, barrag, unfavor, extort, exhort, kde, vr, manhunt, hiss, memorabilia, rsa, conglomer, twodoor, woodwork, expend, gauntlet, leech, acp, stallion, elv, appendix, artefact, dime, prophesi, rambler, anteced, expropri, overtur, curli, bm, richer, wellreceiv, devour, oc, expressli, glyph, gull, reemerg, swimsuit, puppi, nu, stumbl, overrid, ub, narcissist, selfdestruct, ontolog, geni, tekken, nec, mya, payabl, threequart, thug, utterli, latenight, fallout, worthwhil, cpm, superpow, swiftli, eco, jellyfish, usn, joystick, spoon, grim, gimmick, irc, clarif, figurin, caregiv, hesh, nearer, dsm, laughter, hum, slider, hannabarbera, easiest, sg, caricatur, honesti, humankind, calypso, constru, irrespect, utmost, deceit, dislodg, antic, finalis, subconsci, surfer, alterc, maze, azur, ska, packard, sari, conduc, sar, reconnect, stagnat, undead, starscream, sculpt, , poach, psp, geopolit, seaplan, fallaci, untitl, unbalanc, sticker, fiasco, dentistri, intimaci, stormi, wc, ui, outcrop, friez, triplet, bald, vertigo, bot, courtship, superb, rl, ansi, sash, footwear, interrel, gemston, doorway, cynic, evoc, kodak, insecur, alchemi, disobey, aac, devalu, neo, cohort, longestrun, hairstyl, jtwc, stasi, ipo, bh, descriptor, oratori, atf, porcelain, foolish, briberi, vase, fireplac, nourish, electronica, institution, widest, cisco, allnew, cute, seduct, dummi, msdo, sweater, rss, witti, mallet, mazda, unfit, snare, oem, anoint, raspberri, scorn, highaltitud, debtor, habitu, peril, rockstar, reckless, sturgeon, combo, theyv, unravel, townspeopl, bf, wildli, offlin, bleak, chameleon, dogg, lsd, videogam, pon, fret, spaciou, spaniel, allround, garnet, livelihood, info, savior, kelvin, wither, arson, twa, lte, lowbudget, taker, ova, attende, lamborghini, stalem, sikorski, funerari, harem, pillow, forfeit, middleearth, embroid, cv, interdepend, underestim, lawless, seamless, sag, shrapnel, thee, sci, plummet, rollsroyc, pythagorean, olympu, expound, incens, centralis, ipcc, airtoair, rake, pantomim, queer, atv, vaniti, nsa, repercuss, asp, transpos, internship, domesday, hypnosi, encycl, lifeboat, hibern, cloak, hyena, lager, sic, unsolv, worldli, chomski, vulcan, stealth, pep, blasphemi, entrepreneuri, msc, paw, burlesqu, nuisanc, eta, backyard, dumb, freemason, clearer, dreamcast, icbm, heartbreak, lego, sewn, shun, decrypt, miscellan, feral, sarcast, toru, gunmen, omiss, lad, forefront, timbr, cdrom, dread, authorit, brink, feign, plutarch, oct, squat, gunfir, grecoroman, naiv, stagger, toast, derid, antler, apa, loophol, misdemeanor, tunic, tnt, cpr, issuanc, magnolia, fearless, preposit, nitro, hid, ua, rioter, nr, chiropract, dagger, notoc, mist, pixar, kosher, enron, encor, carnat, thirdperson, gunman, cliché, minion, unreason, palladium, ticker, overtli, republish, notif, vacanc, punit, kraft, bosch, salsa, tracker, diy, geek, adjourn, supercomput, harp, goblin, hatchback, tub, whichev, eyebrow, octagon, unspecifi, folli, bombardi, questionnair, pf, undevelop, bop, hardtop, regal, haplogroup, salesman, harrier, quak, willingli, okay, crumbl, salient, infrequ, bailout, britannica, choreographi, shit, npc, phobia, reborn, groundwork, octopu, rum, fric, airship, gr, hone, luger, humil, homo, onesid, echelon, viet, wardrob, gazel, nonn, opel, crossbord, unlicens, locust, catchi, revolution, cherish, rh, façad, inconclus, exalt, hex, towel, mummi, hostess, prowess, monograph, centaur, mozilla, bidder, deepwat, moog, impass, lifes, partak, aristotelian, unheard, fad, gw, ornat, seren, rhino, rove, awak, motorist, chilli, underdog, downgrad, illiter, apprenticeship, gadget, californian, sportsman, makeov, dissatisfact, eman, chopper, isa, wikileak, coalesc, deepest, fullscal, tiein, wig, pathologist, melodrama, empower, centerpiec, telepath, lite, protract, deu, minigam, robber, twentytwo, jeopardi, buff, sodomi, unsur, valiant, mute, powerless, amg, stump, pygmi, volley, lavish, naa, energ, penc, afterlif, ax, dogmat, weasel, playlist, apparel, moos, ascertain, bi, oss, deliver, epitaph, , newslett, klingon, snatch, raccoon, unbroken, iliad, trivia, disillus, ol, flick, dude, twoweek, solitud, montag, uncut, howl, everlast, tl, phonograph, beforehand, rp, aol, proverb, crucifixion, audiovisu, hb, topless, sutra, overweight, retro, toad, spar, distinctli, minimalist, jerk, laps, sear, license, synthesis, goon, ntsc, arbitrarili, drape, wrought, borderlin, selector, necklac, mileag, reap, lick, wrapper, nymph, orc, peerreview, pip, destabil, hurri, courtesi, biospher, fax, reassur, surreal, therein, bra, cock, statur, handi, sentri, upsid, intermediari, sensual, iucn, publicis, acdc, tranquil, glanc, biplan, eloqu, backlash, focuss, dismount, coloss, extracurricular, widescreen, topdown, roar, technicolor, pictori, quiz, hyster, neapolitan, oneman, rebroadcast, polygami, underscor, vedanta, funki, shorthand, interdisciplinari, tamper, spelt, pdp, perch, reexamin, jingl, voc, subpoena, exhum, kettl, elektra, culprit, hallway, ital, silo, lovecraft, divest, flamenco, budgetari, fuzzi, almighti, assail, decoy, aptitud, septuagint, turntabl, impal, underwrit, prepaid, intro, leve, dice, glare, nurtur, nirvana, gunfight, readership, indigo, maniac, concoct, wow, minaret, pelt, miracul, excurs, transnat, behold, witchcraft, sleeper, mirag, mercedesbenz, fest, personifi, lastminut, daimler, stud, dada, limousin, baffl, platon, confuciu, symposium, objectori, cx, whiski, parrot, troublesom, swarm, biometr, seabird, preclud, lunat, speedi, sunglass, attic, merlin, closeup, sober, sha, resuppli, categoris, blight, maximis, rg, inr, unfamiliar, payoff, devoid, bonus, acrobat, mash, rook, nyse, utensil, coercion, soyuz, twig, champagn, enigmat, imparti, colossu, mindset, allig, detractor, preproduct, hump, deadliest, immor, skinni, tuner, irrespons, odysseu, vo, hitter, cybertron, geo, ara, backstori, sh, righteous, tester, disorgan, kingship, ipa, explanatori, thrash, selfcontain, oo, nk, annuiti, af, handson, resel, themat, electrif, twentyon, scam, superhuman, compuls, dissatisfi, clinician, onslaught, indign, disdain, sublim, shove, handler, kippur, payout, panoram, mana, habea, hedgehog, joker, pg, totem, myriad, straighten, allegor, urgenc, calf, outgo, quilt, acacia, apprais, subgenr, curtail, pol, pup, unicorn, decri, misti, leibniz, mardi, autist, galact, vibe, collag, junip, symbolis, breakout, embellish, propens, dire, shortfal, rudimentari, paralyz, meticul, riddl, machinegun, snp, fi, lupin, quorum, disparag, fetish, masquerad, monologu, oar, copul, dl, theyd, spec, highdefinit, unnot, bernoulli, lowercas, dx, slump, countercultur, barbecu, impregn, inaccess, intellect, hoop, ppp, windmil, junk, anu, catchphras, wilt, isoiec, hideout, ¥, lash, curtiss, terra, tighter, someday, trebl, brawl, pandora, behindthescen, stag, pejor, existenti, debit, kc, cyclop, firefox, nvidia, grudg, epithet, fuell, mela, ancillari, wrongli, †, lastli, guillotin, firstgener, sadli, gtr, solemn, roadsid, ur, shopper, oneshot, finder, slew, tramp, sl, mutil, ri, linnaeu, hoist, gorgeou, esteem, boar, cider, sled, whistleblow, lowpow, handmad, surrog, electro, whereupon, peugeot, abstain, lavend, endear, instil, verizon, priestli, embroil, horsesho, tenyear, monolith, obvers, outset, limbo, feroci, capcom, cowl, shaker, wreckag, implicitli, bracelet, homeown, optimu, twentythre, tripod, inocul, goodi, uplift, rejoic, sideway, overdub, jackpot, aclu, ee, chore, permeat, sevenyear, psychiatri, tranc, immacul, codex, bsa, cbi, mono, newfound, sparrow, nightli, cessna, coca, hospic, priestess, advert, nil, monstrou, ridden, lute, anthrax, yam, unhealthi, luthor, cosmopolitan, directv, superstit, mink, catfish, captor, imax, dwindl, sprung, veda, grotto, indispens, tauru, uhf, dt, grail, parabl, gopher, bayonet, longev, enix, pepsi, honorif, barter, centauri, inquest, dy, maharishi, aton, pud, pulsar, mic, outcast, poincaré, reinvent, bk, voiceless, voiceov, pumpkin, britney, underlin, nike, zodiac, nostalg, fokker, rejuven, magneto, tabloid, standoff, fang, authorship, toddler, acl, rapist, clumsi, glossi, goliath, avi, deconstruct, rhinocero, subterranean, gpl, dlc, ·, stride, cgi, entangl, firefli, tor, humanoid, postproduct, vane, blew, bootleg, bluegrass, hawker, kickstart, flea, tortois, kr, helper, prerequisit, entrylevel, thinli, sorcer, philanthrop, pegasu, rein, hacker, jive, binocular, ovid, interlac, kin, enigma, dat, transpir, suv, punctuat, parlor, pinch, preset, alchemist, wouldb, pharmacist, piti, sentient, gangsta, spectacl, mattress, plough, melancholi, sender, adida, subchannel, stigma, watcher, valor, trespass, accru, windi, infanc, konami, passer, whiskey, billiard, interraci, hord, yeah, applaud, hera, medallion, antelop, pax, paranorm, starbuck, elf, tlc, kepler, hysteria, dusk, landbas, passov, nostalgia, snoop, tr, issuer, bl, anxiou, kite, fairchild, scifi, raisin, entrepreneurship, protestor, mahogani, womb, perl, hk, vegan, frantic, bastard, covet, turboprop, urn, corvett, stela, sentinel, agnost, winfrey, youngster, pelican, requisit, coop, odyssey, tacitu, unharm, hypocrisi, wellestablish, upanishad, bodywork, parenthes, rj, puberti, misspel, reckon, auspici, sapien, curfew, cymbal, twopart, loom, blacksmith, pancak, multilingu, egalitarian, dp, unambigu, viper, assemblag, turnaround, rn, cull, weep, peach, autobot, impati, participatori, volvo, awesom, ericsson, derail, mayhem, atrium, recuper, hen, oprah, leftov, omnibu, saab, frenzi, oedipu, circumv, marconi, xmm, unfairli, summaris, puck, reassembl, paranoid, whatsoev, intertwin, department, stargat, mx, sip, namco, unbeliev, sig, facelift, hype, relentless, chakra, jewelleri, supplementari, chime, markup, torrent, generos, shortcom,

deciph, chaser, tartan, guevara, carriageway, extraordinarili, spaceflight, disengag, impetu, farc, comprehend,
shipwreck, galileo, poorer, bw, execution, unmark, compliant, winemak, din, horseback, clockwis, stupa,
creas, tulip, strangl, conscienti, outperform, lexu, glamor, tougher, stricter, sandal, inexperienc, com, hourli,
payrol, jag, secondhand, suffic, reconfigur, diaper, groundbreak, neat, secondgener, rko, effigi, tweak, ton-
nag, cryptic, fetch, hsv, supergroup, affidavit, bg, cun, grumman, uncanni, unleash, disallow, jg, braveri,
pervers, folio, ratchet, impedi, um, orat, mnemon, holist, disgrac, earnest, gall, ati, newt, tata, webbas, rune,
hog, brandi, bandag, baccalaur, gallup, fowl, priorit, plank, ku, misfit, eo, csa, rooftop, bonfir, draught,
quirki, hydra, vm, paranoia, loco, quan, reliant, wd, pitchfork, sprinkl, lakh, coda, heap, hasten, harshli, xl,
materialist, miseri, orderli, mundan, sprang, dreamwork, emu, iaea, patienc, heron, twoday, lax, undoubt-
edli, unison, stutter, barcod, hooligan, longlast, hitherto, ass, sadist, staircas, exoner, dupont, saucer, nou,
dolbi, psychopath, cellar, mele, volta, preorder, bodhisattva, pap, voltair, splash, techno, avert, gallop, fieri,
char, amazoncom, smear, aura, wildfir, suitcas, discord, worshipp, gnome, dissimilar, inconveni, substant,
scrambl, detour, overcrowd, mismanag, gin, treacheri, quadrupl, cambrian, extravag, cookbook, splendid,
derelict, masteri, breastfeed, sixmonth, scari, stubborn, triton, domino, tempt, resuscit, standardis, mca, fn,
consumm, thunderbolt, apron, cctv, diphthong, cajun, nuditi, esperanto, monochrom, decidedli, reboot, flop,
commonplac, overs, nra, masturb, shank, josephu, barren, kinship, acquitt, rosari, mediocr, assort, unpro-
tect, lien, apparit, cg, liar, multitud, gpa, factual, proprietor, typifi, rx, pimp, emphat, transgress, outdat,
cauldron, esquir, astound, tug, biscuit, handicraft, rescuer, novic, ls, sacrifici, broom, extraterrestri, cinemat,
primu, contemporan, lightheart, nontradit, ddt, heroism, slant, handtohand, deepen, fictiti, yamato, bale,
mug, metaanalysi, papyru, incapacit, concis, rewritten, subvert, blueprint, oneoff, taglin, engulf, atc, attrit,
pretens, loath, recordbreak, typewrit, bun, interceptor, selfish, shack, indec, bharat, peta, upstair, mace,
louder, fy, coupon, genitalia, malic, gambit, salamand, untouch, hottest, retitl, fisherman, tyrannosauru,
flamboy, handwrit, appal, matador, rendezv, solari, coproduct, sapphir, astra, reclassifi, hindustani, aoc, uri,
progenitor, threemonth, apocalypt, untru, astral, rv, twoseat, stricken, scotch, bullion, improperli, heracl,
outburst, tp, onehalf, hasnt, antagon, ruse, icao, neptun, rad, sparkl, nypd, mart, asa, ero, discriminatori,
grotesqu, javelin, beginn, macroeconom, housew, etho, clover, ramadan, iss, invalu, disprov, afloat, typefac,
rebat, worldview, freighter, gees, hermit, iaf, lame, haze, dynamit, eclect, lingeri, interlud, tout, careless,
precari, discover, reinterpret, peacetim, equinox, unplug, inflight, monik, racket, oldfashion, reputedli, tack,
orb, unnatur, troll, nam, interspers, burglari, digger, wallet, tame, uneth, galactica, shard, ingeni, misinter-
pret, apocryph, cannonbal, pn, precept, shaken, thale, compassion, iec, prologu, epistemolog, shuffl, buyout,
stare, drinker, pinnacl, booklet, mta, reintroduct, dilig, selfconsci, gs, extrapol, slack, ebook, citroën, refund,
stardom, turban, gorilla, upbeat, spinner, cautiou, bellow, decca, viennes, hardcov, stave, scooter, stretcher,
euthanasia, anti, recast, operat, pli, buick, remad, eb, tesla, acorn, conjur, forgeri, onetim, pleistocen, needi,
resurfac, onehour, cinematographi, undetect, ama, solidifi, fabul, stat, backpack, epitom, motorola, mouth-
piec, diversif, swastika, wheelchair, brightest, yom, phalanx, multidisciplinari, lore, transpond, profoundli,
pd, anarchi, internation, chinook, albatross, mu, westinghous, tombston, timeless, scoop, exquisit, gunneri,
funniest, contextu, midday, nighttim, pluck, ×mm, tuck, lampoon, concur, orchid, sire, worthless, kia, tore,
foreclosur, mainstay, corset, calligraphi, antidot, cryptographi, disarma, xerox, hastili, resumpt, castor, fugu,
cuckoo, retribut, glorifi, apc, peg, foray, birch, lieu, introspect, mau, chequ, wreath, hitchhik, pew, spread-
sheet, dropout, bulldoz, iu, abrupt, loft, lucif, oa, caption, pe, beret, uneasi, penthous, lü, supplant, dh, entic,
watchdog, neanderth, americana, taint, ot, jargon, pu, reclus, pinki, eater, silhouett, nov, féin, rampag, snap-
shot, gass, pri, flatter, carousel, msa, forprofit, deadlock, seclud, fiddl, brokerag, skit, dualiti, mahal, ture,
derogatori, stout, odin, chai, evas, fleetwood, adulter, cyber, rehab, muppet, lex, elus, nonverb, raptur, mar-
tyrdom, aug, unjust, falun, keyston, nuanc, ordeal, headphon, chaotic, brillianc, penanc, saffron, hh, gentli,
edific, blaze, plung, slipperi, lexicon, standpoint, dubiou, unintent, gunshot, forgot, ro, keynot, underdevelop,
preemptiv, futil, succe, aqua, burgeon, fourwheel, reprimand, rye, eboni, asham, cadenc, append, multi,
dionysu, glimps, blizzard, stripper, stylu, misconcept, éireann, rarer, roost, unsign, hasbro, gc, shook, lust,
priu, orion, megatron, enchant, rem, lg, fanfar, dike, keynesian, polem, sciencefict, eyesight, mag, irrat, var,
opengl, unorthodox, ito, rampant, downturn, coerc, popularis, bohr, selfproclaim, ohm, eid, artemi, sonnet,
glitter, cocktail, disassembl, outcri, jeep, trash, promiscu, hypnot, gown, layoff, reconsid, recollect, netflix,
misfortun, ubuntu, melon, dinar, fukushima, tabernacl, hertz, brute, codec, dáil, kingfish, psych, renegad,
infidel, parisian, elucid, picnic, paraphras, freemasonri, booti, firstperson, ak, paperwork, foo, subvers, vc,
wrongdo, steak, insolv, rocker, interplay, scholast, looney, amic, rancher, cracker, tn, thaw, yearlong, colli,
barbar, obelisk, scoreboard, overt, plow, loudli, vf, corona, illfat, gita, moonlight, twoway, firebal, ichigo,
couch, abod, gundam, symphon, chute, lush, crate, kd, acm, sed, psa, magellan, tyrant, sow, pedest, gambler,
craze, embroideri, vt, karaok, apprehens, adept, lr, botani, hindustan, cu, unexplain, transsexu, grit, yahweh,
youll, orangutan, slander, valkyri, diner, alt, calv, meander, preempt, tutori, rustic, mover, polari, sloth,
ponder, introductori, reshap, kierkegaard, tm, gigant, archeri, crisp, zedong, stairway, uav, skid, rariti, zeta,
interestingli, checker, eschew, htc, ramayana, kiwi, standbi, glaciat, reindeer, notforprofit, causat, ecommerc,
repaint, pilat, juror, anvil, abridg, sauron, trident, quad, magpi, catapult, franca, indetermin, viz, foot-
not, shortcut, horrif, meaningless, pinpoint, neoliber, werewolf, audiobook, accordion, tith, disclaim, wiener,
backer, triumphant, biomed, thistl, showroom, curricula, manslaught, crossbow, roadster, postcard, cock-
roach, blackandwhit, glitch, drm, psychotherapi, cactu, klux, kanji, kiosk, npr, moratorium, breez, overdr,
dentist, bystand, workout, underpin, psychoanalysi, dreadnought, taj, delphi, woe, woodpeck, syncop, ib,
dialog, counterpoint, expressionist, adulteri, cohabit, overpow, lancer, dunlop, elud, abyss, adverb, retroact,
guis, whenc, conflat, dab, fragranc, childbirth, handbook, firsthand, unrestrict, smoothli, disrepair, barefoot,
impressionist, ici, poke, contradictori, glad, quarantin, castrat, lest, br, decepticon, phish, overtak, omen,
crucifi, tangl, shapeshift, surmount, qa, ig, zenith, refit, tango, cyborg, widerang, talon, subprim, bland,
bugl, chernobyl, alias, phenomen, gmc, stylish, unrealist, silli, unearth, fanbas, sling, healer, accentu, remors,
ww, mania, gratitud, vignett, tantra, maraud, atheism, bac, signag, sunken, pti, corrobor, epilogu, mil, mbc,
sidebysid, downsiz, kangaroo, dormant, midi, slid, hoc, kt, backstag, inton, stringent, delici, scribe, nikon,
comma, pr, bhakti, spectr, rediscov, promo, pixi, helpless, hive, sociopolit, stray, apt, fanat, trapper, box-
offic, vultur, infal, mantra, subcultur, mime, desol, proactiv, fullsiz, skype, autograph, keel, overcam, idealist,
fourdoor, heartbeat, mattel, pasteur, emerald, csi, hopeless, elaps, envisag, rabi, mahabharata, hug, vulgar,
delus, outsourc, satisfactori, scuttl, noncommerci, boomer, maru, remiss, lm, nexu, mane, esp, psychoanalyt,
ale, suffoc, pois, almanac, etiquett, transcendent, euclid, restat, bondag, tintin, morph, inaccuraci, unau-
thor, noncombat, falter, earthli, repaid, pda, henceforth, obliter, cadillac, swirl, dd, fledg, epoch, manoeuvr,
displeas, vehicular, forese, allegori, oat, widget, maximu, miocen, aegi, fend, kabbalah, manic, skunk, gaze,
piraci, chevron, stifl, shabbat, improb, clap, unconvent, np, bmg, nasdaq, sucker, apprehend, sae, molotov,
withheld, poss, fsb, alevel, zebra, vip, horu, tusk, hs, messerschmitt, elk, goos, harlequin, reced, hourlong,
drank, usbas, invoc, ebay, dichotomi, potion, subsum, electra, teas, cardboard, ironi, gcse, awe, wutang,
envi, insofar, jackal, grappl, incest, hobbyist, sampler, vet, starship, soc, cliqu, typolog, casket, isp, bmi,
cumbersom, schemat, insignific, delicaci, beech, grung, misrepres, paramed, overshadow, closet, torment,
asu, laplac, stork, panason, rag, gamecub, phenomenolog, expon, policymak, mojo, esa, che, cybernet, afp,
nestl, walmart, exemplari, twohour, xx, handwritten, nerd, polka, ethnograph, antisubmarin, portmanteau,
notwithstand, freshli, toc, courier, referr, stewardship, kei, worldclass, foreground, disqualif, loneli, torch-
wood, enquiri, havoc, unpublish, unsettl, hobbit, swung, selfsuffici, beagl, gra, fab, accustom, druid, vodka,
foundri, crave, abound, rubl, preschool, malici, righteou, obligatori, reimburs, condon, millennia, yesterday,
hoard, centurion, sinist, junker, personif, sli, decapit, adc, voodoo, herm, waterg, kindl, attir, abelian, dowri,
greed, yearbook, endgam, throwback, msn, camper, nag, cappella, ia, hardest, adjud, glamour, undo, hearth,
semicircular, monopol, dew, ks, rubbl, utopia, greedi, dsp, ccc, futurist, courtroom, rr, medicaid, blacklist,

telegram, lazi, kink, sinn, symbiot, hade, php, outing, nazareth, scissor, minstrel, unresolv, subsect, belliger, naughti, siren, uranu, dissuad, veer, prometheu, restitut, stateoftheart, sprinter, prerecord, cleanli, maimonid, exceedingli, overtaken, coupé, ddr, barricad, anthropomorph, ope, empathi, neuter, notebook, cessat, nullifi, ox, shortwav, suitor, bandai, scorpion, startl, richli, underwear, bae, daredevil, horsemen, tumbl, doomsday, cong, arisen, pinbal, visionari, kayak, thirst, peertop, graveyard, diacrit, oldsmobil, spaghetti, bitterli, poppi, displeasur, manli, tardi, ei,

$\mathcal{V}_2 =$ cher, bj, constanc, dani, bartlett, rene, melvil, rowl, barth, ryder, stephenson, hitchcock, kendal, brahma, elisabeth, burt, polli, foss, craven, manni, kerr, berni, klau, alexandr, benoit, petri, bernstein, upton, rei, parri, maci, russ, townshend, fei, elop, baptis, gabe, melvin, osbourn, osman, sita, meng, stepmoth, dusti, ein, wheeler, beckett, elain, becki, jai, descart, slade, khrushchev, bigg, weir, kaplan, bingham, elena, pahlavi, kissing, adolph, guthri, dramatist, griev, mayfield, thornton, eliot, kang, sisterinlaw, voldemort, susi, mcgrath, cynthia, rahul, ame, xiang, chong, lazaru, mahmoud, priscilla, berg, paterson, fatima, silverman, cale, smokey, jakob, eno, sampson, cassidi, baum, elmer, partridg, tong, mildr, guan, harlan, mcbride, cabaret, mccoy, karim, serg, eastwood, reilli, gee, jacobi, croft, neumann, sgt, joann, meg, reggi, zane, bongo, cullen, vito, sax, trotski, bhatt, gilmour, nan, pam, lamont, braxton, ryu, vaughan, kobe, wesson, tyson, mcintyr, lea, hillman, capt, roth, vicki, boyl, emeri, brandt, marquess, earnhardt, zappa, olaf, sutton, kaiser, astor, nikolai, ringo, ashok, minogu, bard, stacey, mcclellan, calvert, kramer, hagen, cartwright, modi, farrel, walden, bai, ella, anand, ulyss, exchequ, sargent, kamal, fiancé, jacquelin, godfrey, custer, jacobson, brezhnev, childless, louie, madden, jawaharl, busch, humphri, jonni, anwar, donaldson, filmographi, draper, goddard, kirbi, huey, grimm, sherri, cbss, flair, nath, aerosmith, begum, lowel, milo, zhu, emmanuel, abbott, jamess, fatherinlaw, patterson, linu, seaman, deacon, woodward, shortlist, heartbroken, abram, rori, vera, horowitz, lorrain, joli, indra, mehm, reluctantli, kabir, rosenth, ste, mckenna, coleridg, boo, scarlett, aguilera, jinnah, bellami, dent, remarri, pei, lam, carla, meredith, wilbur, dodd, courtney, rishi, sanford, newsweek, ell, henrik, annul, gregg, mcgovern, lott, poe, bridget, hale, joplin, giuliani, gerhard, ramon, heidegg, biopic, serena, ulrich, herod, illegitim, tiffani, braun, dorian, concubin, ridley, dre, robbin, trombon, bundi, hanson, tun, epstein, sylvia, hooker, josef, titu, obituari, nme, dariu, vanc, miln, ty, rudolph, exclaim, sadi, thatcher, dion, hodg, prima, rajah, henrietta, davenport, dowag, dorsey, rousseau, manu, taunt, kubrick, mahmud, radha, herzog, cecilia, sigismund, tobago, hayn, maha, djokov, reza, daryl, kathleen, johan, beal, obo, valentino, pandava, armand, whitlam, arjun, orton, hui, ewe, lulu, heinz, sardar, shakira, rani, tinker, trier, pia, kern, jp, hai, reev, unita, lal, sumner, ke, bint, coltran, cabot, weston, hine, fullback, anastasia, jefferi, anita, aloud, britten, gordi, whereabout, woolf, quintet, halen, robson, picker, tilli, stafford, olivi, vikram, ek, yadav, aurangzeb, eisner, maher, puri, honeymoon, cartman, tal, woodrow, lindsey, leann, eliza, flynn, durant, netanyahu, asimov, shamrock, keenan, shaun, happili, konstantin, hendrix, namesak, hume, courtier, diva, orson, himmler, welch, becker, yate, cinderella, valeri, mae, zack, naomi, cromwel, andretti, corneliu, sidekick, sanjay, cutler, burnett, teuton, barnard, davey, vick, clapton, brigham, winthrop, khalid, gradi, handel, shapiro, norma, brodi, ritter, connor, bequeath, schneider, hammerstein, collier, samantha, marlon, whitehead, barkley, eyr, yeat, ritchi, mastermind, waugh, rosi, olson, madelein, desmond, alicia, tolstoy, bei, gail, adler, buster, daw, josiah, chandra, abigail, dmitri, shelbi, ramsay, michelangelo, harmonica, schmidt, mcgraw, liszt, wolff, lev, gideon, holliday, finch, gomez, sheldon, englishman, tung, evelyn, magdalen, cowrit, jun, howel, galen, infatu, mckenzi, priya, muller, corsair, lyricist, halfsist, lindbergh, edna, shea, goldberg, germain, streisand, theodosiu, christen, raphael, rutherford, austen, prescott, senna, maclean, alban, uncredit, hain, buckley, bianca, organist, kung, reginald, ramsey, nightingal, macfarlan, boyz, entourag, guo, ripper, sj, peyton, favr, slept, horton, landi, chun, blanch, zachari, timur, cello, offbroadway, barrymor, trey, bain, lu, gough, ping, menzi, gladston, menon, muir, barlow, nguyen, ganesha, murad, adi, cedric, bentley, ing, richter, grayson, pearc, nana, bree, cassandra, wilk, brigg, mullen, varma, helmut, aj, doherti, olli, ruskin, hubbard, moran, dicken, stonewal, nemesi, hershey, stoog, snyder, hendrick, bate, hari, napier, yin, ingram, duff, staffer, protégé, peck, mcdonnel, palin, sergei, nakamura, ja, jenna, hansen, lau, romano, papa, ric, slater, leonid, winger, rockwel, hollyoak, nevil, duan, albrecht, cinematograph, spector, cantor, irwin, gaiu, sweeney, hutchinson, harley, kellogg, choi, dow, nikola, stein, maureen, narayana, sylvest, spielberg, hartman, ander, arya, leah, lucil, siegfri, clemen, geffen, blackwel, tanner, jing, ayer, igor, melani, bartend, jolli, saxophonist, howe, taft, claudia, nat, picard, dobson, carmichael, monti, mulder, carver, duran, grover, flo, moodi, natalia, nathaniel, gabl, brando, kimbal, wainwright, maynard, pj, dunham, alfa, gilli, parton, tendulkar, coowner, baird, blanchard, jang, springsteen, sati, markham, miriam, berat, thierri, rous, hernandez, sharif, patsi, carolyn, anjou, ang, dyer, houghton, pauli, oppenheim, underwood, novella, nader, clarinet, jb, damian, waltz, tennant, cohn, og, mustafa, kemal, saul, beyoncé, omalley, freder, dutt, beaumont, mckinley, minh, greatgrandfath, ayr, gan, malon, oti, tao, mcpherson, rabin, donovan, huffington, agatha, gueststar, cobain, dun, rollin, pir, rae, benton, clau, kyli, karan, gaga, relent, linden, fulton, jj, marcel, cato, tutelag, salvator, orr, compton, canning, ruben, nolan, sila, mcgregor, bernhard, sinatra, chaplin, ao, hector, engel, priestley, gibbon, forsyth, mugab, mcdowel, melinda, pamela, burr, merl, ashton, lawler, virtuoso, ripley, yamamoto, chu, erni, prasad, dalton, paisley, narayan, brutu, cara, housem, arden, dil, vasili, barrow, ala, nicki, bandlead, luciu, hick, cicero, ellison, steiner, hayek, cbe, hubert, reuter, odonnel, compatriot, molest, violinist, andersen, tomlinson, footstep, famer, phoeb, obe, foreword, kuhn, pollard, eusebiu, akira, teller, iqbal, duffi, leela, katharin, kaufman, thorp, iyer, dhabi, bea, shu, ozzi, pickett, gottfri, bender, orléan, carlyl, bono, alexi, göring, fisk, kean, dustin, schumann, lister, cass, oconnor, snl, donni, keegan, ail, benefactor, letterman, kamen, unmarri, darryl, bonham, syke, stefani, sham, madoff, kala, layton, konrad, dixi, yusuf, aur, erich, popper, garrett, merri, philanthropist, mansfield, acharya, rowan, brennan, luka, loretta, jeremiah, tj, cush, darrel, babu, skipper, lacey, hester, kimberli, kazan, bryce, hepburn, mercer, sinha, jovi, graf, asher, burgess, om, fielder, tudor, zelda, lori, zimmerman, greenwood, xu, ballard, terrel, addam, ballerina, putnam, rai, kobayashi, martini, fowler, wiley, brock, alec, massey, kitt, cunningham, julien, loeb, bourn, villeneuv, rubin, slain, squir, gorbachev, bhai, schwarzenegg, harald, mara, persh, romney, simeon, connolli, alf, frazier, rolf, ich, guido, bertrand, doesn, juda, metallica, waitress, foley, spade, mather, oconnel, playbyplay, mohan, abd, cena, hallow, blyth, atkin, tanya, louisa, dolli, surya, zu, yao, gertrud, mandir, rigg, yan, seinfeld, georgi, gareth, chow, inferno, ava, merton, forster, bede, brenda, annett, shakur, larsen, huang, mai, mahler, butch, stefan, skye, smiley, gale, metcalf, ezekiel, bradman, claudiu, hobb, tex, denis, plini, mcguir, dickinson, baxter, vern, mandi, edda, pavel, maximilian, keaton, rhi, chloe, coppola, lillian, liddel, khanna, amar, bachchan, flanagan, jedi, payn, mcqueen, sasha, damon, goldsmith, marian, mccormick, alain, bess, garland, accomplic, émile, caldwel, cosbi, sheen, skinner, eduard, shakti, nair, supper, mosley, raoul, cowork, jamal, burrough, bran, soninlaw, leung, gillard, irvin, megadeth, garth, kendrick, pryor, dandi, majorgener, moffat, booker, derrida, hadley, sheppard, marlow, behest, mariann, nawab, ajay, tweed, beatric, laurent, yd, bhutto, mcgee, phylli, guggenheim, ravi, dirk, curt, wittgenstein, mage, gogh, bliss, allman, prem, derrick, unbeknownst, keller, ching, antoinett, keat, kart, epistl, crockett, ellington, taco, luciano, siva, trudeau, mabel, reuben, boyer, socialit, axel, terenc, hayley, hanna, saviour, eastend, exhusband, holt, barr, baro, babe, dong, larkin, mehta, walton, playmat, scroog, dem, beckham, stravinski, conway, hussain, minni, foreman, peng, matti, godfath, chaucer, rashid, warden, shin, crowley, brewster, messi, fitch, andrei, olsen, knox, simm, frankenstein, barbi, montagu, maa, clayton, lilli, godwin, rees, rosemari, galloway, sweetheart, royc, mori, editorinchief, darci, salim, payton, deborah, kingsley, zia, sharma, brabham, hooper, infuri, ida, flirt, oneal, gillett, unborn, gonzalez, dietrich, nur, ó, mors, finley, sal, waller, higgin, bandmat, foucault, archduk, chand, osullivan, genghi, reddi, roxi, coe, hark, emil, sadler, duma, paddi, edith, slay, addison, jessi, mandela, angi, didn, theresa, leno, duli, devin, clarkson, gerard, perez, jericho, brent, quinci, strauss,

davidson, nilsson, rawl, sheila, nico, ignatiu, nelli, cochran, yuri, musa, yoko, clifford, mackenzi, lola, friedman, johansson, mogul, bose, peacock, gemma, tiberiu, hewitt, mariu, housekeep, haig, jona, hess, mirza, moe, eminem, christensen, tobi, gong, larson, prost, lyle, ike, mahesh, vaughn, luc, drummond, lawson, bloch, yong, eastman, hearst, mansel, kwan, oreilli, brisco, gerrard, frenchman, everett, ariel, kathryn, bauer, sexiest, hemingway, octavian, hilari, zheng, publicist, yun, stewi, manson, ghulam, hoffman, padma, mbe, demetriu, qc, erasmu, graem, wordsworth, viscount, ursula, shackleton, selena, berger, vettel, maud, bogart, boa, barnaba, garfield, abbi, grandmast, murdoch, swann, mcconnel, costello, rascal, tyron, middleton, dina, policeman, kat, berman, tak, sandman, ying, gaston, puja, gina, tess, mobster, hoffmann, caleb, schwartz, cassi, oakley, opin, ji, sculli, manfr, beatti, cheng, dyke, lana, liang, eaton, greer, faraday, spitzer, bowman, mei, timberlak, clifton, arti, hoyt, jensen, schulz, chappel, spock, audrey, bullock, titular, trajan, roberta, buffett, dewey, rana, paig, salman, hathaway, lai, erwin, whitak, tristan, salomon, damien, shepard, crouch, arun, merril, virgil, marcia, ezra, gifford, bismarck, shearer, rosen, wynn, arjuna, alumnu, aubrey, beau, colbi, goeth, patton, mackay, aziz, cleopatra, angu, sutcliff, stringer, stevenson, mukherje, oswald, dai, debra, vijay, eugèn, edi, zoe, ohara, bey, mayer, gama, goodwin, heali, ramakrishna, wren, ness, schultz, faust, spenc, betsi, dahl, olympian, aldin, abe, waldo, jare, gilmor, rowland, hopper, morley, wonderland, dane, albright, stoner, camil, wendel, bene, traver, vinci, fabian, parvati, wilkin, bett, hu, penelop, ahmadinejad, russo, mendelssohn, goebbel, burnham, loren, coolidg, lehman, fleme, distraught, brecht, mein, xiao, maguir, abi, osama, zach, tweet, milli, hindenburg, williamson, nero, lear, josephin, alvin, newel, munro, dominiqu, jock, joachim, bloomberg, warhol, joanna, tagor, kidd, sabrina, saunder, watkin, lowri, tammi, baudelair, nietzsch, hayden, seward, ada, rohan, paleontologist, sherwood, cobb, kai, walther, kara, meek, erica, rudi, holloway, yogi, für, atkinson, henley, yvonn, lara, fiona, deng, haydn, springer, js, merritt, veronica, adel, lerner, joshi, hawthorn, sima, theo, lew, mia, kri, charley, rajiv, samson, juliet, archangel, hayward, meteorologist, weiss, sloan, eileen, alistair, randal, shan, farley, dimitri, hasan, pott, twain, wyatt, mariah, matthia, conqueror, roe, weinstein, hilda, ginsberg, colbert, bakr, heidi, gage, nicholson, overhear, heller, grandpar, marguerit, nugent, jonah, woo, cori, wee, cassel, poirot, esther, alam, willard, durga, hurley, putin, cheney, pollock, leroy, singleton, clint, altman, behead, provost, eulog, mira, norri, raju, dreamer, lakshmi, mir, daley, evangelist, libretto, mitt, betroth, hanuman, barton, maharaj, regina, webber, jasmin, roach, jokingli, katz, nikita, erin, bahadur, mathew, xfile, faber, hippo, lennox, rommel, steinberg, suleiman, jarvi, martel, shen, cavendish, kristen, kaishek, polk, béla, dev, angrili, cho, pooja, viola, bert, marjori, mott, richi, cj, nora, gladi, henson, jayz, simmon, tanaka, dali, anil, wr, gunn, firth, blackston, rep, jasper, capo, cathi, nate, emanuel, gamespot, faulkner, chopin, hodgson, speer, darl, hazrat, alison, grossman, carlton, tchaikovski, mosh, cornerback, shanti, zhao, tarzan, leland, pandit, iain, cheung, hazel, jagger, patel, staci, wharton, groen, viktor, yve, monet, mack, wanda, ogden, schubert, elijah, harriet, chung, garri, keyn, cari, libbi, macbeth, aga, cheryl, lieberman, phelp, vivian, fairbank, talbot, abel, apologis, lucia, peirc, alexei, mclean, kimmel, smyth, copeland, coward, lar, tha, bradshaw, roommat, savil, lena, cowel, dempsey, earp, wilkinson, def, chopra, marr, averi, carmin, ethel, hon, jude, ledger, hotspur, kristin, hua, daphn, tobia, tian, stow, suzi, jiang, templar, mortim, sutherland, bariton, bergman, sai, hatfield, hartley, janic, gao, denton, bradburi, morrow, shivaji, jameson, carli, wilcox, amelia, ricci, mcnamara, pell, conn, ono, arlen, ismail, himach, ej, tam, morrissey, feng, housewif, pundit, exwif, heme, sahib, matilda, gallagh, scorses, cha, jani, harrington, brig, decker, yeltsin, lauri, percussionist, haa, townsend, olga, soloist, ist, sach, gershwin, grime, gardin, sawyer, malik, lim, bret, jodi, hamid, gotti, dori, thom, dreyfu, francesca, amir, waiter, barbarossa, bunt, cindi, keyboardist, lizzi, tracey, siegel, cy, marta, sabin, botanist, amadeu, macleod, orwel, stanton, grandchildren, xv, kahn, kelley, easton, felic, frasier, macmillan, gile, nikki, wasn, nanni, conni, umar, müller, hahn, dickson, barron, shankar, gopal, jimi, estrang, sonia, rankin, elliot, jarrett, headmast, amo, fonda, lamar, megan, middleag, novak, ambros, vinni, marley, nehru, jeanbaptist, deathb, prodigi, maxi, getti, dyson, macpherson, cocreat, druri, stepfath, swanson, harman, psycho, shelton, pasha, greenberg, dawkin, ganesh, tycoon, mona, henchmen, douglass, rasmussen, herbi, judd, countess, diaz, whistler, ling, comedydrama, yue, msnbc, dillon, gillian, huxley, ren, ree, haley, duval, mccall, bunni, jahan, fallon, bowen, rusti, qi, iren, horrifi, jung, rudd, codi, goldstein, minaj, natasha, bartholomew, kemp, dora, whitman, biden, swore, asha, bragg, knowl, radcliff, xian, lydia, granni, mimi, wen, dunbar, jermain, rosenberg, hem, darbi, archibald, prakash, calhoun, fay, uthman, elia, blain, regi, dumont, nadia, gretzki, mckay, enoch, petra, edmond, isaiah, congratul, winslow, aquitain, mister, burrel, clanci, rooney, carlson, clare, jax, sammi, gillespi, chrétien, rufu, gwen, deva, erickson, faisal, vader, kipl, jc, gleason, banjo, amr, billionair, suzann, yew, jeann, bower, hutton, graci, dole, weinberg, aquina, barnett, corbett, mandolin, scotti, glover, saraswati, lenni, feldman, earldom, falk, corey, heiress, gavin, mclaughlin, pai, marlen, xviii, huston, rihanna, werner, mitch, fran,

$\mathcal{V}_3 =$ predic, ellipt, ganglion, gpu, stockpil, taller, asynchron, edema, unpleas, circumfer, ultrasound, placenta, interlock, nanotechnolog, highqual, planar, gestat, blocker, pentium, glide, myocardi, disinfect, modulu, polygon, gnu, fingerprint, plutonium, lubric, conspicu, overdos, uptak, gaussian, vastli, seismic, sedimentari, decompress, truncat, conif, agonist, plum, rotari, xy, cleaner, blister, sq, silt, deterg, alkaloid, broth, jelli, boson, minimis, lettuc, circuitri, fasten, norepinephrin, leach, conduit, barley, subspac, aft, herbivor, testicl, sinu, mismatch, carbohydr, phosphat, lymphocyt, sewag, paralysi, coolant, modular, dementia, hivaid, actin, monom, perpendicular, tangent, longitudin, asphalt, trough, dung, lid, superconduct, fractal, swollen, phosphoru, blackberri, cantilev, disconnect, enamel, discomfort, foam, hydrid, lifecycl, parametr, cretac, smelt, mustard, benzodiazepin, valuat, heater, nicotin, sync, entropi, seam, igneou, avian, airspe, retrofit, coli, shrink, slit, pdf, refract, electromechan, hydroxyl, unintend, starch, longrang, uneven, pineappl, phosphor, tray, quantifi, permeabl, incis, basalt, facet, pancrea, $\leq$, phenol, thruster, graft, lisp, permut, biomass, silic, unman, neurosci, carburetor, incendiari, nonhuman, mussel, inward, recharg, lowcost, venou, pediatr, trajectori, ct, inlet, micro, taxabl, dilat, compressor, cytokin, pvc, masonri, shoal, optimum, grower, highend, lagrangian, granul, predetermin, cranial, inerti, neurotransmitt, inhibitori, alkyl, bowel, gum, pesticid, nitrat, pickl, stamina, headlight, eukaryot, fascia, cytoplasm, verif, porou, pendulum, lupu, retent, hubbl, lumbar, δ, creep, halogen, torsion, recombin, evergreen, adhes, nt, knit, meteorit, methamphetamin, selfesteem, steroid, stellar, harden, coke, subsist, unicod, φ, voip, cellulos, congenit, sweeten, covent, taxonomi, abdomen, manur, fructos, measl, depreci, rodent, firmwar, gsm, pancreat, burner, herbal, yaw, aftermarket, improp, pacemak, hue, handheld, hallucin, intracellular, cmo, alga, slug, mucu, crank, cramp, inflow, megawatt, submachin, hemp, cereal, prognosi, refractori, zoom, subsurfac, insomnia, adob, reflector, cholera, snout, vortex, xenon, platelet, woven, penicillin, fermi, fungi, rust, muddi, graze, polymeras, duplex, evenli, garbag, fungu, tau, horsepow, forehead, tradeoff, sine, debug, flammabl, amnesia, buckl, decidu, nonzero, convect, clog, sulphur, prefront, contraind, finer, syring, peat, atrophi, incub, reload, baggag, auditori, spectromet, lactat, mucosa, sulfid, inorgan, calori, parabol, aircondit, hepat, bruis, decomposit, metamorph, spong, khz, pollin, notch, lichen, mtdna, scalp, null, peni, bleach, lifespan, uv, scarciti, wastewat, filesystem, latenc, thicker, countermeasur, phenotyp, foliag, router, rippl, trivial, soak, psychot, braid, itch, kinas, neonat, ipv, canin, morphin, constrict, amp, bloodstream, airbag, phonolog, inductor, deflat, primordi, macroscop, pastur, matric, proportion, lumber, yeast, pv, spleen, rot, thorac, undul, reddish, gait, microprocessor, dorsal, acet, infus, locu, ballast, tuna, benign, analges, köppen, multiplex, analogu, hotter, gravi, nozzl, tomographi, loosen, morbid, coval, malwar, anomal, quotient, ultrason, hilbert, regimen, pollen, resin, insensit, wifi, workstat, appetit, onboard, beryllium, disson, precaut, infest, inert, builtin, logarithm, grind, eeg, leukemia, reusabl, millet, camshaft, inhal, lighten, pellet, perfor, phylogenet, encapsul, manmad, withstand, cdma, nausea, flex, sap, smallscal, snowfal, covari, tentacl, asymmetri, pandem, crosssect, coagul, vagin, cinnamon, mole, ach, chill, bottleneck, firewal, boolean, pcr, tread, antidepress, firstord, tecton, formaldehyd, lithium, hn, maiz, refil, acetylcholin, taper, subtyp, perenni, chimpanze, inexpens, psychosi,

siphon, refresh, highperform, ditch, anesthesia, particul, breadth, sharpen, microbi, ribosom, litter, strawberri, hf, scalar, url, lifethreaten, ounc, acryl, bait, centrifug, quench, perfum, chimney, pci, baselin, powerpc, dilut, stabilis, epsilon, inertia, boni, kombat, fetal, unequ, shutter, plumb, nucleotid, rangefind, ammonia, magnesium, neutrino, cough, unsaf, razor, filtrat, protrud, pulley, rectangl, dissect, boron, cylindr, milder, retina, thunderstorm, endogen, welldefin, waterproof, mening, addon, overload, eyelid, salin, pore, quadrat, reentri, halflif, traction, undesir, drawback, latex, vertebra, socket, scuba, diod, opaqu, herbicid, graphit, cervic, starvat, bulki, sedat, isom, ef, cn, aspirin, carcinogen, reclam, apoptosi, carnivor, stainless, crankshaft, cholesterol, beak, euclidean, fuze, ovul, twostrok, wedg, geotherm, fluoresc, polymer, waist, mediums, sutur, testosteron, proteas, spp, stool, helix, tremor, oven, backbon, aldehyd, glutam, gearbox, gaseou, gastrointestin, apertur, amphibian, metabolit, audibl, flake, calculu, fiberglass, tint, tether, unsuit, bodili, adren, prune, mould, prenat, crosslink, brine, serotonin, assay, accret, deactiv, pharmacolog, chalk, turbul, elicit, wafer, xml, plankton, euler, termit, antimicrobi, prokaryot, theta, otter, diarrhea, tonic, heterogen, ligament, allel, σ, chromatographi, valenc, headlamp, oyster, ether, mgkg, ioniz, bedrock, taxonom, fibrosi, mammalian, prosthet, crt, noisi, shortest, farmland, parallax, electrochem, gel, ejacul, buoyanc, bmp, seawe, doppler, pelvic, axial, tensil, welldevelop, allergi, arabl, lumen, telephoni, flap, dredg, intox, amd, scaffold, plume, atm, deforest, lc, dipol, tungsten, excis, monoton, hydroelectr, scanner, tightli, rash, bladder, nebula, applianc, seafood, gut, css, plumag, viscos, clariti, λ, bulg, anemia, deceler, antioxid, citru, dehydr, purifi, scrub, amplitud, kbit, chipset, homolog, gunpowd, octav, lessen, costeffect, asymptot, millisecond, footprint, hypothet, cumul, amphetamin, pariet, extinguish, resistor, supercharg, spectral, binomi, opioid, dashboard, viabil, vesicl, benzen, lcd, landfil, buildup, aerosol, barb, allerg, pneumat, spectra, urinari, grassland, tannin, numb, inflect, macrophag, capacitor, cleav, emuls, tcp, mitochondri, monoxid, photosynthesi, placebo, topographi, counteract, intermitt, fission, petal, etiolog, tyrosin, beet, cathet, lug, ghz, inciner, muscular, excret, toxin, ampl, subunit, syphili, tick, damper, safer, duct, endocrin, sweat, steril, epidemiolog, glue, embryon, leakag, mesh, transluc, dosag, methanol, pear, attenu, palett, convolut, latent, semiautomat, shunt, cleavag, fluorid, reactant, continuum, obliqu, hamiltonian, wearer, ht, ellips, errat, ev, hydrolog, dough, centimetr, µm, asthma, biopsi, inflammatori, electrolyt, amplif, spoiler, sausag, regen, interperson, cobalt, timer, lath, diffract, lowlevel, fece, highpressur, subduct, washer, impart, munit, handset, autoimmun, powertrain, plywood, vagina, salti, malnutrit, halid, asbesto, microorgan, submerg, relativist, necrosi, debilit, hygien, throughput, manganes, unload, magnifi, smallpox, perturb, lymphoma, iq, avion, π, saliva, pasta, probabilist, schrödinger, recurs, methan, hairi, sac, virul, slab, outpati, mangrov, glaze, retard, tactil, tonal, ventral, smoother, pastri, waveform, thermomet, ultraviolet, hotspot, transmembran, cuff, unix, fungal, kwh, triangular, scrape, fixat, anaerob, walnut, isomorph, charcoal, seawat, legum, movabl, exacerb, toplevel, cocoa, contour, appendag, breech, vomit, qualit, pavement, stew, splice, realworld, agil, clad, drier, dataset, cortic, pars, epithelium, seab, impur, immatur, serum, dim, shred, atyp, hydrat, hydropow, gust, thyroid, stochast, urea, ovarian, uteru, medial, endotheli, bacterium, damp, decompos, prism, synaps, primer, thirdparti, mri, scalabl, selfpropel, ganglia, javascript, lorentz, brood, usabl, airflow, coars, sticki, orthogon, germ, polyethylen, opportunist, ester, gastric, yearround, hydrolysi, calibr, reset, zip, ionic, convex, slender, chew, migrain, pebbl, curvatur, groundwat, snack, peptid, rainwat, outweigh, potent, contigu, nmr, harmless, cadmium, brittl, modem, postag, nectar, clade, anal, follicl, generalpurpos, stove, vend, cathod, yogurt, contracept, capacit, ozon, kappa, indistinguish, milki, neon, cation, thicken, theropod, solidst, uterin, howitz, arthriti, electrostat, nylon, ε, dissoci, localis, anatom, celsiu, softer, thinner, warhead, hash, dendrit, rectifi, tubular, dimer, ulcer, anesthet, viscou, linearli, highenergi, smoker, hemoglobin, lobster, warmth, carbid, biodivers, occlus, vanilla, diaphragm, α, microscopi, tar, anod, hover, sideeffect, seedl, brighter, thereof, aircool, cdc, heaviest, topograph, vitro, folder, innerv, ripen, reagent, ailment, eucalyptu, cornea, supernova, illicit, immunolog, quark, nuclei, queue, edibl, mildli, quantiz, concav, innat, bipolar, determinist, cigar, onsit, aromat, smartphon, polymorph, titanium, slr, actuat, interconnect, mixer, outward, centimet, pylon, dohc, zx, resili, riemann, hydro, loader, asexu, lamin, windshield, floral, weakli, latch, emitt, garlic, peroxid, tricycl, ovari, lng, antipsychot, intric, subfamili, compost, bile, bulb, abras, mitochondria, redirect, synapt, tandem, histolog, ventricular, gelatin, olfactori, hippocampu, histon, mpa, transvers, cabbag, alkalin, flang, filament, shrimp, polio, torso, ammonium, condition, β, raft, unaffect, nematod, asymmetr, laundri, swine, denser, planck, cartesian, infinitesim, mach, endpoint, intraven, necessit, ccd, cushion, helium, mimic, exce, maneuver, arthropod, readabl, cyanid, booster, chloroplast, aneurysm, saltwat, chemotherapi, unpredict, petrochem, cutoff, mandibl, carcass, alveolar, fixedw, ppm, microb, fore, fig, kv, hyperbol, increment, θ, transistor, suction, situ, perceptu, tumour, impract, carboxyl, truss, μm, fume, fertilis, spacetim, semiarid, ineffici, etch, incandesc, squeez, alluvi, interoper, buttock, motil, runoff, aquif, brightli, pelvi, sew, tighten, lag, greas, lymph, nucleophil, binder, projector, odor, conic, vinegar, aroma, dirac, enzymat, colorless, projectil, bhp, darken, poultri, drip, embryo, bromid, ligand, shrub, ieee, symptomat, scent, pcb, aortic, germin, biodiesel, eigenvalu, manpow, chromat, lh, fern, millimet, collater, funnel, homemad, toxicolog, malign, crystallin, mango, $\pm$, brows, irrevers, biochem, migratori, spindl, coaxial, disproportion, semen, distal, moist, constrain, predatori, fibrou, frontal, loaf, syntact, respir, luggag, strata, mosquito, hierarch, warmer, immobil, elast, sturdi, camouflag, kiln, forearm, invertebr, offroad, iodin, lexic, hydrophob, spectroscopi, rom, chlorin, spheric, dimorph, feeder, byproduct, thigh, schema, psi, mbit, faulti, cytochrom, orgasm, isoform, autosom, biotechnolog, highpow, chromium, sprout, dopamin, infarct, basal, flatten, scsi, vat, mpeg, cucumb, rub, lumin, irradi, markov, aberr, insolubl, aquacultur, granular, plasmid, coronari, nomenclatur, ripe, fissur, wheelbas, foodstuff, biofuel, cleanup, pathophysiolog, handgun, apic, relaps, planetari, corneal, sulfat, sheath, hexagon, quadrant, halv, cyclic, menstrual, cataract, sonar, influenza, yarn, conveyor, shingl, malfunct, yellowish, barium, inlin, jpeg, methyl, genotyp, luminos, nonstandard, bayesian, carbonyl, outflow, androgen, glu, overh, loudspeak, lipid, antisoci, poisson, suppressor, â, shellfish, condom, replenish, fetu, ipad, clot, postur, fourier, superfici, bsd, savanna, lengthen, rudder, lump, carrot, recoil, magnif, encas, catalyt, iodid, taxa, fluorin, preferenti, suck, hamstr, avers, massproduc, hydroxid, weed, hz, capillari, pallet, sanitari, lattic, epitheli, soybean, markedli, anion, ic, imbal, carcinoma, ecm, overlay, carbin, cryptograph, helic, caffein, microwav, alkali, sore, tab, higg, heurist, purif, clamp, plaster, lightli, transloc, http, anomali, twodimension, extracellular, estrogen, malt, linen, geodes, workload, mainfram, indent, firepow, threedimension, scaveng, touchscreen, collagen, pariti, radiant, antiinflammatori, cryogen, hose, kerosen, subtract, nucleic, squid, clam, subclass, nitric, opensourc, pituitari, insecticid, flu, motherboard, macintosh, metadata, ubiquit, γ, glycol, palsi, abstin, canopi, retrograd, colder, affix, airfram, vivo, sickl, dehydrogenas, chunk, potenc, html, predictor, adhd, epilepsi, rgb, canist, phylogeni, linkag, cassava, radial, hardwood, nostril, hemorrhag, ruptur, biosynthesi, aqueou, nodul, flare, pounder, dn, metallurgi, volt, cipher, biochemistri, mrna, complementari, ω, crustacean, nippl, solder, wingspan, strut, microbiolog, almond, intrus, vapour, extratrop, magma, mite, spore, fahrenheit, diurnal, soy, forag, uncontrol, cleft, glacial, vertex, disloc, lemur, marrow, bluetooth, photovolta, unwant, transfus, droplet, sludg, retin, fp, sewer, molar, cartilag, sql, galvan, delic, agar, instantan, powerpl, molten, sunflow, subsystem, palat, thorium, untreat, quicker, punctur, transient, uniformli, spici, soften, soda, gui, ethylen, polyest, vascular, μ, axon, petrol, somat, superstructur, functor, precess, turbocharg, hydrocarbon, syrup, oneway, sugarcan, tendon, knob, dryer, furnac, hyperact, kb, arid, benchmark, streamlin, pouch, skew, minu, runtim, stationari, macro, fourcylind, apex, cystic, phosphoryl, overflow, infertil, dope, sclerosi, chiral, interstellar, sequenti, tuber, hing, filler, repositori, undersid, ethyl, lambda, transduc, sn, nocturn, nanoparticl, skelet, rearrang, amorph, spam, keyword, inactiv, ethernet, caterpillar, bog, longitud, massag, bio, snail, throttl, ventricl, radiolog, elong, quartz, malform, aerob, weaponri, hypertens, groin, ipod, concuss, redistribut, summat, rivet, cultivar, pheromon, takeoff, catalyz, dielectr, silica, floppi, paddl, amput, rf, mollusc, keton, cyst,

$\mathcal{V}_4 =$ anchorag, uci, bremen, metropoli, holstein, terrier, sagar, airstrip, decommiss, rink, pisa, burgundi, showdown, wichita, raleigh, honolulu, playhous, hillsborough, essen, openair, yucatán, sooner, careerhigh, cheltenham, augusta, bazaar, suntim, avon, internazional, regatta, awa, luzon, taekwondo, sw, tahiti, hereford, galatasaray, wat, punic, wyom, swindon, stirl, samoa, surrey, boardwalk, goaltend, lynx, zurich, midwest, cypress, hackney, fruition, lineman, pendleton, hampstead, pike, sinai, warwick, paralymp, britannia, lowli, tripoli, eskimo, qs, hom, vicechancellor, durham, chengdu, triest, lsu, barangay, somerset, hermitag, dakar, payperview, baja, metz, silesian, williamsburg, antigua, galway, fillmor, kochi, heathrow, patna, lauderdal, grizzli, jamestown, swat, chattanooga, equestrian, chesapeak, hilton, farmhous, headtohead, arcadia, heidelberg, genoa, sofia, suffolk, dorset, borneo, berkshir, racetrack, fk, tallinn, ghent, auditorium, northbound, utc, calai, canuck, centenari, goali, shortstop, limerick, ut, yearend, volga, granada, atol, thenc, blazer, nugget, chinatown, nxt, ipswich, geelong, southward, cologn, auckland, glastonburi, seoul, xm, galveston, thoroughfar, schleswig, ural, reenter, brunel, usl, salford, iaaf, raptor, deco, palermo, tavern, haifa, turf, infield, dresden, georgetown, reschedul, gamewin, northumbria, unbeaten, outskirt, threepoint, cairn, biennial, wolverhampton, aggi, huski, inver, bodybuild, rotunda, fordham, squash, dormitori, encamp, colspan, somm, qanta, redshirt, mcgill, bahama, nagar, caf, humber, monmouth, stoni, uruguayan, kathmandu, ff, wellesley, bathurst, cove, carlisl, tucson, antwerp, tf, upstat, centenni, everglad, disembark, northerli, myrtl, taluk, durban, kolkata, turin, midget, penang, doneg, langley, schoolboy, sacramento, straddl, yokohama, thessaloniki, indu, viaduct, niagara, backtoback, newport, prom, vermont, greenwich, postcod, aleagu, guangdong, subregion, savoy, eal, seasid, olympiad, twotim, mangalor, zürich, nagpur, allamerica, mrt, barbari, northernmost, wta, burbank, argyl, jurass, nj, ventura, freiburg, tmobil, woke, fewest, semi, roh, kennel, madeira, staterun, asean, vicker, siberian, shawne, argonaut, hsbc, precinct, lill, nippon, verona, fresno, monterey, belgrad, jaya, guangzhou, sumatra, redskin, bhopal, redoubt, flint, varanasi, interc, tryout, showtim, norwich, heisman, bogotá, intercollegi, occident, renumb, anfield, undraft, abscbn, saratoga, snowboard, wiltshir, bois, boulder, cheyenn, dealership, starboard, ute, boutiqu, vale, claremont, tonga, canberra, shropshir, arbor, hinterland, tehran, gladiat, salem, seminol, olympia, sept, cavit, nrl, steamship, coldest, minneapoli, edmonton, turnpik, midwestern, anaheim, taipei, sesam, derri, belfast, mersey, entrant, beacon, stuttgart, salzburg, maui, fujian, ohl, ctv, jaipur, grang, savannah, irb, picket, motel, northumberland, callup, upland, rochest, pyongyang, cnbc, fedex, kiel, roadblock, nagoya, luton, colombo, bungalow, gettysburg, horsedrawn, eastwest, loyola, staten, adriat, mariana, vauxhal, powerhous, potsdam, humboldt, raceway, clemson, coimbator, embank, waterfront, caen, camden, fremantl, hillsid, bere, caledonia, ashram, sarajevo, upn, goalscor, indycar, burnley, bergen, bronx, astro, wildcat, repertori, shipyard, doncast, barnsley, hawkey, wakefield, defenceman, aaa, swansea, strikeout, wb, andalusia, hq, début, fulham, annapoli, storey, mma, sichuan, fjord, firstround, knoxvil, kickbox, pagoda, heineken, baden, basel, longdist, promenad, dunde, panhandl, azor, captainci, dortmund, panchayat, exet, siriu, waterford, perth, condor, croydon, pontiac, nanj, gothenburg, regularseason, mekong, sumo, byu, nit, kraków, auburn, islet, ajax, eureka, newark, lisbon, peke, watford, simulcast, zion, himalaya, oiler, barnet, steamboat, nassau, bash, sunda, mainz, godavari, armori, hampton, fai, caledonian, nyc, rté, dor, wilmington, soho, hobart, calgari, aspen, sorti, westchest, coeduc, doha, shutout, galile, alsac, windsor, albani, olympiaco, bordeaux, bsc, oncampu, highestgross, lucknow, airbas, westfield, rotterdam, trolley, transvaal, vanderbilt, regroup, nyu, spruce, jakarta, landfal, superson, oricon, allegheni, disus, saracen, skylin, nautic, westbound, wharf, nl, casablanca, mesa, fiesta, antarctica, hammersmith, louisvil, pacer, wadi, fuji, twentysix, pba, judo, quezon, freshmen, amphitheatr, sevilla, albuquerqu, halfhour, stepp, gloucestershir, avalanch, southampton, raffl, panathinaiko, badminton, dhaka, novi, aegean, lièg, collieri, cfl, dinamo, barclay, cheshir, bali, colchest, wessex, akron, crosscountri, toledo, blackpool, parkland, juventu, augsburg, massif, calcutta, allaround, macau, parma, midatlant, caspian, postworld, eindhoven, hanov, pune, leyt, cayman, wba, marriott, fl, airlift, peripheri, merseysid, meridian, papua, halifax, blitz, cbd, seaport, glendal, kota, cyclist, stalingrad, kingston, malibu, ballpark, outfield, purposebuilt, leipzig, worcest, expo, lufthansa, eri, guantanamo, euphrat, fir, oriol, golfer, copenhagen, barg, amherst, uptown, bison, carpathian, aba, dartmouth, gloucest, peterborough, eastbound, aero, vodafon, voivodeship, leinster, sprawl, smoki, loch, steamer, peninsular, sinojapanes, nave, dunk, caldera, palisad, concordia, secondlargest, diamondback, prep, collingwood, ghat, skier, gala, fiba, loir, wellington, kyoto, sixday, bermuda, golan, valencia, cumberland, southernmost, blackhawk, aerodrom, walkway, fremont, danzig, streetcar, tacoma, midseason, highris, spree, scarborough, oclock, omaha, highschool, timor, allireland, albion, zee, lafayett, matricul, bayer, elgin, wrexham, piccadilli, aqueduct, caraca, homecom, tasmania, caravan, bournemouth, sill, stoppag, dweller, yeshiva, transcontinent, yangtz, timeslot, unc, distilleri, sellout, weeknight, midsumm, avro, poli, whitehal, catchment, sk, cavali, saskatchewan, worcestershir, electrifi, suez, warwickshir, oslo, piedmont, middlesbrough, hilli, belmont, watersh, manitoba, gotham, shetland, antil, selangor, rodeo, hilltop, sabr, bucharest, bethlehem, stockholm, midtown, fb, tundra, aberdeen, severn, potomac, churchyard, pomerania, kany, concours, oceania, norwood, comcast, wildcard, alta, ballroom, pennant, stamford, grandstand, subdistrict, realign, hove, sevil, dockyard, clipper, overtook, firstyear, norfolk, hostel, chatham, pdc, lancast, sioux, dorm, ovat, daytona, semifinalist, baylor, ave, odessa, bastion, bandar, nowdefunct, fairfield, condominium, uninhabit, avalon, johannesburg, faro, euroleagu, benfica, beirut, guam, buckinghamshir, lazio, singleseason, shandong, alaskan, cod, portsmouth, phra, millwal, concord, nairobi, buccan, napoli, utrecht, boca, threetim, moat, jaffa, wbc, bookstor, pretoria, fairfax, eton, monorail, riversid, aurora, uppsala, jutland, spokan, bukit, grenadi, shenzhen, kimberley, lockout, greenfield, platt, greyhound, ipl, himalayan, upscal, skyscrap, shrewsburi, wineri, shorelin, slough, monza, bluff, saigon, helsinki, grassi, warmup, pyrene, unincorpor, arlington, feb, düsseldorf, charleston, overland, waterloo, corinthian, stockton, twentyfour, mannheim, nohitt, penultim, madurai, algier, handbal, borussia, soldout, bobcat, canari, cork, yosemit, mare, coliseum, equatori, snooker, corinth, lexington, bridgehead, yukon, anzac, nw, addi, stratford, zagreb, dover, danub, isthmu, ravin, tiebreak, guernsey, chesterfield, quay, pregam, okinawa, inducte, cumbria, tuft, kandahar, winnipeg, portico, brandenburg, afb, verd, palo, chestnut, guildford, cska, northampton, wesleyan, lookout, argo, downhil, causeway, lans, natal, undisput, stockport, elm, porto, bonn, naia, concacaf, parramatta, coyot, bologna, strasbourg, knick, fargo, italia, tyne, breaker, spitfir, gma, az, badger, falkland, tvb, budapest, harlem, enfield, beaufort, threeway, anglia, devon, dynamo, canadien, oasi, triplea, christchurch, rada, tehsil, raaf, aachen, racecours, carmel, tbilisi, comanch, dayton, nottingham, sunris, rampart, bilbao, yellowston, agra, chichest, gaa, rochdal, marlborough, cougar, snowi, asiapacif, arrondiss, champ, mcc, brighton, psv, purdu, oxfordshir, inelig, constructor, disneyland, maroon, sein, fenway, rutger, pembrok, fia, bromwich, carleton, tko, roundabout, aisl, bedford, eurasia, tx, foothil, usaaf, staffordshir, redwood, riyadh, salisburi, transatlant, citadel, calder, derbyshir, darlington, huntington, deccan, busiest, rerun, tianjin, ahl, kindergarten, mexicanamerican, cheerlead, essex, gmbh, outli, harrow, trafalgar, allstat, amtrak, charlton, woodstock, elb, pasadena, pullman, amman, yunnan, professorship, scoreless, courthous, marquett, burlington, everest, hertfordshir, toulous, munster, ecoregion, kensington, ymca, sv, jfk, nant, bye, marlin, andean, cdp, bloomfield, malmö, havana, allahabad, confluenc, málaga, tulsa, nsw, escarp, buena, sussex, circumnavig, reno, tramway, ganga, bakeri, topten, stamped, chittagong, montevideo, agglomer, standout, hartford, buckey, coventri, crossroad, clubhous, bangkok, westwood, idaho, surat, appalachian, lima, multipurpos, firstev, leiden, southbound, middlesex, nouveau, bari, glee, picturesqu, siemen, phnom, waiv, tuni, gymnasium, oldham, shire, kremlin, australasia, lago, westernmost, syracus, hanoi, cebu, lincolnshir, tyrol, paddington, tee, mercia, whaler, welterweight, northsouth, rooster, hc, trenton, penitentiari, lausann, longhorn, huddersfield, ahmedabad, tuscani, guadalcan, slum, zoolog, marseil, cafeteria, orkney, wnba, mainlin, lighthous, kilkenni, roanok, osaka, sidewalk, lancashir, macquari, ashor, rerout, mindanao, feyenoord, hurl, homestead, brunswick, trafford, sahara, alto, württemberg, raze, equalis, puma, fife, ecw, plymouth, seneca, mohawk, huron, porch, oneday, stagecoach, govt, matchup, scrimmag,

$\mathcal{V}_5 =$ vain, misconduct, glider, practis, solitari, traitor, vengeanc, vanish, pertain, safeguard, remembr, subdu, axiom, salut, nobodi, bump, judgement, gentl, deaf, restart, runaway, eighti, smuggl, heroic, sneak, trooper, timelin, smile, bulli, exemplifi, allud, sheer, bb, thiev, breeder, immers, bp, veto, noon, sympathi, realism, lifelong, phantom, thor, cosmolog, karat, sermon, tailor, hesit, anecdot, ch, wive, rational, liken, defi, straightforward, deadli, reincarn, perpetr, risen, creditor, curb, achil, imper, multimedia, token, vandal, penni, sb, tremend, dilemma, rhyme, terribl, spoof, bail, loot, forgiv, redempt, arbitr, lan, banish, spartan, repuls, conjectur, genealog, pill, blur, semin, donkey, ci, awaken, regret, veil, taxpay, landslid, ark, bizarr, newest, ge, exagger, pentagon, pal, hr, groom, inhous, masculin, atroc, wholesal, midst, entireti, distract, unexpectedli, purg, embarrass, graffiti, isnt, homag, undertook, revert, onscreen, rand, prop, acquit, stab, mess, summon, slayer, pre, nonexist, jade, contempt, dissemin, twothird, reallif, cannib, racist, bred, clown, probat, caution, wick, brilliant, ll, nude, evok, firework, roam, inact, messeng, gotten, weird, rm, revisit, theyr, somebodi, incap, hallmark, unwil, tori, ineffect, homeless, calm, pretend, bias, diversifi, extant, problemat, unpreced, ts, wouldnt, motto, allot, transcend, pragmat, sincer, remodel, spectacular, guilt, funni, cheat, obey, undercov, banknot, flock, notifi, quotat, somehow, vener, repent, colloqui, mayb, auster, mega, anymor, grate, dalek, noway, sharpli, trump, annihil, epa, amin, ware, unto, novelti, melod, atari, wield, invis, courag, saloon, atheist, onethird, decim, surprisingli, disastr, starv, swear, eager, countdown, inadvert, outrag, belov, cosmo, disclosur, ki, dividend, highprofil, agit, disapprov, promptli, talmud, await, halo, proxi, justif, inmat, onair, astonish, deduc, drunk, cp, parcel, thrill, reassign, dull, satan, feloni, plausibl, mural, ec, eccentr, espionag, sensibl, transgend, yell, twentyf, yahoo, rever, unfair, unus, wherein, connot, eras, deserv, erad, storytel, coven, couldnt, sabotag, optimist, taboo, restless, ve, ni, elderli, vow, shame, supposedli, intuit, countless, jealou, sunshin, inappropri, nurseri, sorri, rage, hamper, slaughter, pharaoh, samurai, psychic, comed, hadith, plaintiff, katrina, clue, fcc, scarc, firstli, paus, thou, excerpt, incompet, unaccept, demolit, thoroughli, hijack, tomorrow, annoy, bisexu, marketplac, faint, misunderstand, burger, . . . , profici, erot, spotlight, gunner, indulg, cr, transcrib, secreci, stir, avatar, dear, humbl, signifi, psychedel, salon, feat, lure, secretli, insult, curios, newborn, infin, peculiar, worthi, fx, suspici, rhythmic, sexi, wherev, ascent, ka, startup, endeavour, excus, flashback, viciou, foe, propheci, chronolog, mistaken, gag, sacrif, fundrais, hello, injustic, popularli, treason, blunt, revamp, immort, stakehold, bloodi, clever, awkward, liabl, puriti, whisper, compliment, £m, reconcili, imperson, heighten, unpopular, pascal, zeu, oversaw, supersed, ridicul, drone, plc, skateboard, abid, mock, werent, denial, yoga, famous, seldom, socrat, slash, est, rc, disposit, em, gi, motown, ugli, stylist, disqualifi, weaker, charm, ari, metaphys, makeup, messiah, dell, es, wont, allah, commend, societ, triad, endless, metacrit, thwart, rpg, au, thief, ra, serpent, decisionmak, disintegr, mitsubishi, whoever, viewpoint, receipt, seduc, credenti, merci, cruel, subsid, indict, afflict, ambit, altogeth, occult, frighten, roleplay, ah, scrutini, spars, va, mistakenli, highlevel, ai, adulthood, workforc, bust, fanci, contractu, hadnt, arguabl, overturn, skater, exposit, broaden, foremost, ambiti, arent, dec, incorrectli, gentlemen, interf, banquet, scarlet, porn, abduct, amid, imperfect, batch, slim, acronym, obscen, bunch, soror, perish, plato, notori, everybodi, blackmail, fist, brothel, satisfact, torah, perfectli, ae, meantim, si, chariot, nc, prehistor, buffi, landlord, forthcom, amc, persona, insan, bounti, deceiv, fiat, verdict, various, amidst, evad, tran, pleasant, reappear, lucki, reintroduc, prelud, archetyp, discern, enumer, marg, hercul, rework, afraid, lefthand, loser, quietli, menac, fresco, lgbt, crippl, unnam, criterion, coma, stupid, oneself, bestow, interrog, youv, strive, gestur, weve, beneficiari, proud, holocaust, paradox, sporad, amass, neglig, postmodern, arrog, ransom, hinder, hack, devote, loud, marit, persuas, nice, inflict, bedroom, noteworthi, daddi, terrifi, compass, hungri, decept, femin, impend, adversari, suspicion, litig, destini, unreli, scare, harder, medicar, evidenc, chiefli, freak, lunch, repel, robberi, stipul, fragil, entail, amnesti, fraudul, ruthless, alik, hatr, mod, reclaim, freud, mourn, vigil, asleep, shout, reconcil, babylon, gangster, kant, importantli, rumour, therapist, demis, orphan, riski, despair, bt, shi, drunken, sad, jealousi, bowler, python, geniu, obvious, daylight, password, aforement, sub, indefinit, standalon, resent, aristotl, undergon, casual, handicap, implicit, injunct, urgent, goodby, simplic, keeper, attest, intimid, nonsens, pend, unconstitut, wipe, quarrel, rfc, guess, swimmer, jehovah, outlook, millionair, useless, tolkien, righthand, vintag, negat, hisher, disgust, tsunami, umpir, skeptic, provoc, bargain, mythic, rewrit, pivot, relic, unconsci, eyewit, rid, backdrop, atlanti, intensifi, dislik, clarifi, apocalyps, humili, dispens, interpol, noir, sacrament, erron, turnov, sp, masterpiec, aggrav, seventi, prematur, halloween, cremat, bearer, incit, parol, versatil, akin, retrospect, opium, climax, discrep, quota, willing, broker, vest, enrag, fond, haunt, samsung, vagu, magnific, psalm, grasp, digniti, ng, dracula, wane, sympathet, overli, nowher, ostens, allus, slang, ufo, homicid, formid, boycott, instantli, intim, xmen, karma, comrad, unawar, conscienc, slap, detriment, offspr, energet, astrolog, anyway, havent, asylum, repay, lucr, purana, obsolet, hardship, setback, incident, certainti, voluntarili, bold, unlaw, ar, plead, reminisc, resurg, memorandum, hereditari, deceas, hare, customari, myself, nineteen, confisc, mom, ok, cocacola, mileston, vivid, overnight, isi, fabl, tragic, obedi, autobiograph, magician, rude, dad, circumcis, narcot, min, leverag, unfold, foul, hoax, decent, preexist, ought, honest, obsess, wise, dissent, invok, drown, inscrib, postul, ex, vigor, irrelev, infiltr, ninja, nonstop, discredit, famin, brutal, imaginari, sting, lender, characteris, fascin, memo, adolesc, temptat, plea, ultra, luck, cs, underway, bodyguard, ourselv, onstag, bankrupt, ir, muse, defer, breakup, salvag, shooter, mao, relianc, refut, incarcer, salvat, lengthi, firmli, envis, rogu, redeem, slogan, shatter, ea, reluct, everywher, eleg, underworld, theoriz, succumb, misus, immin, celesti, blockbust, ancestr, notorieti, damn, bandit, re, utilis, learner, covert, nineti, costli, sage, undisclos, outright, coincident, cruelti, hedg, usher, hed, dharma, mighti, cohes, mankind, allegi, unfinish, mutant, gon, anybodi, rode, spous, yearli, pawn, gentleman, rubi, retali, furnish, caller, gypsi, sorrow, panic, absurd, finest, nonviol, inaccur, secondli, unavail, seeker, unlock, confidenti, stranger, doom, hound, forbid, wan, farewel, reel, instinct, mislead, outspoken, invalid, purport, ape, hardli, summar, yourself, shaman, stanza, oracl, sake, dare, heterosexu, lament, tempo, forcibl, tu, vegetarian, phonet, buzz, uncov, incred, outlaw, furiou, maya, musket, ego, idl, iri, pornographi, dismay, sd, delight, curiou, unlimit, deed, baptism, crook, ti, indiffer, censor, archaic, gossip, relaunch, heavenli, empow, corps, ptolemi, poetic, wellb, ascrib, abruptli, codenam, restrain, inquir, randomli, cobra, hint, intoler, shelv, cop, batsman, keen, handsom, stuff, constel, restraint, bribe, fool, mob, ador, marijuana, commodor, prejudic, endeavor, wrath, reinstat, longstand, emphasis, prestig, tb, enthusiasm, miracl, horribl, grief, bother, stun, lent, merced, coexist, feminin, stole, corpu, refrain, illus, oh, contempl, skip, umbrella, fuck, hunger, authoris, pardon, incompat, intrigu, libel, gym, forgotten, soar, audi, verg, crise, supernatur, porsch, par, aint, pray, humour, suzuki, utter, paragraph, deter, censorship, firefight, disregard, prefac, nightmar, exodu, unexpect, ko, tempera, uncomfort, pointer, forget, conspir, amen, furi, unhappi, evict, beg, ya, swap, coffin, thorough, recogniz, solicit, overhaul, hobbi, aw, spoil, reiter, infam, stunt, ta, prank, disguis,

$\mathcal{V}_6 =$ cartel, guerilla, huntergather, nazism, sparta, stronghold, baptiz, cleans, sloven, planner, detaine, quo, bahá'í, jat, kurdish, unpaid, parthian, swede, orphanag, authoritarian, colonis, gestapo, extermin, moravian, militar, abolit, sicilian, unicef, annal, assyria, nepali, signatori, hama, safavid, royalist, academia, reorganis, cornish, islamabad, sabah, acced, seleucid, chaplain, xiongnu, alqaeda, yoruba, anatolia, demarc, grassroot, vehement, nagasaki, expeditionari, abolitionist, cree, epiru, aleppo, nkvd, indoaryan, plunder, kazakh, hispania, nationalis, détat, vicar, upbring, breakaway, ghanaian, mesopotamian, angloamerican, suffrag, anarch, bicamer, bourgeoi, goodwil, aristocraci, gentri, rajasthan, iroquoi, priori, orthodoxi, auditor, paratroop, kgb, malacca, amerindian, galician, extremist, charismat, milošević, joseon, automak, rwandan, paraguay, curia, huguenot, xinjiang, oriya, uzbekistan, wehrmacht, chechnya, bantu, chairperson, celt, offshoot, montenegrin, dissid, zulu, hezbollah, mesoamerican, unrest, samaritan, maori, lakota, fianna, entrench, druze, eucharist, scandinavia, privatis, parliamentarian, frisian, prerog, gupta, mayan, uncondit, johor, hannib, austriahungari, gibraltar, freedmen, downfal, extradit, insular, codifi, arafat, protestant, burgundian, tasmanian, rhineland, carthag, transylvania, silla, caretak, ankara, franciscan, brethren, herodotu, palatin, guiana,

truce, yiddish, confucian, mestizo, francophon, intergovernment, hegemoni, enshrin, senatori, somali, naacp, hutu, nizam, eurasian, liberia, peerag, oman, imf, antioch, politburo, staunch, kashmiri, iit, espous, peso, akkadian, arian, uzbek, bedouin, oust, algerian, banu, aeronaut, estonian, gnostic, junta, outnumb, jiujitsu, seced, auschwitz, frankish, germanspeak, guyana, namibia, conven, esoter, puritan, pinyin, unitarian, briton, overthrew, bureaucrat, dacia, basra, crackdown, precolumbian, overrul, azerbaijani, hittit, heraldri, meiji, modernis, priesthood, mutini, kuomintang, launder, outreach, seljuk, aryan, romani, transliter, meteorolog, caucu, assad, heret, tantric, jurisprud, rescind, sumerian, ascens, bohemian, claimant, barbarian, athenian, pretext, apartheid, secess, turmoil, sinhales, multiparti, privi, hakka, strife, achaemenid, weimar, lahor, bolshevik, gallic, benin, appeas, elizabethan, acadian, canaan, gentil, sizeabl, habsburg, seneg, creol, defam, carthaginian, moldavia, burgh, unilater, ombudsman, sukarno, maltes, recaptur, bohemia, burmes, masjid, ngo, regenc, capitul, edo, peacekeep, cadr, singaporean, pali, demot, tunisian, preemin, edict, monast, manchu, czechoslovak, catalonia, diplomaci, englishspeak, rhodesia, ensign, spaniard, knesset, defianc, intermarriag, schism, unoppos, subjug, hagu, andalusian, policemen, nordic, subcommitte, goth, barbado, belarusian, fledgl, consular, majesti, seafar, croat, coinag, noncommiss, iberia, malawi, khmer, sharia, haryana, servicemen, ceylon, archeolog, eunuch, selfgovern, antislaveri, subcontin, counterterror, bihar, artisan, riviera, plight, ceasefir, catalan, roug, malaya, hardlin, devout, zen, ultimatum, mistreat, odisha, dragoon, airmen, nobleman, turkmenistan, solidar, bylaw, slovenia, kabul, dalit, paraguayan, warlord, inuit, darfur, tort, imposit, zoroastrian, rector, bosnian, serf, rupe, seventhday, instig, bavaria, gop, expatri, rumbl, entrust, iconographi, niger, dynast, jacobit, excommun, taoist, unitari, babylonian, benevol, ru, lawmak, latvian, gaddafi, notari, paleolith, censur, khyber, historiographi, austrohungarian, marxism, hokkien, tyranni, postsecondari, populist, fishermen, bolster, bhutan, nuremberg, sarawak, constitution, liturg, reformist, subsaharan, cornerston, individualist, sui, semit, travancor, mamluk, somalia, fiji, taiwanes, byzantium, baroni, separatist, zambia, disarm, ravag, zanzibar, kazakhstan, hillari, multin, civilis, devolv, chalukya, leningrad, roc, seamen, mongolian, nonchristian, heresi, kali, sindh, lowincom, hajj, gubernatori, nicaragua, vilniu, quaker, grievanc, uyghur, swahili, assent, mujahideen, postcoloni, quell, prefect, wealthiest, patrician, mon, belaru, igbo, suharto, tripartit, grenada, incurs, spearhead, governorgener, saxoni, hellen, nco, hussar, silesia, overrun, charlemagn, sindhi, botswana, lombard, revit, baton, gurkha, bipartisan, nonmuslim, sabha, romantic, ssr, viceroy, gregorian, kiev, overseen, mahayana, vassal, pacifist, slav, traditionalist, shogun, manchuria, adventist, togo, ministeri, byelect, thebe, oecd, hun, uboat, leftist, overthrown, highrank, envoy, augustinian, macedon, primaci, taoism, javanes, breton, ioc, forerunn, thrace, loyalist, sworn, jihad, orthographi, planter, prehistori, dictatorship, ugandan, mali, damascu, frenchspeak, selfdefens, dogma, emissari, caucasian, wwii, māori, calvinist, gloriou, reichstag, angola, bourbon, cypriot, ismaili, guatemalan, basqu, distrust, syncret, tutsi, manageri, pashtun, bangladeshi, imperialist, admiralti, judah, bjp, anglo, pacifi, indoeuropean, sami, fief, justinian, mauritiu, bishopr, prc, kurd, moro, geologist, frontlin, legat, relinquish, prewar, aceh, bosniak, tajikistan, retak, bonapart, oblast, sufi, supervisori, lobbyist, rhodesian, jain, paramilitari, outpost, impoverish, craftsmen, aztec, malayan, anc, rightw, fascism, sikhism, janata, slovak, afrikaan, navajo, choctaw, upperclass, mecca, landown, vichi, populac, gaul, saladin, bern, commanderinchief, mesoamerica, reactionari, despot, environmentalist, carolingian, jordanian, antitrust, depos, sassanid, fugit, pontif, archdioces, resettl, novgorod, spd, boycot, polynesia, flander, romanesqu, faa, appointe, fide, forbad, federalist, armada, ashkenazi, galicia, agrarian, uphold, siames, antiwar, vernacular, cloister, vanguard, enclav, industrialist, cognat, uae, hasid, bourgeoisi, bahrain, guatemala, baath, venezuelan, contra, nonpartisan, zionist, sixyear, scientolog, decentr, mep, sudanes, promulg, constabulari, disobedi, pompey, gunboat, moorish, businessmen, gujarati, encroach, fundamentalist, madagascar, shang, khanat, expuls, friar, eritrea, traine, mesopotamia, shinto, selfdetermin, mysor, papaci, balochistan, emancip, corsica, benedictin, crimea, tatar, karachi, anglosaxon, wto, insurrect, haitian, goguryeo, uttarakhand, skirmish, nicaraguan, brunei, undocu, yemeni, syriac, chechen, latino, sizabl, wight, kyrgyzstan, legitimaci, mandarin, cosponsor, solicitor, muster, piou, eurozon, secretarygener, tokugawa, nara, sephard, dravidian, rebelli, mozambiqu, inquisit, armistic, hmong, hellenist, mauritania, horticultur, florentin, cambodian, diocesan, dday, turnout, nascent, tipu, discont, boer, militarili, enslav, anatolian, naga, bolivia, maoist, bureaucraci, ascet, azad, stateown, aden, turkic, judea, venetian, flemish, surveyor, impeach, rajput, illyrian, moroccan, cham, vedic, repatri, barrist, moldova, judiciari, consecr, conservat, islamist, diaspora, latvia, jharkhand, renounc, medina, baku, sectarian, plebiscit, dal, politi, magnat, maldiv, flotilla, reunif, nepales, phoenician, abdic, pentecost, adjut, etruscan, mubarak, bolivian, candidaci, cochair, embargo, reaffirm, daytoday, anticommunist, poorest, jammu, pla, upheav, libyan, chairmanship, indentur, kmt, repar, checkpoint, siam, ghetto, cochin, goa, israelit, fatah, ratif, workingclass, bnp, maratha, overwhelmingli, counterinsurg, airspac, moot, ecuadorian, castilian, liechtenstein, crimean, repudi, mobilis, supremaci, mercantil, disciplinari, revok, unionist, presumpt, oversight, overlord, bavarian, pillag, jainism, congoles, influx, assyrian, demographi, autocrat, plo, coptic, whig, strategist, mla, affluent, peshawar, surinam, inca, shiit, catech, brahmin, sardinia, osteopath, orissa, ndp, tanzania, writ, staf, magyar, tenet, cantones, chola, polynesian, riga, moravia, hondura, philanthropi, ecumen, nasser, multicultur, kurdistan, gdr, scythian, sikkim, clandestin, leftw, rwanda, tunisia, kenyan, heartland, musharraf, multilater, peasantri, fluent, beliz, idf, pantheon, jamaican, ltte, messian, totalitarian, cameroon, middleclass, genoes, labrador, hiroshima, nongovernment, statewid, thracian, statesman, levant, visigoth, samoan, usurp, qin, desecr, crete, ionian, foothold, ardent, steward, umayyad, pogrom, zionism, reconstitut, cityst, ashoka, jurist, cossack, mausoleum, diocletian, assam, mennonit, auspic, cpc, richest, loanword, scandinavian, dalmatia, dacian, berber, battlecruis, peruvian, governorship, chieftain, iberian, tagalog, secretariat, shipbuild, gazett, abbasid, arama, malabar, myanmar, neolith, liaison, erstwhil, slovenian, pursuant, siberia, statehood, dignitari, indochina, circa, nguyen, registrar, toppl, synod, reassert, interwar, unarm, lordship, sympath,

$\mathcal{V}_7 =$ tyre, pigeon, flora, mous, upright, chicken, groov, ant, tap, coconut, hind, knive, rig, curl, hat, corn, tooth, marbl, underwat, cooki, foil, lit, bud, wrap, beneath, snap, feather, cosmic, potato, pie, explod, timber, wolv, burrow, fuse, bread, eject, stuf, rack, plenti, gray, pepper, helmet, blown, deton, shade, calib, grill, owl, worm, straw, granit, badg, collid, deer, chrome, lace, pile, weld, outfit, tile, ribbon, chocol, miniatur, axe, tilt, beard, whip, monkey, haul, candi, sabbath, basement, bamboo, spike, pod, crest, cam, candl, nut, envelop, juvenil, bark, mint, brass, limeston, fog, shirt, powder, chees, cherri, skeleton, saddl, dot, garment, bolt, enclosur, dungeon, sandston, wrist, furnitur, salad, blond, axl, adorn, whistl, lotu, bake, duck, dune, pork, velvet, collar, canva, comb, peanut, lamb, weav, rip, fri, enclos, squirrel, locker, stuck, bracket, butter, click, blast, triangl, exot, camel, cart, robe, clutch, engrav, spray, brick, pyramid, pale, brace, strand, cream, pink, torch, carpet, mast, grip, scratch, chick, knife, aluminum, cab, hollow, underneath, darker, thrown, wasp, vent, jupit, rib, spider, bitter, bite, toss, stripe, dash, slice, flesh, jewel, larva, dwarf, omega, diver, pickup, leather, crab, lean, liveri, toe, rabbit, fauna, pierc, twist, lip, cement, flip, bucket, pencil, mat, shed, honey, dip, tongu, turret, halfway, burst, punch, deflect, coral, forg, brew, bead, stalk, dairi, swan, crocodil, motif, wash, comet, pizza, rainbow, wore, aquat, backward, nail, mosaic, jar, emblem, roast, orchard, piston, pedal, chi, crane, magnum, belli, violet, ceram, wax, arrow, strap, blossom, cow, bumper, beef, shake, flavour, maiden, shotgun, lever, sleev, goat, reef, herd, preciou, debri, alley, screw, bug, pant, tattoo, bent, mortar, shine, vault, pan, ginger, bee, beetl, mule, fuselag, hung, lemon, onion, kitchen, pad, lizard, liquor, rotten, coaster, jewelri, dial, crescent, trim, claw, recip, banana, pot, curtain, smell, crawl, hood, replica, fade, scrap, plaqu, blank, bloom, pulp, flour, fed, mud, butterfli, dust, wheat, purpl, swallow, peel, wool, willow, mapl, poni, chin, spear, tan, noodl, gravel, crust, soup, cockpit, sandwich, scroll, pig, dye, sheep, eclips, botan, leap, fin, lantern, tini, textil, thread, butt, dinosaur, dig, basket, oval, trouser, header, mushroom, lightweight, lightn, illumin, fender, nickel, void, sock, ore, roller, chop, discard, ink, sauc, turtl, swing, stain,

atp, frozen, rectangular, shave, diagon, grab, ivori, glove, pour, herb, ornament, shower, insignia, sox, slip, trout, elbow, blanket, exterior, ceil, cigarett, asteroid, brush, gem, venom, pigment, shark, ladder, drift, juic, stair, lime, dump, shaft, horizon, mold, dug, glow, spiral, sour, torn, skate, cheek, pea, silk, leaf, spun, sink, capsul, rim, flush, floyd, dessert, thumb, jacket, heel, accessori, cane, bounc, potteri, dirt, leopard, grape, rope, fur, launcher, microphon, stitch, choke, liner, mantl, bean, moth, tomato, nake, cage, cone, tin, frog, eleph, hammer, grenad, crimson, reptil, thunder, amber, bathroom, chili, tshirt, tight, hut, eaten, needl, batter, cube, har, warp, cutter, throat, hatch, spice, vine, toilet, burnt, plug, hook, salmon, retract, cake, bubbl, skirt, balloon,

$\mathcal{V}_8 =$ devi, lester, wrestlemania, lili, calvin, stephani, freddi, akbar, sandra, agn, kathi, humphrey, philipp, mickey, doc, liu, vishnu, ronald, gil, stevi, patern, dant, jami, ned, rudolf, anton, piu, melissa, rao, col, winston, louis, clive, bonni, saddam, andr, gu, seth, wang, aaron, liam, valentin, edmund, patti, isaac, paulin, wu, brotherinlaw, maggi, judi, sue, lionel, brad, doug, shannon, darren, clarenc, randi, tina, jeremi, kyle, ronni, niec, marvin, hermann, joel, cum, stan, trevor, geoffrey, hassan, betti, nina, madhya, jeffrey, gustav, mahatma, pierr, horac, nicol, wong, trent, sharon, lynn, zhou, holli, ludwig, hulk, wolfgang, eleanor, dee, swami, benni, emma, rachel, miranda, tel, leigh, eugen, elton, bo, mo, basil, mohammad, xavier, yu, yi, rama, hannah, sherlock, clement, timothi, halfbroth, bryan, bori, marilyn, erik, edwin, wei, shirley, grandson, nephew, kirk, indira, allison, anni, friedrich, shane, hal, shri, rupert, sidney, kati, chiang, mauric, archi, luci, isabel, travi, paula, helen, omar, alexandra, ernest, tai, karen, buddi, maid, herbert, johann, isabella, qb, sy, gregori, marc, bernard, marion, barack, jennif, andrea, glenn, bon, barney, butcher, katherin, leonard, jenni, vanessa, laurel, dalai, prof, ahmad, kitti, jess, cao, vladimir, jacki, ahm, lesli, marcu, daisi, sophia, lyndon, patricia, lil, dana, jessica, boyfriend, amanda, marti, felix, alfonso, jo, christin, constantin, pratt, laurenc, sonni, wilhelm, debbi, shawn, chen, joan, emili, sheikh, jerom, perci, ethan, sen, conan, mama, edgar, reverend, kurt, befriend, dorothi, diana, fu, dale, vic, lauren, ashley, kicker, bruno, mose, fritz, mick, dwight, sara, alia, noel, augustin, uttar, dexter, ernst, cowritten, heather, byron, franki, robbi, josh, dudley, guru, mikhail, theodor, julia, matern, kumar, heinrich, brett, malcolm, teresa, abdul, clair, vernon, christina, bing, judith, brendan, granddaught, randolph, ellen, jin, glen, benedict, te, petersburg, loi, janet, sebastian, laura, baba, claud, raj, donna, clyde, raymond, liz, wendi, florenc, nichola, lindsay, rev, gerald, woodi, ho, lok, magnu, juliu, ivan, jule, leopold, mel, rené, tottenham, romeo, madam, teddi, abdullah, salli, grandmoth, angela, trinidad, cal, carol, cyru, fr, allan, vinc, moham, françoi, empress, jacqu, brittani, notr, stella, noah, jake, kenni, congressman, maj, nathan, martha, milton, consort, herman, vincent, joey, seymour, walli, und, nigel, molli, eva, kapoor, nicola, abba, peggi, gerri, zhang, adrian, lt, ibrahim, cecil, mozart, jill, kenneth, colin, rodney, julian, hey, sid, conrad, olivia, krishna, nanci, ricki, brandon, imam, neal, raja, adolf, derek, goldman, joshua, lou, alma, linda, antoni, eli, otto, augustu, gloria, lanc, leonardo, sophi, rita, franz, beth, roland, kuala, ruth, dian, chad, fanni, rex, carolin, andhra, hank, mistress, rebecca, lin, natali, bart, traci, frontman, tara, catherin, geoff, née, ferdinand, helena, maharaja, elvi, yang, aunt,

$\mathcal{V}_9 =$ dealer, mortgag, agenda, forbidden, embodi, lab, propon, whenev, medit, compli, behav, credibl, deficit, organiz, gambl, durat, bilater, discrimin, heroin, advocaci, penal, portfolio, scholarli, cheap, rhetor, overview, abort, uncertain, biblic, comprehens, uncommon, pursuit, sociolog, depriv, abstract, strictli, sentiment, perpetu, rehabilit, inclus, proven, inspect, anonym, monetari, self, bigger, identif, pronunci, prevail, strict, pleasur, ordin, escal, sudden, incorrect, formul, implic, firearm, tender, justifi, articul, dictat, judgment, abbrevi, relax, conjunct, liabil, terminolog, proposit, retriev, augment, shorten, overlap, weaken, traffick, lifestyl, statutori, imit, legitim, contradict, reliev, curriculum, bia, monopoli, proce, deem, antisemit, specialti, ideolog, contrari, placement, habit, stanc, conceptu, restructur, albeit, hierarchi, voluntari, specialis, loyalti, humanitarian, theft, copyright, etymolog, ambigu, discours, setup, immens, privaci, inconsist, classroom, metaphor, endur, methodolog, synonym, remedi, authent, silenc, simplifi, taxat, intact, alarm, procur, conspiraci, disclos, feasibl, steadili, vital, incomplet, wholli, verifi, workplac, plagu, maxim, appreci, norm, reward, infring, constraint, dealt, concurr, tough, compel, criteria, assumpt, homosexu, imageri, fratern, critiqu, manifest, omit, endang, racism, inabl, predomin, lineag, adher, anticip, humor, complianc, vocabulari, quran, complement, expenditur, fulfil, correctli, diminish, strongest, harsh, broadli, feminist, peer, profound, mediat, nonetheless, puzzl, eas, accordingli, modest, explicit, openli, flaw, partit, sophist, artifact, cope, practition, unrel, adequ, donor, claus, seemingli, forecast, spite, disagre, irregular, deepli, inher, hypothesi, largescal, chemistri, crucial, confin, fiscal, guidanc, aspir, obscur, realist, convey, frustrat, absent, breach, outlin, buyer, offenc, disagr, wisdom, postal, submiss, conform, royalti, compromis, extraordinari, obviou, merit, broader, healthi, properli, overcom, stereotyp, prioriti, systemat, affirm, quiet, chao, encompass, undertaken, capitalist, logist, aesthet, analyz, rigor, charit, poorli, scenario, healthcar, adject, neglect, provok, repress, astronom, segreg, oppress, verb, essenc, racial, guidelin, explicitli, deterior, fraud, enlarg, distant, collector, deduct, pace, buddha, steadi, autonomi, government, disadvantag, burden, alert, fare, offend, exempt, compulsori, wors, tendenc, trait, enorm, enlighten, noun, discourag, wartim, advent, singular, fault, accent, astronomi, everyday, mandatori, freeli, visa, insight, genuin, harass, assur, harmoni, overwhelm, primit, scope, obstacl, heal, premis, regardless, underw, categor, aros, unclear, verbal, boost, lend, percept, non, plural, wherebi, conscious, likewis, expertis, geolog, tenant, inevit, uniti, sphere, anthropolog, trademark, necess, inventori, incent, undertak, regulatori, assimil, virtu, conceal, moreov, prescrib, profess, consciou, exam, forens, registri, iso, pharmaceut, clone, embrac, devis, consensu, undermin,

$\mathcal{V}_{10} =$ sol, roo, à, libertador, rivera, barrio, dauphin, carmen, flore, québec, revu, javier, alessandro, roi, iglesia, lope, félix, rodríguez, alfredo, gran, avant, je, león, pérez, banda, français, provenc, ain, rancho, willem, pont, argentinian, sarkozi, peña, oro, ángel, khomeini, marqu, sul, allend, salazar, davao, silvio, chico, mort, delgado, claudio, blanc, antoin, maestro, niño, salina, cid, ole, international, brasil, universidad, córdoba, enrico, navarro, navarr, varga, val, tito, guadalup, banco, mariano, jaim, vila, paolo, côte, benito, guadalajara, nord, garibaldi, bam, vittorio, sergio, castillo, qaeda, école, bravo, jardin, witt, hustl, moreno, molina, catalina, rey, comt, batista, serra, rochel, parc, libr, julio, gael, ferrer, bernardo, mend, dio, ortiz, sant, veracruz, américa, estadio, historia, luna, ernesto, vill, eduardo, campo, angelo, españa, pietro, cerro, teatro, oaxaca, laguna, carrera, emilio, vasco, ignacio, opu, perón, haut, toro, toma, lombardi, hernández, terr, marcello, ricardo, laci, siena, gonzález, ruiz, deportivo, una, casa, coco, puebla, pico, jong, rossi, estrada, chavez, juárez, tarantino, aux, santana, bella, dei, capon, gómez, fernández, loma, grupo, padr, raúl, nacion, dalí, vita, vizier, gonzaga, lobo, quentin, ramo, roch, méxico, temp, ramirez, della, guerrero, paso, ivanov, blanco, alvarez, asturia, vin, mata, sánchez, mina, stefano, pueblo, khalifa, boi, laval, mal, räikkönen, ciudad, gard, garcía, alonso, yankov, césar, zaragoza, château, sur, guillermo, domingo, nuevo, ramón, ronaldo, francesco, herrera, ou, sera, dia, martinez, mendoza, joaquin, cort, tijuana, arroyo, ayatollah, giorgio, isla, montoya, leyland, aragon, yo, chevali, saba, fontain, sanchez, ferrara, martínez, bel, novo, castil, alejandro, piero, para, canto, aquino, arturo, luigi, messina, pinto, marqui, ligu, gore, federico, romero, dino, mateo, gambino, stade, lair, scala, centro, quito, divoir, museo, guillaum, rodrigo, vida, telenovela, salvador, rizal, nueva, mussolini, palazzo, alamo, por, mond, duchess, national, dom, división, maccabi, trujillo, santand, dolor, ateneo, borg, vicent, verdi, diablo, fray, amor, rue, sonora, vie, fernand, palai, alba, bolívar, samba, aguinaldo, bahia, mayo, primera, femm, felip, hidalgo, cali, cabrera, corté, torino, jazeera, soto, coutur, joão, nort, que, viva, tre, gallo, nadal, louvr, como, río, díaz, martín, monterrey, fernandez, paz, suárez, lac, greco, musé, massa, cesar, enriqu, rosario, société, renn, vall, ponc, giusepp, lópez, fontana, chanel, conquistador, piazza, chávez, cristina, picasso, porta, croix, lux, saud, gonzal, académi, mora,

$\mathcal{V}_{11} =$ roadway, dine, luxuri, unveil, excav, travers, grove, fring, countrysid, sedan, subspeci, harbour, convoy, bend, ridg, trench, thrive, closur, builder, ambush, fortress, frigat, java, voyag, meadow, renault, pipelin,

tanker, att, pave, escort, coastlin, ski, leisur, strait, steep, highland, fountain, perimet, beaver, sm, aerospac, downstream, shelter, scenic, junction, gorg, trunk, bunker, usaf, rebuilt, cedar, ferri, inland, portal, toll, pedestrian, northward, alpin, marsh, subdivid, loung, torpedo, tent, intersect, uss, detach, expressway, pt, plaza, greenland, bangalor, sunk, hemispher, aboard, tractor, freestyl, terrain, raf, boom, cafe, ambul, antitank, lagoon, swept, sniper, café, wreck, ca, il, boe, breweri, wilder, antiaircraft, interst, refineri, toyota, wetland, canyon, cascad, hm, refurbish, fork, armament, observatori, dwell, smallest, bs, demolish, pier, chennai, cruiser, surg, motorway, taxi, waterway, racer, tram, nokia, zoo, aa, pavilion, volcano, lawn, tributari, paradis, palm, ramp, bypass, hike, vineyard, flew, inn, rebrand, harbor, baltic, mk, thame, anywher, warehous, honda, shelf, nightclub, mig, hamlet, pub, fortif, winchest, oak, upstream, hangar, barrack, telecom, quarri, vista, nissan, refug, beverli, pearl, groceri, somewher, crater, slope, mall, ny, arctic, consortium, ranch, distributor, ab, atla, widen, reconnaiss, rug, forestri, pillar, vicin, supermarket, sandi, redevelop, fisheri, parkway, flank, pine, divert, outlet, overlook, cano, facad, mt, aquarium, eastward, monsoon, marina, corridor, cliff, hudson, atop, flown, rocki, ordnanc, depot, erod, ballist, ag, offshor, auxiliari, capitol, encircl, tornado, parachut, swamp, buse, erect, chevrolet, rift, bike, waterfal, mansion, volkswagen, suburban, pa, sank, airbu, antarct, dock, llc, nearest, glacier, runway, refuel, aerial, apach, po, airfield, neighbourhood, fortifi, maneuv, amalgam, gm, sunset, gateway, cf, panama, woodland, chrysler, lodg, erupt, fenc, airplan, surf, plantat, estuari, boulevard, alp, carriag, warship, interchang, hub, amazon, casino, remnant, amphibi, lowland, mi, endem, nile, redesign, stapl, jungl, rhine, prairi, stall, boast, dismantl, battleship, terminu, hawaiian, sanctuari, luftwaff, terrac, nh, altar, haven, courtyard, cottag, en, subdivis, rental, volcan, subway, plateau, battlefield, fs, cater, adjoin, farther, sweep, freeway, reopen, platoon, typhoon, westward, tow, tallest, bombard, delawar, pond, manor, hamburg, wagon, shipment, garag, cruis, flagship, wildlif, cabin, mound, spa, township,

$\mathcal{V}_{12} =$ physiolog, byte, degener, dental, insulin, dispar, radioact, nervou, enzym, varianc, aerodynam, lung, recurr, diagnosi, antibiot, virus, obstruct, collis, diagnos, patholog, textur, fractur, infecti, surgic, implant, facial, mice, decay, inadequ, regener, vertebr, cognit, transplant, evolutionari, viabl, lesion, passiv, limb, thermodynam, socioeconom, arteri, pathogen, volatil, abdomin, irrit, insuffici, neural, gamma, neurolog, sensat, reflex, exponenti, tract, mood, reproduc, sensori, viral, feedback, nonlinear, cardiac, chromosom, uncertainti, momentum, neutron, primat, diagnost, duplic, bacteria, arous, viru, inflamm, impuls, renal, liver, schizophrenia, synthet, deviat, oscil, slight, synthesi, psychiatr, simpler, cerebr, analyt, sigma, chronic, substrat, reactiv, subgroup, vein, phonem, defici, quantit, benefici, morpholog, incur, vibrat, polynomi, nucleu, cardiovascular, coher, encod, seizur, dysfunct, focal, acut, cure, reciproc, fusion, accuraci, allevi, prone, react, ankl, instabl, phi, robust, angular, bleed, headach, genom, drastic, phenomena, gravit, genit, microscop, replic, obes, gland, intercours, malaria, lobe, urin, antagonist, semant, impair, templat, pronoun, shortterm, affin, diverg, trauma, congest, stimuli, nutrit, indirect, unstabl, proton, harmon, autism, cellular, apparatu, distress, anatomi, invert, nerv, scar, fatigu, hormon, tumor, lethal, deform, stiff, prolong, epidem, syndrom, likelihood, catalyst, mild, propag, tempor, mitig, unchang, ecosystem, genera, tens, underli, toxic, meaning, development, posterior, unnecessari, infer, traumat, inequ, advers, pulmonari, exert, queri, conson, stimul, recess, conjug, invers, stomach, nich, tensor, inclin, dietari, caviti, pregnanc, beta, indirectli, bandwidth, drought, parasit, hazard, transpar, spectrum, multipli, symmetr, molecular, grammat, simplest, neuron, causal, occurr, reson, imped, onset, catastroph, regress, breast, anxieti, digest, inferior, invari, alzheim, correl, cord, suffix, hypothes, subtl, kidney, intestin, spatial, suscept, transcript, corros, marker, parkinson, therapeut, degrad, reus, iter, rigid, dimension, syntax, cortex, homogen, paradigm, fever, decod, cannabi, spinal, syllabl, bacteri, subset, coeffici, arithmet, efficaci, cue, repetit, worsen, ingest, pneumonia, semiconductor, nasal, stimulu, antibodi, spine, reproduct, swell, durabl, inhibitor, modal, prostat, polym, peripher, tuberculosi, breakdown, redund, antigen, induc, symmetri, geometr, tertiari, cocain, mutat, addict, metabol, electrod, respiratori, intrins, magnitud, spontan, xray, hiv, rna, precursor, prolifer, equilibrium, prescript, inhibit, arbitrari, pest, pathway, vaccin, anterior, pi, analys, cosmet, isotop, distort, diabet, abnorm,

$\mathcal{V}_{13} =$ gibb, fischer, rodriguez, stalin, goodman, sherman, macdonald, gill, troy, levi, lenin, solomon, pearson, porter, rodger, dunn, casey, cameron, thomson, thompson, stern, lama, berri, boon, bradford, fletcher, gandhi, dame, mater, ferguson, carpent, hawkin, reynold, caesar, wallac, perkin, weaver, barrett, harper, bowi, gould, curri, myer, drake, chapman, byrn, owen, mclaren, hussein, wright, canterburi, cole, forrest, benson, reid, hoover, morrison, sheridan, newton, spencer, bailey, gilbert, fraser, freeman, walsh, emerson, fuller, griffith, carey, jen, starr, morri, scotia, ix, blake, helm, sinclair, livingston, phillip, carrol, levin, quinn, mccarthi, watson, wagner, rahman, xvi, curti, fitzgerald, crosbi, harrison, bro, montana, armstrong, lynch, hammond, elli, xi, webster, walker, lennon, xii, chan, parker, maxwel, archer, tate, potter, edison, dixon, bradi, nichol, osborn, kent, reed, logan, nash, bennett, finn, lang, thorn, allmus, stuart, eisenhow, holden, fisher, whitney, clara, presley, booth, montgomeri, dylan, beck, luther, kay, murray, irv, hogan, cohen, arnold, webb, lyon, lambert, christi, obama, blair, heath, newman, gibson, burk, churchil, shelley, ebert, powel, crow, shaw, eden, carson, truman, watt, wade, jenkin, henderson, butler, harvey, vii, lumpur, mustang, cain, roosevelt, murphi, riley, penn, reagan, sander, mccain, viii, baldwin, monica, boyd, barker, hyde, kane, swift, mann, tyler, doyl, bach, palmer, crawford, coleman, barber, carr, jefferson, schumach, hast, luca, barn, nixon, griffin, md, aka, pitt, mead, sim, koch, macarthur, wesley, oneil, campbel, preston, holm, gardner, dawson, bradley, tucker, sr, vi, meyer, den, fe, hay, hardi, chamberlain, collin, frost, savag, mccartney, mason, morton, robertson, burton, klein, baker, chester, laden, byrd, piper, der, robinson, sterl, norton, einstein, hopkin, sullivan, buchanan, stark, johnston, elliott, duncan, stewart, laud, bacon, hart, moss, hancock, peterson, mitchel, parson, marx, kerri, buck, darwin, mcdonald, turner, hawk, lopez, rockefel, monro, mcmahon, obrien, xiv, richardson, holland, hamilton, xiii, chandler, bryant, cox, weber, joyc, madison,

$\mathcal{V}_{14} =$ anthem, carniv, spinoff, wii, followup, reissu, sequel, conductor, apollo, cameo, slate, vh, eve, gig, unreleas, smart, directori, contributor, trio, embark, sung, ds, horror, rendit, poster, puppet, lp, pb, sang, arcad, facebook, manga, rca, crazi, hd, recount, broadway, genesi, rap, editori, simpson, seller, vol, herald, myspac, rbhiphop, mc, percuss, jam, smash, diari, jockey, funk, bet, preview, cassett, amaz, latest, hail, bollywood, triumph, protagonist, favourit, airplay, forev, regga, guin, rapper, fantast, mtv, artwork, batman, xbox, hardcor, liveact, duo, cheer, choir, recur, commentari, headlin, quartet, rehears, selftitl, con, choru, vinyl, doll, breakthrough, fulllength, oz, dinner, hymn, dancer, maker, trilog, pen, remix, enthusiast, podcast, referenc, telegraph, remast, saga, sitcom, ace, summari, sketch, ballad, cohost, duet, wizard, remak, villain, marvel, hiphop, reunion, soni, melodi, spawn, idol, theatric, lone, backup, epic, aria, teen, zombi, joy, madonna, nintendo, mini, superhero, cbc, bestknown, itv, spiderman, repris, coproduc, emi, recit, monthli, legendari, glori, flute, chat, bang, breakfast, unoffici, showcas, bonu, midnight, shortliv, upload, crossov, banner, greet, bluray, reprint, superman, screenplay, lesson, memor, chant, improvis, merchandis, rereleas, stereo, dirti, catalogu, satir, platinum, tonight, ne, tribut, ensembl, pokémon, thriller, delux, cnn, echo, garner, playabl, instant, numberon, cowrot, parodi, discographi, demo, dj, sonic, imprint, catalog, incarn, scream, hiatu, antholog, disco, upcom, memoir, mgm, trek, itun, circu, dawn, nickelodeon, twitter, espn, photographi, hbo, playboy, footag, autobiographi, clip, riaa, longrun, orchestr, paramount, tragedi, trailer, youtub, mixtap, eurovis, beatl, sega, playstat, miniseri, ap, bside, eponym, blog, repertoir, bestsel, rerecord,

$\mathcal{V}_{15} =$ sc, wwf, tenni, streak, sec, cub, chess, podium, lap, talli, spectat, seventeen, sheffield, texan, rooki, boxer, allstar, wolverin, afl, fixtur, everton, sat, laker, gt, comeback, springfield, sixteen, vacat, softbal, overtim, bulldog, surpass, blackburn, brewer, pageant, tna, cowboy, brisban, tackl, leed, rivalri, fierc, odi, speedway, trainer, conced, roster, postseason, refere, freshman, sack, barcelona, leicest, av, wicket, striker, raven, poker, dodg, wimbledon, penguin, knockout, bruin, eighteen, –present, millennium, acc, sixti, ucla, richmond, scorer,

stint, packer, thanksgiv, quarterback, raider, â, runnersup, dodger, golf, bundesliga, division, hometown, aggreg, stoke, trophi, pac, thirti, semest, thirteen, trojan, flyer, bout, fumbl, $\frac{1}{2}$, sunderland, gymnast, ufc, bristol, nebraska, gator, raini, usc, replay, falcon, nashvil, td, oakland, volleybal, easter, shootout, lacross, maverick, icc, milwauke, brave, hattrick, mlb, marathon, wander, vacant, midfield, nwa, narrowli, vike, pitcher, wcw, bench, ferrari, forti, charger, indianapoli, —, wwe, rbi, jacksonvil, indoor, postpon, adelaid, lotteri, premiership, fourteen, memphi, undef, chelsea, colt, lineback, semifin, sophomor, preseason, fifteen, incept, derbi, smackdown, dart, clinch, starter, cincinnati, runner, hornet, fastest, inning, rebound, bronco, panther, kickoff, cardiff, celtic, offseason, rover, ensu, rejoin, punt, philli, quarterfin, goalkeep, remaind, nhl, threw, arsen, varsiti, ivi, steeler, collegi, tier, qualif, beaten, wigan, ml, orlando, dolphin, firstclass, feud, duel, mascot, columbu, berth, er, panzer, deadlin, inter, motorsport, seahawk, europa, halftim, preliminari, newcastl, sidelin, titan, wrestler, bolton, yacht, alltim, midway, runnerup, releg, autumn, nascar, wembley, yanke, spur, intercept, catcher, rematch, jaguar,

$\mathcal{V}_{16} =$ lao, sailor, ukrainian, kosovo, serbian, loyal, bloc, modernday, slavic, morocco, arabian, ulster, citizenship, guinea, uruguay, dissolut, persia, aristocrat, provision, midland, armenia, colombian, croatian, azerbaijan, arabia, cornwal, taliban, filipino, yugoslav, confederaci, hyderabad, ethiopia, feudal, cuisin, jamaica, refuge, treasuri, ghana, lebanes, cuban, madra, bengal, legion, punjab, ham, istanbul, negro, blockad, balkan, ethiopian, frontier, colonist, malta, concess, ottawa, tang, yuan, nomad, czechoslovakia, ming, gaelic, counterattack, alexandria, vietnames, roma, mercenari, serb, newfoundland, kenya, uganda, gaza, expel, dominion, peasant, gujarat, iraqi, westminst, patriarch, insurg, consul, trader, dominican, settler, migrant, ancestri, mongol, herzegovina, colon, palestin, montenegro, archipelago, syrian, malay, malaysian, mafia, nors, qing, haiti, libya, bombay, pilgrim, papal, hispan, homeland, lithuania, freed, pragu, upris, cede, zimbabw, qatar, yemen, ira, chilean, kerala, detain, isl, commando, macedonian, dakota, besieg, conscript, karnataka, warsaw, pact, canton, kuwait, nepal, hostag, detent, bulgarian, folklor, aborigin, predominantli, protector, normandi, maharashtra, prussian, afghan, victorian, pilgrimag, monarchi, incumb, macedonia, dubai, overthrow, denounc, bosnia, algeria, genocid, militia, cypru, indonesian, sudan, prefectur, albanian, ussr, pakistani, brussel, mongolia, romanian, embassi, congo, napl, catholic, pow, baghdad, vatican, tibet, garrison, cambodia, nobil, saxon, caliph, unif, conting, thai, slovakia, nationalist, lithuanian, constantinopl, luxembourg, passport, yugoslavia, georgian, airway, turk, caucasu, albania, reestablish, takeov, guerrilla, estonia, burma, scot, nigerian, proclam, argentin, emir, partisan, yorkshir, finnish, sicili, fascist, synagogu, deport, flourish, cairo, ecuador, sovereignti, cheroke, napoleon, kashmir, milit, prussia,

$\mathcal{V}_{17} =$ demon, cri, knew, creatur, asid, creator, devil, blind, constantli, wolf, infant, fate, hitler, wed, revel, stolen, competitor, doubt, crush, pretti, pleas, worri, surpris, exactli, lover, wonder, persuad, childhood, accident, trick, els, quick, badli, thank, lesbian, fun, sword, samesex, sure, ye, na, friendship, rider, prompt, tortur, teenag, testimoni, pride, ma, worst, bare, everyon, pronounc, doesnt, insist, aliv, mad, lifetim, grave, certainli, till, killer, disappoint, alien, desper, hate, realis, troubl, ghost, repeatedli, shadow, anyon, fallen, narrat, bride, homer, su, none, spark, captiv, dialogu, temporarili, kidnap, guilti, scandal, joke, wake, ive, monster, versu, recal, quest, angri, resurrect, bless, welcom, toy, funer, confess, robot, wasnt, prostitut, pregnant, specul, anger, imagin, costum, testifi, sin, comfort, forth, conceiv, unfortun, guardian, companion, rape, hang, hide, sacrific, foster, whatev, fake, reportedli, ms, heaven, shall, devast, id, hunter, grace, sick, storylin, suddenli, presum, audit, witch, fortun, mistak, passion, spare, steal, rumor, survivor, interrupt, wrong, truli, boss, acknowledg, suppos, blame, vampir, kiss, inherit, curs, silent, apolog, evil, coincid, betray, contend, etern, guy, mar, upset, deliber, mate, odd, remind, allegedli, cant, jail, portrait, predecessor, complain, laugh, hell, spoke, serious, confid, kid, custodi, hurt, strang, suicid, repli, imprison, beast, aveng, closest, dozen, innoc, confront, reveng,

$\mathcal{V}_{18} =$ sugar, mask, habitat, explos, narrow, flavor, cotton, nois, concret, accumul, tea, sight, bag, carv, arc, insert, tail, thin, cloud, tear, cylind, insect, valv, sand, flash, meat, raw, mammal, tall, arch, clay, drill, pocket, galaxi, shoe, rod, ft, beam, tast, suspens, patch, wire, dive, bore, ammunit, dish, outer, meal, rough, teeth, mirror, tip, barrel, shoulder, hull, stroke, fossil, cattl, fold, shorter, cm, beer, discharg, bow, disk, loop, copper, swim, inner, sheet, plastic, bath, thick, locomot, finger, pistol, float, crystal, diamet, bicycl, neck, blade, steam, pack, penetr, empti, exhaust, ash, bed, chest, steer, gaug, snake, blend, sweet, predat, rat, clock, bullet, pipe, flat, bottl, flame, spin, gear, rocket, motorcycl, wet, tone, axi, gap, fat, tobacco, slide, ear, milk, trap, laser, mercuri, inch, smoke, poison, genu, ingredi, roof, curv, boot, horn, fabric, nest, brake, rubber, skull, deck, polar, cluster, circular, barrier, grain, grass, pet, tire, breath, scatter, pit, knee, mouth, stamp, blow, lamp, stick, soft, chip, hidden, specimen, fragment, outdoor, cartridg, shell, propel, log, harvest, lift, sharp, prey, kit, button, batteri, drag, slot, smooth, delta, whale, crack, medium, pin, bright, coffe, pool, vertic, cannon, artifici, faster, cultiv, horizont, dispos, appl, nose, wooden, egg, metr, alpha, dome,

$\mathcal{V}_{19} =$ mathematician, trumpet, superstar, preacher, sonata, patronag, psychologist, isbn, chancellor, sculptor, encyclopedia, physicist, endow, avantgard, elementari, berkeley, conservatori, rave, beethoven, curat, soprano, modernist, tsar, avid, philharmon, sergeant, archaeologist, reich, comedian, mit, jd, tenor, cyril, brigadi, prolif, unesco, birthplac, manifesto, paperback, apprentic, telugu, malayalam, humanist, deutsch, chef, spokesperson, punjabi, wikipedia, councillor, magna, diploma, bibliographi, magistr, singersongwrit, uc, counselor, truste, biograph, ballet, creed, alumni, newcom, mentor, synopsi, ign, economist, yale, pamphlet, postgradu, englishlanguag, banker, choral, businessman, princeton, smithsonian, math, clerk, coauthor, librarian, sheriff, cornel, thesi, dictionari, superintend, tuition, freelanc, violin, entrepreneur, culinari, seminar, astronaut, urdu, sociologist, forb, screenwrit, petti, emin, troup, vocat, vogu, polytechn, •, pianist, veterinari, discipl, tutor, regent, inspector, yorker, nonfict, biologist, shepherd, concerto, ba, neoclass, rabbi, textbook, abbot, op, preparatori, mba, jointli, filmmak, standup, spokesman, parttim, vicepresid, surgeon, pupil, supervisor, choreograph, pornograph, citat, marathi, bilingu, psychiatrist, playwright, treatis, renown, pseudonym, quarterli, naturalist, bulletin, fellowship, classmat, theorist, kannada, hindi, acquaint, anthropologist, constabl, columnist, baroqu, appel, pharmaci, dissert, shakespear, defunct, saxophon, cartoonist, playback, inventor, grammar, blogger, chemist, instructor, upheld, campus, prose, subtitl, africanamerican, fairi, riff, pp, bengali, thinker, emeritu, technician, stanford, novelist,

$\mathcal{V}_{20} =$ render, sensit, proper, fatal, perceiv, toler, sole, rapidli, earthquak, necessarili, solv, bound, owe, repair, satisfi, emphas, craft, strengthen, explan, wider, ecolog, fals, excit, somewhat, poverti, framework, wealth, tension, simultan, landscap, imposs, manipul, immun, temporari, massiv, exact, matur, grown, behaviour, elabor, outcom, disrupt, closer, emphasi, flexibl, pose, ordinari, defect, minim, notion, complic, understood, circumst, ration, strain, valuabl, superior, similarli, furthermor, longterm, vowel, reli, wage, stronger, exploit, fairli, passag, unless, comparison, dissolv, rapid, counter, extinct, heavili, shock, routin, oral, ongo, easi, accomplish, ignor, afford, modif, absenc, socal, compens, suppress, expos, preval, perspect, partli, phenomenon, vulner, widespread, slowli, moder, therebi, dramat, attain, mainten, confus, character, circul, displac, ideal, absolut, familiar, destruct, reinforc, theoret, violent, margin, deliveri, recoveri, stabl, suffici, resolv, substanti, strongli, aris, unusu, disabl, accur, sort, neutral, huge, facilit, fewer, accommod, attitud, valid, reconstruct, relev, greatli, harm, suitabl, safe, radic, alloc, disturb, impli, optim, isol, persist, weak, easier, visibl, mere, supplement, broad, proof, diet, reliabl, aggress, trend, interfer, borrow, trigger, align, preced, undergo, gradual, gender, loos, clearli, exclud,

$\mathcal{V}_{21} =$ forum, hostil, evacu, counsel, guarante, stake, seiz, constitu, voter, ceas, inquiri, referendum, terrorist, servant, friendli, ratifi, halt, interven, ralli, administ, reorgan, prospect, parliamentari, auction, unifi, advisor, prosecut, equiti, liberti, demograph, eu, rebel, submit, leas, permiss, telecommun, pledg, salari, autonom,

civic, lawsuit, tribal, faction, tribun, withdraw, fulltim, withdrawn, enact, judici, tenur, commenc, physician, consolid, recipi, mandat, contractor, resum, bureau, begun, consent, nonprofit, accredit, reelect, complaint, bid, clinton, sa, diplomat, elector, charter, withdrew, rent, nasa, unsuccess, volunt, behalf, unanim, nationwid, merger, decre, admiss, nato, intervent, propaganda, cia, petit, landmark, repeal, elit, endors, sovereign, elig, deleg, subordin, abolish, spi, commerc, holder, dispatch, licenc, ss, legislatur, analyst, cadet, terror, auto, aftermath, democraci, culmin, registr, oblig, maritim, enlist, archiv, specialist, criticis, clash, warrant, bankruptci, coalit, pension, opt, welfar, interim, advic, condemn, privileg, recognis, prosecutor, workshop, mutual, regain, rebuild, expir, disband, sharehold, slaveri, casualti, sanction, riot, advisori, publicli, fda, prosper, ballot, lobbi, statut, activist, coup, congression, un, supervis, surrend, urg, fbi, renov, overse, postwar, discontinu,

$\mathcal{V}_{22} =$ infrar, vapor, fibr, inflat, nitrogen, residu, ambient, knot, lowest, surplu, absorpt, $-$, mw, silicon, commod, detector, torqu, subsidi, heavier, temper, offset, distil, solvent, dens, gase, ventil, boil, uranium, crude, ph, bulk, melt, humid, deeper, mph, greenhous, gradient, lava, payload, spill, °c, lighter, deplet, muzzl, mb, overhead, abund, sanit, rpm, fatti, upward, plasma, threshold, kmh, hydraul, eros, shortag, alloy, glucos, evapor, cheaper, compart, refriger, sulfur, tide, wavelength, cooler, shear, median, photon, thrust, enrich, moistur, petroleum, gasolin, buffer, freez, width, zinc, cyclon, radiu, boiler, puls, subtrop, veloc, satur, mg, machineri, potassium, sodium, amino, pollut, thermal, combust, shale, dioxid, kinet, diffus, nm, drain, calcium, dispers, emit, tidal, friction, nutrient, propuls, unemploy, ignit, flux, °f, shallow, contamin, freshwat, recycl, turbin, precipit, tariff, clearanc, ethanol, rainfal, sunlight, slower, rainforest, discount, beverag, reservoir, insul, sperm, lesser, intak, aluminium, chlorid, irrig, °, fluctuat, vacuum, latitud, dissip, livestock, solubl, fertil, jaw, manifold, lunar, coil, drainag, literaci, condens, altitud, mortal, sediment, proxim, dose, electromagnet, ferment, rotor, downward, fraction,

$\mathcal{V}_{23} =$ realtim, telescop, cpu, protocol, codic, node, interv, compat, diagram, grid, terrestri, finit, server, lens, io, freight, ps, email, cach, googl, stack, navig, automot, proprietari, sensor, compact, plugin, notat, transmit, static, xp, autom, commut, connector, api, subscrib, portabl, pc, broadband, desktop, q, matrix, gb, browser, layout, ibm, linear, consol, array, android, $\times$, turbo, probe, graviti, modul, mac, kernel, relay, hp, hybrid, denot, $\rightarrow$, prefix, tablet, radar, iphon, ac, quantum, integ, pixel, graph, cc, kw, shuttl, mhz, automobil, embed, supplier, db, vitamin, delet, z, intermedi, vendor, emul, random, simul, gameplay, diesel, chassi, ip, default, gp, laptop, remot, wireless, subscript, newer, bmw, converg, download, topolog, os, prototyp, vector, algebra, scan, encrypt, usb, antenna, cargo, font, transmitt, theorem, leak, surveil, chord, intel, synchron, refin, bundl, amplifi, len, app, readili, menu, interfac, premium, printer, analog, multiplay, reactor, linux, synthes, hardwar, conveni, paramet, dual, infinit, processor, spacecraft, databas, packet, configur, highspe, geometri, discret, binari,

$\mathcal{V}_{24} =$ danni, leo, justin, neil, maria, ibn, nelson, colleagu, kevin, warren, ted, dean, russel, lisa, nova, eric, billi, tim, pat, dick, steven, princess, longtim, teammat, matthew, kim, fred, benjamin, jean, willi, singh, carter, max, cousin, jon, jan, pete, hugh, carl, bassist, kelli, kate, larri, widow, da, craig, ralph, eldest, harold, ron, susan, abu, eddi, santa, lloyd, terri, nick, charlott, franklin, ross, yearold, jay, costar, grandfath, greg, anna, jane, gari, bruce, jeff, alan, charli, shah, elder, wayn, jacob, li, albert, phil, sibl, michel, bin, christoph, drummer, alic, karl, ed, archbishop, ian, ryan, victor, margaret, leon, bobbi, johnni, tommi, denni, rick, ken, robin, perri, luke, todd, ben, sarah, norman, morgan, anthoni, girlfriend, gordon, matt, sean, brook, andi, gen, jerri, donald, evan, graham, dougla, jason, jonathan, barri, oliv, abraham, uncl, reunit, chuck, alfr, brian, roy, walter, cofound, youngest, baron, ami, mario, muhammad, keith, alex, frederick, jimmi, dave, rob, dan, barbara, samuel,

$\mathcal{V}_{25} =$ sampl, expens, classif, index, upgrad, innov, strategi, algorithm, otherwis, enhanc, topic, difficulti, wherea, stabil, variabl, equival, input, usag, experiment, automat, evalu, client, visual, context, motion, coordin, fundament, shift, discoveri, graphic, dynam, mode, intens, accid, represent, classifi, segment, util, variat, revers, differenti, variant, modifi, evolut, laboratori, fast, monitor, revis, core, virtual, assess, error, logic, henc, dimens, map, zero, enabl, mathemat, pure, transmiss, delay, sustain, procedur, calcul, essenti, alter, evolv, handl, extern, correct, weather, appropri, composit, bit, packag, orient, check, add, specifi, extra, predict, descript, equat, statist, precis, scheme, manual, balanc, updat, fix, andor, divers, partial, strength, manner,

$\mathcal{V}_{26} =$ hungari, contin, exil, mumbai, norway, turkey, ukrain, patriot, tokyo, beij, frankfurt, caribbean, ontario, bulgaria, athen, delhi, romania, nigeria, afghanistan, peninsula, lebanon, cuba, taiwan, belgium, cemeteri, austria, iran, iceland, malaysia, munich, finland, hawaii, switzerland, northeastern, greec, vancouv, jerusalem, neighbor, fled, thailand, mainland, alaska, sieg, amsterdam, queensland, geneva, croatia, southwestern, hampshir, venic, glasgow, villa, serbia, peru, netherland, nevada, manila, brazil, emigr, annex, pirat, dublin, indonesia, syria, ambassador, metro, chile, summit, madrid, invad, singapor, orlean, northwestern, southeastern, shanghai, portug, colombia, edinburgh, poland, montreal, alberta, moscow, sweden, presentday, venezuela, bangladesh, denmark, milan, elsewher, vienna, argentina, quebec, abroad, neighbour,

$\mathcal{V}_{27} =$ parad, injur, slow, trace, nicknam, touch, oppon, caught, pull, ahead, wound, penalti, crowd, chase, broke, induct, vs, journey, fought, straight, bat, sail, besid, row, climb, ram, longest, shut, cap, twin, knock, twelv, bought, disappear, struck, jump, stood, departur, twice, trip, broken, span, driven, substitut, laid, pitch, suspend, ward, throw, twenti, tiger, retreat, lane, hurrican, kick, rush, goe, u, ran, drove, whilst, drawn, eleven, giant, gone, buri, tripl, gang, wait, drew, plu, sit, strip, fell, catch, exit, warrior, lay, push, readi, collaps,

$\mathcal{V}_{28} =$ coron, worship, mytholog, sultan, abbey, shrine, monk, monasteri, mosqu, wealthi, persian, realm, ce, missionari, han, thcenturi, ruler, shiva, feast, antiqu, bce, ruin, heir, rebellion, myth, renaiss, priest, conquest, chapel, cathedr, revolt, ancestor, mystic, descent, commemor, surnam, goddess, burial, buddhist, gothic, tibetan, byzantin, mediev, onward, throne, rite, nobl, sikh, clan, denomin, alphabet, proclaim, mughal, conquer, prophet, hindu, crusad, buddhism, dynasti, patron, monarch, ascend, sanskrit, deiti, flee, calendar, inscript, massacr, treasur, armenian, sacr, counterpart, archaeolog, monument, saudi, tomb,

$\mathcal{V}_{29} =$ shape, sequenc, abil, signal, target, integr, qualiti, uniqu, interact, symbol, etc, presenc, detail, directli, devic, focu, equal, eg, principl, fit, resourc, factor, categori, pattern, knowledg, messag, user, definit, advantag, mass, contrast, capabl, characterist, environ, correspond, detect, consum, interpret, matter, distinct, phase, demonstr, sens, reflect, transform, item, kind, impact, techniqu, root, option, deriv, simpl, analysi, solut, consider, content, tool, skill, compon, display, mechan, multipl, basic, restrict, safeti, altern, address, consequ, ie, implement, aspect, electron, instanc, ident,

$\mathcal{V}_{30} =$ wast, bind, dri, membran, storag, depth, fluid, clean, consumpt, oxid, orbit, substanc, radiat, atmospher, fuel, compress, skin, solar, adjust, fruit, heat, veget, deposit, soil, hydrogen, protein, crop, layer, acceler, decreas, feed, emiss, particl, oxygen, tissu, inject, load, maximum, ion, carbon, atom, filter, pump, dna, fiber, exposur, acid, reduct, solid, bone, muscl, mixtur, angl, tropic, molecul, warm, coal, stem, tube, salt, absorb, cool, miner, receptor, liquid, rotat, fresh, drink, optic, excess, extract, alcohol, constant, minimum,

$\mathcal{V}_{31} =$ photograph, occasion, possess, cite, ban, enjoy, pilot, request, depict, suit, confirm, unlik, guid, meant, pursu, abandon, rescu, repeat, encount, descend, favor, obtain, watch, chosen, distinguish, incorpor, dedic, paid, respond, choos, fashion, sought, search, warn, explor, invent, convert, preserv, experienc, perman, permit, regist, introduct, convers, encourag, gather, assign, engag, count, ensur, creation, seek, grew, restor, kept,

threaten, attribut, buy, recruit, accus, deni, send, deliv, recommend, recov, belong, princip, split, accompani, conclud,

$\mathcal{V}_{32} =$ currenc, enterpris, membership, certif, household, expans, tourist, sector, loan, interior, subsidiari, insur, net, ltd, payment, export, consult, farmer, rural, disast, visitor, inc, renew, worldwid, destin, partnership, profit, fair, relief, asset, merg, fee, budget, geograph, ticket, viewer, revenu, residenti, exclus, survey, entiti, sponsor, cash, transact, compris, recreat, crisi, estat, censu, investor, trust, employe, vast, ownership, chariti, ministri, illeg, patent, acquisit, infrastructur, ventur, debt, co, donat, tourism, domest, retail, newli, telephon, financ,

$\mathcal{V}_{33} =$ austin, virginia, maryland, kentucki, connecticut, atlanta, jersey, portland, seattl, boston, oregon, kansa, chicago, manchest, iowa, wisconsin, melbourn, avenu, reloc, pittsburgh, illinoi, baltimor, michigan, ranger, arizona, downtown, miami, liverpool, brooklyn, houston, phoenix, detroit, arena, toronto, dalla, colorado, birmingham, louisiana, denver, philadelphia, pennsylvania, texa, berlin, suburb, tech, minnesota, cardin, manhattan, buffalo, indiana, usa, fc, utah, massachusett, metropolitan, cleveland, georgia, missouri, florida, alabama, sydney, borough, ohio, arkansa, oklahoma, tennesse, mississippi,

$\mathcal{V}_{34} =$ storm, plate, cycl, boat, chamber, rear, winter, bomb, apart, meter, floor, leg, bottom, steel, frame, burn, flood, ring, door, insid, wave, fli, bar, switch, panel, tabl, block, attach, height, column, parallel, spring, glass, tank, onto, edg, gate, mill, stone, forward, bond, lock, wheel, circl, vessel, crash, deep, chain, mm, stream, seed, shop, path, circuit, pair, wood, garden, tower, feet, fill, mount, foot, factori, plane, truck,

$\mathcal{V}_{35} =$ medicin, cooper, attract, statu, librari, mainli, declin, formal, prepar, focus, hospit, agent, demand, programm, architectur, whole, organis, recogn, themselv, file, divid, attent, foundat, agenc, purpos, primarili, discuss, mostli, document, legal, teach, benefit, domin, conduct, controversi, employ, regul, basi, effort, aid, exhibit, mission, economi, intellig, custom, job, cours, situat, convent, money, emerg, oppos, aim, branch, progress, expand, opportun, secret, worker, contact, conflict,

$\mathcal{V}_{36} =$ convict, ‘, someon, answer, hear, divorc, herself, neither, didnt, truth, nor, im, alon, mind, heard, impress, babi, admit, sentenc, promis, fear, dead, punish, perfect, noth, older, anyth, bad, sleep, remark, gift, wish, moment, birth, wit, victim, convinc, spirit, everyth, intent, talent, commit, jesu, notic, inde, suspect, soul, unknown, realiz, mysteri, whi, occas, beauti, coupl, happi, chanc, rememb, gay, holi, dream, listen,

$\mathcal{V}_{37} =$ sourc, concept, method, problem, protect, appli, rather, object, experi, rel, subject, particular, measur, individu, occur, reason, condit, combin, certain, specif, improv, normal, theori, express, concern, complex, approach, evid, sound, physic, typic, imag, structur, properti, applic, materi, function, formula, itself, defin, signific, element, observ, speci, remov, code, indic, compar, valu, therefor, either, data,

$\mathcal{V}_{38} =$ account, futur, learn, shown, suggest, seen, recent, face, particularli, achiev, relationship, past, adopt, introduc, hold, propos, full, memori, charg, reveal, surviv, maintain, separ, numer, carri, contribut, today, share, respect, promot, subsequ, regard, select, key, particip, gave, advanc, earn, accept, despit, saw, especi, rais, gain, whose, identifi, except, least, argu, toward, extend,

$\mathcal{V}_{39} =$ toni, clark, o, miller, tom, bell, jordan, scott, adam, harri, jone, marshal, frank, ford, brown, kennedi, jr, chri, allen, johnson, mike, moor, simon, howard, anderson, knight, don, bush, ray, jack, van, daniel, jim, von, roger, lee, iv, taylor, jackson, lewi, joe, davi, sam, biographi, wilson, ali, steve, smith, khan, bob,

$\mathcal{V}_{40} =$ phrase, bibl, prayer, legaci, illustr, ritual, poet, tale, liter, gospel, narr, poetri, scholar, influenti, cinema, dub, amongst, reader, hebrew, essay, linguist, devot, romanc, painter, legend, dialect, chapter, icon, philosoph, spoken, poem, manuscript, speaker, vers, heritag, spell, canon, romant, cult, quot, chronicl, literari, earliest, wellknown, sculptur, reviv, pioneer, script, literatur,

$\mathcal{V}_{41} =$ bird, yellow, sun, wine, resembl, lion, dress, grey, flower, spot, dragon, eat, hair, dog, planet, moon, breed, belt, coin, wild, cloth, colour, magic, orang, hunt, iron, eagl, wear, worn, rain, snow, coat, cat, decor, ride, rose, hors, rich, diamond, bull, rice, hole, ice, dark, uniform, seal, cook, bear, shield,

$\mathcal{V}_{42} =$ anglican, liturgi, congreg, sunni, clergi, pagan, ld, apostol, oath, pradesh, lutheran, baptist, methodist, pastor, shia, judaism, theologian, scriptur, brotherhood, rabbin, sect, nun, episcop, cleric, apostl, theolog, basilica, dioces, persecut, secular, hinduism, evangel, ecclesiast, communion, parish, triniti, seminari, jesuit, marxist, christ, mormon, presbyterian, orthodox, anarchist, preach, libertarian, ordain, martyr,

$\mathcal{V}_{43} =$ manuel, juan, rosa, carlo, pedro, são, lorenzo, sierra, di, rafael, josé, giovanni, lui, roberto, mont, pablo, andré, fernando, marco, jorg, gabriel, alberto, silva, aviv, miguel, hugo, ana, cruz, copa, fidel, maría, torr, garcia, monaco, paulo, du, polo, marino, castro, antonio, santo, jose, franco, bernardino, santiago,

$\mathcal{V}_{44} =$ northeast, basin, migrat, cave, corner, creek, resort, southwest, tunnel, railroad, nearbi, southeast, pacif, mediterranean, inhabit, geographi, pole, atlant, coastal, boundari, restaur, municip, canal, dam, desert, km, highway, headquart, ocean, adjac, trail, cape, northwest, hotel, fort, stretch, castl, plain, entranc, beach, shore, mile, underground, neighborhood,

$\mathcal{V}_{45} =$ sever, manag, chang, base, found, area, provid, although, produc, product, creat, power, intern, complet, report, each, open, line, within, local, act, point, anoth, remain, lead, own, compani, oper, major, addit, accord, continu, receiv, design, set, under, present, build, current, form, hous, same, support,

$\mathcal{V}_{46} =$ violenc, threat, leadership, belief, opinion, faith, recognit, motiv, scientist, resolut, argument, freedom, divin, speech, intellectu, spiritu, duti, philosophi, alleg, advoc, conclus, ethic, disput, moral, corrupt, instruct, exercis, excel, choic, expert, examin, favour, vision, nevertheless, debat, reput, reject, doctrin, disciplin, statement, creativ, dismiss, assert,

$\mathcal{V}_{47} =$ natur, those, special, consist, activ, though, limit, repres, engin, bodi, possibl, market, further, involv, test, project, exampl, model, standard, respons, industri, contain, effect, issu, type, land, event, exist, human, period, class, control, case, term, way, great, process, ad, anim, offer, requir,

$\mathcal{V}_{48} =$ figur, mention, credit, print, pictur, piec, collabor, plot, earlier, label, novel, text, theme, screen, celebr, websit, journal, newspap, adapt, arrang, instrument, press, comment, magazin, scene, audienc, interview, page, letter, volum, articl, voic, paper, background, doctor, inspir, card, edit, fan, mix, paint,

$\mathcal{V}_{49} =$ peabodi, jubile, daytim, pulitz, bafta, primetim, filmfar, prizewin, awardwin, allamerican, nobel, posthum, telecast, desk, firstteam, accolad, emmi, globe, saturn, finalist, cann, sundanc, oscar, nomine, brit, gemini, prestigi, baseman, medalist, carnegi, nielsen, laureat, guild, juno, dove, mvp, honorari, cw, mellon, grammi,

$\mathcal{V}_{50} =$ founder, deputi, formerli, mayor, bishop, meanwhil, successor, chose, chairman, admir, lincoln, advis, secretari, fellow, hire, cabinet, crown, invit, inaugur, ceo, scout, editor, politician, resign, lawyer, colonel, journalist, assassin, mp, veteran, lieuten, architect, chair, rival, renam, presidenti, briefli, victoria, commission, attorney,

$\mathcal{V}_{51} =$ tonn, usd, capita, lb, ton, ago, litr, cubic, cent, trillion, exceed, metric, kilomet, €, pound, gram, annum, dollar, revolv, yen, fifti, euro, kg, kilogram, crore, gallon, kilometr, se, gdp, weigh, acr, gross, hectar, rs,

$\mathcal{V}_{52} =$ cavalri, jet, helicopt, rifl, warfar, battalion, raid, assault, naval, airborn, artilleri, fleet, bomber, missil, guard, strateg, squadron, submarin, regiment, patrol, expedit, fighter, corp, deploy, infantri, brigad, armour, carrier, combat, tactic, aviat, personnel, armor,

$\mathcal{V}_{53} =$ egyptian, portugues, polish, welsh, palestinian, vice, austrian, commonwealth, oversea, nazi, puerto, dutch, czech, scottish, confeder, swiss, hungarian, tamil, continent, merchant, sri, turkish, mexican, irish, iranian, danish, isra, belgian, imperi, swedish, norwegian, provinci, brazilian,

$\mathcal{V}_{54} =$ behavior, mental, cancer, brain, emot, neg, therapi, symptom, psycholog, compound, diseas, abus, clinic, seriou, ill, reaction, depress, gene, treatment, surgeri, biolog, pain, stress, treat, patient, sexual, infect, chemic, disord, failur, genet, risk,

$\mathcal{V}_{55} =$ via, varieti, link, extens, construct, flight, ground, manufactur, store, transport, equip, ship, access, distribut, suppli, rang, facil, space, avail, format, free, commerci, car, weapon, fire, aircraft, food, comput, plant, vehicl, connect,

$\mathcal{V}_{56} =$ coverag, regularli, hollywood, comedi, disney, logo, weekli, anchor, amateur, serial, daili, theater, fox, fm, poll, documentari, realiti, bbc, cancel, holiday, venu, affili, franchis, drama, nbc, mail, syndic, sky, cb, abc, cartoon,

$\mathcal{V}_{57} =$ brief, genr, adventur, tape, disc, photo, compil, biggest, cd, soundtrack, mainstream, dvd, hero, entitl, highlight, signatur, lyric, favorit, ep, christma, audio, certifi, lineup, fantasi, tune, greatest, session, sing, solo, string,

$\mathcal{V}_{58} =$ civilian, jew, citizen, occup, immigr, egypt, era, affair, regim, pakistan, occupi, tribe, settl, settlement, soldier, republ, coloni, israel, airlin, allianc, invas, slave, flag, revolut, alli, troop, camp, philippin, rome, navi,

$\mathcal{V}_{59} =$ previous, soon, simpli, refus, heart, frequent, better, yet, longer, might, hope, agre, actual, intend, commonli, —, here, hard, immedi, unabl, expect, ultim, quickli, alway, alreadi, fail, ever, probabl,

$\mathcal{V}_{60} =$ miss, crew, schedul, youth, challeng, elimin, prior, entri, incid, beat, lose, struggl, driver, strike, compet, owner, crime, offens, enemi, shoot, partner, defend, draw, contest, tie, latter,

$\mathcal{V}_{61} =$ abl, claim, doe, must, hand, upon, attempt, still, need, action, order, initi, without, onc, instead, find, should, decid, help, never, right, plan, eventu, tri,

$\mathcal{V}_{62} =$ softwar, advertis, joint, licens, rail, microsoft, transit, onlin, web, instal, digit, mobil, camera, bu, traffic, satellit, platform, window, cabl, phone, passeng, termin, internet,

$\mathcal{V}_{63} =$ appar, desir, fulli, lot, increasingli, highli, beyond, quit, understand, difficult, prove, perhap, prefer, easili, clear, rare, danger, sex, awar, necessari, extrem, tend,

$\mathcal{V}_{64} =$ stop, stand, keep, behind, taken, brought, escap, travel, drop, destroy, fall, rest, discov, captur, sent, save, arriv, bring, visit, drive, stay, put,

$\mathcal{V}_{65} =$ orchestra, hop, acoust, symphoni, hip, guitarist, vocalist, bass, folk, drum, guitar, vocal, keyboard, warner, rb, punk, jazz, pop, rhythm, piano, songwrit,

$\mathcal{V}_{66} =$ japan, canada, territori, zealand, germani, europ, spain, mexico, england, ireland, kingdom, franc, australia, border, china, capit, russia, provinc, itali, scotland, pari,

$\mathcal{V}_{67} =$ dont, think, seem, ask, want, told, feel, felt, happen, realli, done, someth, thought, am, let, know, talk, explain, tell, got,

$\mathcal{V}_{68} =$ concentr, profil, elev, capac, extent, ratio, yield, significantli, domain, incom, output, quantiti, densiti, sum, percentag, slightli, frequenc, effici, voltag, proport,

$\mathcal{V}_{69} =$ bridg, villag, road, front, hill, site, templ, section, street, middl, outsid, rout, valley, mountain, centr, cross, resid, port, nativ,

$\mathcal{V}_{70} =$ again, led, return, die, sign, join, leav, replac, meet, attack, togeth, begin, enter, left, lost, reach, kill, held, mark,

$\mathcal{V}_{71} =$ younger, husband, historian, succeed, alongsid, saint, reign, ladi, lord, queen, emperor, pope, portray, sir, mr, dr, le, captain, princ,

$\mathcal{V}_{72} =$ boy, king, wife, parent, friend, son, murder, marri, father, met, brother, woman, girl, mother, daughter, marriag, sister, whom, child,

$\mathcal{V}_{73} =$ soap, bueno, thth, fifteenth, buckingham, sixteenth, tampa, nineteenth, fourteenth, thirteenth, eleventh, midth, twentieth, twentyfirst, twilight, eighteenth, twelfth, pga, seventeenth,

$\mathcal{V}_{74} =$ sinc, origin, member, success, appear, live, life, them, group, against, histori, peopl, home, famili, seri, show, countri, perform,

$\mathcal{V}_{75} =$ park, side, along, region, across, river, western, central, london, built, northern, town, eastern, island, near, locat, throughout, southern,

$\mathcal{V}_{76} =$ undergradu, cambridg, oldest, oxford, lectur, bachelor, faculti, enrol, graduat, phd, professor, taught, teacher, scholarship, nurs, campu, harvard, galleri,

$\mathcal{V}_{77} =$ legisl, administr, seat, committe, congress, vote, parliament, campaign, opposit, commiss, council, leader, governor, senat, bill, candid, assembl,

$\mathcal{V}_{78} =$ june, born, februari, novemb, august, januari, career, juli, septemb, forc, york, decemb, march, april, octob, announc, began,

$\mathcal{V}_{79} =$ given, person, allow, result, becaus, consid, veri, even, refer, among, describ, mean, give, see, make, caus, due,

$\mathcal{V}_{80} =$ oil, spread, upper, urban, climat, lie, portion, agricultur, ga, zone, farm, forest, surround, bay, wind, flow, mine,

$\mathcal{V}_{81} =$ sunday, monday, saturday, pm, walt, tuesday, afterward, wednesday, weekend, shortli, friday, thereaft, weekday, morn, thursday, afternoon, newscast,

$\mathcal{V}_{82} =$ medici, bayern, aston, jure, plata, liga, facto, sall, moin, atlético, rothschild, havilland, janeiro, palma, vega, versail, gaull,

$\mathcal{V}_{83} =$ b, c, x, v, iii, g, e, r, f, j, d, k, p, l, w, h,

$\mathcal{V}_{84} =$ next, summer, previou, ten, hour, five, everi, nine, seven, six, eight, big, hall, night, entir, few,

$\mathcal{V}_{85} =$ research, commun, servic, inform, studi, scienc, educ, organ, econom, center, institut, polit, busi, program, train, social,

$\mathcal{V}_{86} =$ elizabeth, loui, arthur, alexand, stephen, patrick, martin, mari, philip, joseph, lawrenc, andrew, franci, edward, ann, duke,

$\mathcal{V}_{87} =$ blood, gun, light, tree, wing, room, eye, sea, machin, surfac, heavi, color, earth, metal, wall, fish,

$\mathcal{V}_{88} =$ draft, nba, pick, rugbi, nfl, squad, junior, winner, tournament, playoff, qualifi, confer, ncaa, retir, senior,

$\mathcal{V}_{89}$ = christian, greek, thousand, latin, arab, protest, islam, muslim, hundr, speak, minor, translat, ancient, religion, jewish,

$\mathcal{V}_{90}$ = exchang, sold, acquir, invest, sale, brand, purchas, sell, transfer, corpor, global, fund, pay, stock, privat,

$\mathcal{V}_{91}$ = true, thing, littl, idea, your, question, my, whether, how, our, look, fact, god, too, me,

$\mathcal{V}_{92}$ = famou, note, write, list, short, wrote, danc, classic, compos, collect, notabl, read, written, cover,

$\mathcal{V}_{93}$ = justic, investig, appeal, judg, crimin, approv, declar, constitut, peac, decis, arrest, prison, trial, grant,

$\mathcal{V}_{94}$ = avoid, damag, prevent, suffer, affect, grow, potenti, poor, resist, loss, drug, injuri, strong, lack,

$\mathcal{V}_{95}$ = languag, cultur, practic, word, relat, view, interest, histor, movement, influenc, associ, modern, tradit, societi,

$\mathcal{V}_{96}$ = russian, indian, french, canadian, german, australian, spanish, royal, english, foreign, italian, japanes, chines,

$\mathcal{V}_{97}$ = differ, small, larg, main, ani, close, similar, import, popular, common, good, variou, increas,

$\mathcal{V}_{98}$ = charl, john, georg, robert, michael, jame, paul, david, william, henri, thoma, peter, richard,

$\mathcal{V}_{99}$ = twoyear, lockhe, rhode, oneyear, rio, virgin, zeppelin, fouryear, threeyear, amus, sponsorship, fiveyear,

$\mathcal{V}_{100}$ = athlet, pro, cricket, basebal, profession, wrestl, soccer, bowl, basketbal, hockey, super, stadium,

$\mathcal{V}_{101}$ = final, start, late, second, end, four, earli, until, last, befor, three,

$\mathcal{V}_{102}$ = price, speed, level, energi, pressur, temperatur, degre, rate, water, cell, cost,

$\mathcal{V}_{103}$ = stage, fight, defeat, race, club, battl, victori, player, competit, match, win,

$\mathcal{V}_{104}$ = pass, come, turn, go, came, get, run, date, just, move, went,

$\mathcal{V}_{105}$ = fa, afc, stanley, middleweight, fifa, intercontinent, sprint, nfc, uefa, heavyweight, costa,

$\mathcal{V}_{106}$ = charact, book, music, role, featur, stori, star, titl, direct, version,

$\mathcal{V}_{107}$ = larger, less, below, greater, higher, smaller, abov, reduc, lower, low,

$\mathcal{V}_{108}$ = so, i, we, could, what, do, did, you, if, like,

$\mathcal{V}_{109}$ = negoti, firm, provis, proceed, jurisdict, impos, amend, violat, prohibit, enforc,

$\mathcal{V}_{110}$ = prize, outstand, honor, silver, honour, bronz, ceremoni, medal, golden,

$\mathcal{V}_{111}$ = channel, movi, launch, host, entertain, news, media, broadcast, sport,

$\mathcal{V}_{112}$ = consecut, eighth, tenth, ninth, seventh, rd, sixth, fifth, nd,

$\mathcal{V}_{113}$ = children, himself, young, man, women, age, men, death, old,

$\mathcal{V}_{114}$ = annual, total, tax, percent, highest, rise, estim, averag, growth,

$\mathcal{V}_{115}$ = socialist, reform, labour, communist, liber, democrat, republican, labor, conserv,

$\mathcal{V}_{116}$ = former, attend, chief, assist, elect, board, serv, head, appoint,

$\mathcal{V}_{117}$ = spent, roughli, almost, squar, spend, approxim, worth, nearli, £,

$\mathcal{V}_{118}$ = academ, environment, scientif, technic, medic, primari, financi, health, secondari,

$\mathcal{V}_{119}$ = outbreak, gulf, vietnam, cold, iraq, korean, tag, revolutionari,

$\mathcal{V}_{120}$ = establish, offici, church, rule, offic, author, independ, parti,

$\mathcal{V}_{121}$ = ball, roll, walk, cut, step, break, shot,

$\mathcal{V}_{122}$ = weight, size, length, distanc, enough, scale, amount,

$\mathcal{V}_{123}$ = indi, korea, asian, coast, wale, asia, carolina,

$\mathcal{V}_{124}$ = doubl, femal, male, promin, adult, guest, cast,

$\mathcal{V}_{125}$ = champion, cup, premier, coach, footbal, championship,

$\mathcal{V}_{126}$ = song, top, singl, album, track, band,

$\mathcal{V}_{127}$ = command, polic, staff, execut, post, box,

$\mathcal{V}_{128}$ = green, white, red, blue, black, gold,

$\mathcal{V}_{129}$ = hit, uk, studio, concert, debut, tour,

$\mathcal{V}_{130}$ = versa, latterday, rico, nadu, rica, rican,

$\mathcal{V}_{131}$ = y, grand, et, del, el, al,

$\mathcal{V}_{132}$ = say, said, ', believ, love,

$\mathcal{V}_{133}$ = museum, contemporari, fine, master, martial,

$\mathcal{V}_{134}$ = american, west, east, south, north,

$\mathcal{V}_{135}$ = america, africa, india, african, bank,

$\mathcal{V}_{136}$ = singer, musician, actress, writer, actor,

$\mathcal{V}_{137}$ = secur, task, defens, reserv, defenc,

$\mathcal{V}_{138}$ = through, back, away, down, off,

$\mathcal{V}_{139}$ = third, regular, finish, rank, fourth,

$\mathcal{V}_{140}$ = half, largest, round, decad, quarter,

$\mathcal{V}_{141}$ = now, becom, best, becam, well,

$\mathcal{V}_{142}$ = artist, rock, style, director, video,

$\mathcal{V}_{143}$ = olymp, theatr, festiv, airport, trade,

$\mathcal{V}_{144}$ = yard, field, score, touchdown, goal,

$\mathcal{V}_{145}$ = prix, juri, slam, duchi, testament,

$\mathcal{V}_{146} =$ motor, magnet, real, nuclear, electr,
$\mathcal{V}_{147} =$ million, around, popul, us,
$\mathcal{V}_{148} =$ british, union, presid, govern,
$\mathcal{V}_{149} =$ earl, birthday, grade, anniversari,
$\mathcal{V}_{150} =$ univers, art, law, student,
$\mathcal{V}_{151} =$ billboard, hot, peak, chart,
$\mathcal{V}_{152} =$ usual, often, thu, sometim,
$\mathcal{V}_{153} =$ season, leagu, war, team,
$\mathcal{V}_{154} =$ fiction, technolog, polici, care,
$\mathcal{V}_{155} =$ recept, review, prais, acclaim,
$\mathcal{V}_{156} =$ francisco, angel, diego, kong,
$\mathcal{V}_{157} =$ divis, minut, overal,
$\mathcal{V}_{158} =$ militari, armi, depart,
$\mathcal{V}_{159} =$ opera, marin, palac,
$\mathcal{V}_{160} =$ nomin, academi, won,
$\mathcal{V}_{161} =$ n, m, t,
$\mathcal{V}_{162} =$ lake, britain, deal,
$\mathcal{V}_{163} =$ long, much, far,
$\mathcal{V}_{164} =$ determin, vari, depend,
$\mathcal{V}_{165} =$ known, took, take,
$\mathcal{V}_{166} =$ agreement, contract, treati,
$\mathcal{V}_{167} =$ copi, billion, per,
$\mathcal{V}_{168} =$ televis, episod, tv,
$\mathcal{V}_{169} =$ day, month, week,
$\mathcal{V}_{170} =$ washington, district, suprem,
$\mathcal{V}_{171} =$ soviet, feder, european,
$\mathcal{V}_{172} =$ religi, indigen, ethnic,
$\mathcal{V}_{173} =$ counti, colleg, california,
$\mathcal{V}_{174} =$ mid, bc,
$\mathcal{V}_{175} =$ critic, posit,
$\mathcal{V}_{176} =$ retain, assum,
$\mathcal{V}_{177} =$ ottoman, roman,
$\mathcal{V}_{178} =$ minist, prime,
$\mathcal{V}_{179} =$ ii, civil,
$\mathcal{V}_{180} =$ radio, railway,
$\mathcal{V}_{181} =$ de, la,
$\mathcal{V}_{182} =$ air, arm,
$\mathcal{V}_{183} =$ comic, publish,
$\mathcal{V}_{184} =$ columbia, dc,
$\mathcal{V}_{185} =$ station, network,
$\mathcal{V}_{186} =$ lanka, lankan,
$\mathcal{V}_{187} =$ cathol, empir,
$\mathcal{V}_{188} =$ court, school,
$\mathcal{V}_{189} =$ high, public,
$\mathcal{V}_{190} =$ lo, hong,
$\mathcal{V}_{191} =$ “, ”,
$\mathcal{V}_{192} =$ st,
$\mathcal{V}_{193} =$ san,
$\mathcal{V}_{194} =$ fame,
$\mathcal{V}_{195} =$ award,
$\mathcal{V}_{196} =$ place,
$\mathcal{V}_{197} =$ wide,
$\mathcal{V}_{198} =$ centuri,
$\mathcal{V}_{199} =$ th,
$\mathcal{V}_{200} = \emptyset$

8.3 Companies with the highest daily returns

8.3.1 Sector breakdown within the S&P500 and the dataset

Table 2 contains the sector breakdown within the dataset as well as the S&P500. These numbers are based on S&P500's factsheet from 2022.

Sector	Weight	Percentage in dataset
Industrials	7.8%	16.3%
Health Care	12.7%	11.0%
Information Technology	29.3%	10.0%
Consumer Discretionary	13.2%	10.7%
Communication Services	10.4	3.0
Consumer Staples	5.6%	10.7%
Utilities	2.4%	7.7%
Financials	10.8%	14.0%
Materials	2.5%	6.3%
Real Estate	2.6%	7.3%
Energy	2.7%	5.3%

Table 2 Sector breakdown of the S&P500 by index weight, together with the relative percentages of each sector within the 300 constituents considered in the dataset.

8.3.2 Ticker symbols of the 300 constituents

Here are the ticker symbols of the 300 companies that we considered, in order:

$\mathcal{V} =$ IP, CB, ZBH, AAPL, GS, IBM, AMGN, MMM, CVX, FDX, COST, CMI, UNP, AVB, BLK, SPG, HD, LMT, JNJ, KMB, JPM, GD, MCK, ESS, CI, UNH, CSCO, PXD, MCD, NVDA, INTC, PSA, MTB, HON, BXP, GWW, NOC, TMO, BA, INTU, APD, TRV, RTX, PEP, CAT, AMAT, TXN, ORCL, WHR, BDX, PPG, QCOM, SHW, UPS, PH, LRCX, PFE, NSC, HUM, ECL, DE, ADP, GE, SRE, ROK, WMT, EOG, MLM, PG, RE, DIS, NEE, T, ITW, KLAC, XOM, PNC, RL, AON, EA, LOW, BAC, AXP, VZ, CMCSA, SYK, EBAY, STZ, WFC, HPQ, ROP, AMT, ABT, CLX, BEN, C, LLY, SNA, SWK, MS, CTXS, KSU, MCO, MRK, EL, FRT, KO, HAL, APA, WM, SJM, ADI, DHR, FCX, JCI, VMC, MSI, IFF, SBUX, GILD, CTAS, CVS, ALL, UHS, COO, SLB, MMC, TT, MCHP, NLOK, MDT, HSY, TGT, BMY, TROW, NKE, USB, COP, EFX, XLNX, MO, DRI, ROST, DTE, JNPR, CCI, BBY, NTAP, DUK, OXY, TFX, VLO, LHX, PAYX, FITB, SBAC, ETR, GPS, COF, NEM, MRO, KR, YUM, CL, DD, DGX, WBA, SCHW, MAR, GLW, SO, BK, JBHT, NTRS, PGR, TJX, AIG, ADM, HWM, A, CAG, STT, SYY, HES, ABC, DOV, CSX, EIX, GIS, TFC, WMB, NUE, VFC, ETN, BAX, EMR, EXC, JKHY, CAH, AEP, AFL, XEL, TECH, ATVI, ARE, DVN, HIG, AVY, NWL, WY, OMC, PCAR, FE, D, MAS, POOL, LEN, BBWI, VNO, EQR, NI, TER, CPB, DHI, PEG, K, LUMN, PPL, HAS, MU, MKC, PLD, LNC, ZION, ED, APH, MGM, CNP, PVH, CMA, CTSH, EMN, FAST, TSN, IEX, RSG, AEE, EXPD, TSCO, TXT, ES, CINF, MOS, CHRW, CERN, PBCT, RCL, UDR, CTRA, PEAK, TAP, CCL, SEE, KIM, ALB, KEY, XRAY, RMD, STE, DRE, BWA, WEC, RHI, GPC, FMC, L, J, LEG, OKE, MAA, CMS, PHM, VTR, IRM, PKI, O, ODFL, SWKS, AES, HRL, BLL, AME, AJG, IVZ, RJF, PNR, GL, LUV, IPG, PNW.

8.3.3 Processed sequence of observations

Here is the complete, processed sequence of observations:

$X_{1:\ell} =$ ADI, AES, PVH, HUM, NTAP, AMT, EBAY, NTAP, J, RL, PVH, ROST, ODFL, DVN, EOG, XLNX, ODFL, LOW, A, INTU, CCI, NTAP, ODFL, ATVI, TSCO, EBAY, STE, BLL, MLM, EXPD, CCI, ODFL, LUV, J, AAPL, ZBH, HAS, TGT, ROK, AJG, CTXS, ODFL, NEM, NLOK, ATVI, SWKS, MSI, SWKS, EA, SWKS, MGM, MU, NLOK, JNPR, NEM, JNPR, CTXS, SCHW, SWKS, JNPR, XLNX, CTSH, TAP, USB, FCX, NTAP, NI, JNPR, HPQ, QCOM, SBAC, TSCO, PVH, SBAC, CTSH, SBAC, TSCO, SWKS, MOS, TER, SBAC, MKC, WMB, FCX, RMD, PKI, ODFL, USB, MRO, NTAP, ODFL, NTAP, ODFL, DRI, SWKS, AMT, WMB, EL, TSN, CTSH, ATVI, AES, GLW, ODFL, JNPR, TER, JBHT, HAL, ODFL, HAL, SWKS, HUM, ZBH, NVDA, SWKS, HUM, ABC, STE, HAL, QCOM, RCL, FCX, JCI, LMT, STE, AAPL, HUM, MOS, CCI, GLW, CCI, SBAC, MAR, PVH, PAYX, SJM, STE, TT, ROP, AES, WMB, ATVI, AMT, CCI, JNPR, WMB, SBAC, AES, JNPR, CTXS, ODFL, PNR, ODFL, AES, RSG, FCX, QCOM, PHM, JKHY, FMC, TSCO, JNPR, QCOM, PXD, AES, BMY, ODFL, NVDA, POOL, TSN, MKC, CCI, LRCX, SBAC, AMT, UNH, JKHY, MSI, SBAC, APD, NTAP, STE, NVDA, JCI, CMCSA, JCI, RMD, HPQ, EFX, NTAP, RL, RSG, NTAP, SWKS, CNP, JCI, TECH, CNP, NEM, WMB, SWKS, CNP, NEM, ODFL, NTAP, ROK, SJM,

CTSH, ORCL, ODFL, MCHP, JCI, HUM, AES, JCI, AMGN, SBAC, JBHT, AMT, LUV, AMAT, CCI, AES, AMT, JCI, NEM, JNPR, ORCL, LRCX, CNP, CTXS, UPS, JCI, ROP, NTAP, AMT, SBAC, COF, AES, SBAC, JKHY, WMB, CNP, CCI, WMB, NWL, GLW, MO, MGM, IPG, LRCX, XEL, CCI, AON, SWKS, SBAC, CCI, GLW, WMB, AES, TSN, GLW, TSN, AMT, GLW, SBAC, CCI, NLOK, BBY, JKHY, COF, NVDA, AES, MOS, AES, HON, JCI, SWKS, PEP, DUK, WFC, EIX, MCHP, CNP, MCHP, WMB, DD, MU, VZ, GPS, XEL, BK, DGX, SBAC, AES, AMT, TSCO, APH, MU, AES, XEL, IPG, AMT, CCI, SBAC, WM, PKI, SBAC, AES, JNPR, NI, LMT, SWKS, CNP, LLY, WMB, NVDA, SBAC, WMB, SJM, AMT, SBAC, TER, AMT, SBAC, MSI, TER, HAL, AES, DUK, AES, STE, AMT, AES, LRCX, SBAC, AES, WMB, SEE, CCI, DRI, ODFL, NKE, SWKS, AES, SBAC, ATVI, TGT, WMB, SBAC, CERN, SBAC, GLW, TECH, AMT, LHX, WMB, GLW, STE, SBAC, WMB, SBUX, SBAC, AES, SBAC, TER, VLO, WMB, PKI, NUE, SBAC, GLW, AES, A, AES, ADI, WMB, DGX, SBAC, UHS, WMB, CMCSA, MU, CTAS, BBWI, GLW, CCI, WMB, IPG, GILD, TSCO, IPG, AME, ODFL, SBAC, WMB, SBAC, NTAP, SBAC, FCX, CVS, CMS, CTXS, FAST, AES, MCD, SYK, CCI, TGT, SBAC, CMI, CMS, JKHY, WMB, SBAC, ROK, SBAC, BDX, CMS, TAP, AMT, TECH, AES, SBAC, MCD, CCI, ROST, SBAC, HIG, SWKS, MSI, CTSH, AES, SBAC, INTU, MO, NI, SBAC, TXN, GLW, BBWI, SBAC, MU, CTSH, BBY, MCD, SWKS, MU, AMT, ODFL, PGR, BAX, HON, TER, SWKS, GILD, GPS, SBAC, CMS, CTSH, SBAC, CTXS, CCI, SBAC, SWKS, SBAC, TER, HPQ, GILD, LRCX, BLL, CERN, HAL, TT, CTSH, TSCO, POOL, FAST, CCI, SBAC, CI, CMI, XEL, SBAC, EXPD, RL, BBY, NKE, LRCX, TECH, KSU, TSN, TXN, MU, SBAC, NTAP, NLOK, JNPR, FCX, LRCX, AAPL, PKI, COO, J, COO, UHS, SBAC, NVDA, CMS, ROK, FE, RL, AES, CTSH, SCHW, SLB, SBAC, NLOK, MKC, LMT, AES, GLW, AMT, DHI, CCI, FAST, SBAC, SWKS, ODFL, TJX, CTSH, SBAC, IPG, GWW, EFX, SBAC, CERN, RE, POOL, PHM, STE, RCL, TER, CCI, AES, CI, MMC, EXPD, TXN, TJX, FCX, L, ABC, CERN, ABT, PGR, HAL, FCX, UNH, ATVI, RCL, NTAP, MOS, EA, LRCX, MOS, J, MOS, EOG, KSU, SBAC, CCL, MCHP, SWKS, AES, NEM, RSG, IPG, CTAS, CERN, MCD, SBAC, AAPL, NVDA, SBAC, FMC, MCK, SBAC, IRM, GILD, AMT, SBAC, JNPR, SBAC, TMO, TER, JNPR, LHX, TSCO, CAH, TSCO, TSN, AVY, PKI, LEG, RMD, TSN, PBCT, RMD, TER, WMB, EXPD, FCX, TFX, SBUX, MU, FCX, HSY, MCD, ADI, SWKS, PHM, AES, MCHP, CCI, COO, CCI, AAPL, BDX, AAPL, NLOK, TECH, FAST, HUM, ODFL, CTXS, TSN, MCD, SWK, NVDA, TER, PNR, FMC, NVDA, PBCT, BLK, TRV, SBAC, JBHT, PEAK, CERN, VLO, VTR, ABT, GWW, SBAC, ODFL, MCK, MSI, MCHP, BXP, TROW, AES, LUMN, MCK, EOG, JNPR, SWKS, DVN, TSN, FCX, DHI, FITB, NTAP, CMI, NTAP, NUE, ROST, ECL, PVH, PXD, ODFL, GLW, STE, NVDA, EXPD, AAPL, EA, JBHT, SBAC, MGM, EL, CMS, STZ, AME, AAPL, LEG, CNP, MOS, SBAC, JNPR, SCHW, A, COO, TER, HUM, WMB, MOS, SYK, FCX, XLNX, CTSH, TGT, HUM, CAH, UNH, PXD, MCHP, NTAP, AME, LRCX, HWM, PCAR, CCI, PKI, ZBH, SBAC, ADM, OKE, SWKS, ROST, GPS, MOS, TSCO, ADI, TGT, ADI, LUV, GLW, SWKS, INTU, SWKS, PHM, FMC, ATVI, AAPL, BLK, MOS, COO, ROST, COO, PHM, SBAC, NVDA, SBAC, MCHP, CAH, VTR, IEX, CTAS, TER, PXD, FITB, DRI, HAL, MRK, FMC, SBAC, CTSH, CTXS, SWKS, ORCL, DHR, ROP, SBAC, GLW, SBAC, AAPL, SBAC, GPC, AJG, SWK, TER, MCHP, MMC, VFC, OMC, TMO, IRM, PKI, HUM, AON, EXPD, MO, RJF, ROK, PVH, ATVI, PVH, HRL, EA, LHX, MO, AMAT, EOG, AAPL, CTRA, MOS, NEM, SBAC, LRCX, NTAP, SBAC, NTAP, CL, FCX, XLNX, ATVI, MOS, VTR, PHM, MRK, LEG, OXY, KSU, ODFL, EXPD, CAG, CTSH, MU, TER, HRL, EFX, HSY, EXPD, MGM, AAPL, ODFL, ATVI, HAL, ATVI, PXD, ADM, TGT, INTU, SBAC, ETR, J, AME, NVDA, MDT, NUE, VLO, RL, TSCO, RMD, TSCO, VFC, AON, CTSH, NTAP, ATVI, A, WMB, TSN, MRK, NEM, MMC, PHM, SLB, FMC, CTXS, TECH, FCX, JNPR, TAP, PVH, ADI, SBAC, HAL, MGM, VLO, DVN, HAL, NVDA, PAYX, VLO, FMC, AIG, HPQ, TXN, CMS, VLO, MCK, ODFL, VLO, RMD, TSCO, EOG, SEE, LLY, SYK, ABT, VLO, PVH, GLW, JKHY, GILD, ATVI, PKI, GLW, MCHP, HUM, EOG, PVH, EBAY, CCI, ATVI, GLW, SBAC, GLW, TMO, ORCL, LOW, A, YUM, AAPL, JNPR, AES, SWKS, PVH, CCI, STZ, PEAK, GPS, ZBH, ATVI, SWKS, EOG, MGM, MMC, EA, BBY, SBAC, POOL, TECH, IPG, KR, CCI, DVN, SBAC, STZ, PAYX, VTR, MKC, CTRA, ROK, IVZ, SCHW, TER, EA, RHI, CMI, CTAS, SWK, EA, TER, EBAY, TSCO, WHR, PNR, MCHP, IRM, ATVI, TSN, TAP, PBCT, ROST, NVDA, AME, CNP, APA, MCD, AAPL, NTAP, TECH, HPQ, CMS, CAT, SBAC, RMD, ATVI, ABC, HRL, LRCX, VLO, VMC, HES, COF, AAPL, MGM, COO, ATVI, CMI, RE, MU, MCD, RE, AAPL, NSC, AON, PGR, ALL, EOG, RMD, STZ, EBAY, MAR, EIX, CLX, LUV, TJX, ROST, APH, CTRA, TXN, AAPL, SWKS, MO, FITB, MGM, AAPL, SBAC, CTRA, HPQ, IRM, ADM, MSI, EXPD, JBHT, WMB, AON, GPS, SBAC, LRCX, PVH, BMY, TSN, CERN, EOG, INTU, MCHP, SBAC, DE, MMC, TER, COO, FCX, SBAC, TER, ZBH, GLW, NEM, ES, CTRA, ECL, INTU, PFE, JBHT, MO, DRI, MRK, TRV, GILD, HUM, ODFL, STZ, HES, ATVI, HES, ALB, ADM, LHX, ATVI, DHI, FCX, ATVI, NTAP, PEG, MOS, JNPR, APH, SLB, ETN, CHRW, GLW, TXT, CMI, AAPL, SBAC, MLM, JKHY, MOS, MU, RMD, VFC, TSN, AON, BMY, CTRA, ROP, VLO, COO, RHI, PHM, IEX, ROP, PAYX, JNPR, GLW, ADM, SWKS, MAA, CVS, TSN, FCX, ATVI, JNPR, SWKS, EOG, SBAC, RMD, PVH, IPG, PHM, PXD, CHRW, EBAY, WHR, NLOK, JKHY, SWKS, NVDA, PHM, CCI, AMT, MOS, UNH, HON, VFC, PHM, APH, AME, LEG, PEG, ODFL, TSN, SNA, NVDA, MOS, LRCX, EXPD, ATVI, ADM, HUM, TSN, COO, UNH, TFX, HUM, JNPR, KR, TSN, JNPR, WHR, SBAC, ATVI, RMD, OKE, RCL, JNPR, WBA, DRE, TRV, TAP, NSC, CERN, DIS, NTAP, DVN, SBAC, PXD, CCL, AES, SWKS, AAPL, JBHT, SWKS, MRO, NVDA, FCX, SBAC, WMB, POOL, MO, EIX, GPS, KLAC, ESS, CTXS, DHI, MAR, ODFL, RL, GL, CERN, HAS, ALB, XLNX, IRM, NVDA, HUM, VMC, MCO, PHM, LMT, ADM, EBAY, CERN, CHRW, NVDA, KIM, JNPR, NVDA, EBAY, NTAP, FCX, WY, SJM, MDT, SWKS, ODFL, EBAY, LRCX, RCL, SWKS, DVN, PBCT, PHM, EFX, NWL, BBY, BLK, SWKS, MGM, NUE, MMC, SWKS, OXY, BWA, LRCX, HES, EXPD, EBAY, MCO, DHI, JBHT, NVDA, CMI, DD, NVDA, MRO, XLNX, YUM, LRCX, ETN, CAH, TSCO, CHRW, CERN, EBAY, NUE, NSC, MCHP, PKI, NLOK, NVDA, D, ROST, CTRA, JNPR, BA, ADM, NUE, TMO, LRCX, HD, PNR, NTAP, EOG, LRCX, DE, FCX, PPL, CHRW, CSCO, PXD, PHM, VLO, NUE, GLW, MAS, CTRA, PCAR, NLOK, TSN, COO, WY, SWKS, RMD, NUE, HPQ, JNPR, GPS, SWKS, AAPL, A, IVZ, ODFL, GLW, MSI, GPS, AAPL, COO, BWA, MOS, GS, PHM, PEAK, CCI, COO, CSX, EBAY, FCX, KLAC, JNPR, EOG, CHRW, ODFL, POOL, CTSH, GLW, VNO, MLM, MOS, EXPD, CSX, NTAP, MOS, SJM, VMC, HAL, INTU, TAP, MAS, CI, ORCL, TSCO, KLAC, LHX, VTR, PVH, APH, EL, T, JNPR, MCO, CSX, ODFL, MLM, ROP, CSX, EBAY, LEN, ATVI, HAL, AVB, JBHT, CHRW, PXD, NVDA, AES, BBWI, CMCSA, FAST, PNC, WY, MMC, PHM, PPG, POOL, PXD, RMD, WHR, SWKS, MCHP, POOL, XRAY, WHR, IFF, MU, EXC, STE, PVH, GPS, AMGN, AMAT, AMGN, HAL, LEN, SCHW, LEG, CERN, MCO, LRCX, MOS, AVB, MOS, BMY, WMT, CTAS, CMI, COO, DGX, ADM, NEE, CTRA, MOS, PGR, NVDA, HUM, CMI, NVDA, SLB, FMC, TECH, FCX, AME, HES, SCHW, AAPL, DGX, TGT, CMI, HES, SWKS, FRT, TJX, KSU, LRCX, BLK, SHW, CAT, CI, UNP, SNA, ESS, STZ, L, TROW, FRT, HUM, ATVI, TROW, EXPD, VTR, FRT, TFX, LUMN, NVDA, A, CMA, EXC, MOS, NVDA, NUE, BBWI, ROST, LMT, NEM, APH, IVZ, CMI, FCX, RL, WY, MS, ATVI, AMGN, HUM, FCX, DHI, NWL, CMI, TAP, SWKS, EL, AES, IVZ, ALB, MLM, INTU, HAS, LEN, OXY, KSU, MGM, BEN, MOS, TJX, JBHT, NTAP, STT, NVDA, UNP, PVH, PHM, NTAP, PCAR, NEE, MOS, JNPR, MLM, NEM, XRAY, SBAC, BWA, HES, RL, ODFL, PSA, LUV, MOS, LEG, AMAT, SBUX, JCI, OXY, BBWI, TGT, HUM, SPG, ALB, ABC, DHI, IVZ, TECH, ORCL, LEN, PVH, PHM, JNPR, COO, LEN, IVZ, MAR, HES, VFC, MOS, IVZ, PEAK, CMI, PPG, TFX, VTR, NEM, J, NEE, LLY, CAH, OKE, ROST, MOS, LUV, PVH, MMC, XLNX, LOW, PHM, NTAP, ALB, LEN, SBAC, RHI, PHM, NVDA, CMI, IEX, CI, MCO, SBAC, LEN, EQR, ROK, CTRA, HRL, MOS, DHI, POOL, SBAC, MOS, LEN, JNPR, EOG, EA, NTAP, IVZ, TSN, IEX, CMA, PEAK, RJF, HUM, PHM, PEAK, JPM, MS, HUM, MS, BLK, PVH, MOS, PAYX, KIM, MU, LEN, MU, POOL, MRK, COF, CTXS, MU, LOW, POOL, HES, SCHW, KLAC, LEG, SLB, HES, FITB, RJF, TROW, MOS, COF, PVH, CMI, PHM, MRO, PXD, DHI, STE, EOG, ATVI, HWM, STZ, PHM, NVDA, LHX, VMC, HUM, CI, QCOM, HSY, MOS, BBY, CTSH, VLO, MCO, SWKS, CTRA, BAX, APA, GPS, MOS, TER, MS, ZION, ATVI, MS, CCL, KEY, HAL, LUMN, KR, MAS, MOS,

STZ, RMD, DTE, ECL, MGM, LEN, PNW, HWM, FITB, PHM, USB, TFC, ZION, KEY, MOS, RCL, DRE, SNA, POOL, RE, BAC, HES, WEC, CTSH, CI, MGM, MLM, ABC, DHI, MGM, ATVI, MOS, PHM, UHS, ES, EOG, VLO, LEN, MGM, POOL, PVH, LEN, PHM, MGM, YUM, PVH, APA, CMA, WHR, AVY, MU, AIG, ZION, JBHT, AIG, NEM, EQR, RJF, NEM, VMC, LEN, TSN, JPM, RHI, STT, FITB, MU, FMC, AIG, AES, STT, GLW, LNC, UNH, KEY, WFC, PPG, SBAC, AES, KEY, TRV, MRO, PNC, ODFL, IPG, MGM, RSG, AIG, HIG, J, MGM, DHI, AES, HRL, GS, AES, ALL, HIG, PLD, STZ, GLW, IPG, KIM, LNC, LEN, LNC, CTAS, PLD, MS, HIG, DRE, NVDA, PEAK, UNH, DRE, NEM, PLD, KSU, MU, RCL, TECH, ARE, MU, PCAR, HES, IEX, IRM, MGM, MU, LNC, ATVI, DHI, GPS, COO, LNC, VFC, DRE, MU, PH, PNC, STT, HIG, VLO, ZION, FITB, ODFL, BA, ROK, DHI, APH, CI, FITB, GE, EXPD, IPG, BWA, DHI, IVZ, COF, MO, KEY, LEN, COF, FITB, USB, FITB, LEN, SWKS, GLW, SWKS, UNH, USB, DRE, MU, COF, AIG, IP, AIG, HWM, NEM, LNC, IP, PLD, NWL, LNC, HUM, HIG, TXT, BWA, MGM, TXT, UNH, ROK, DRE, FITB, ZBH, AXP, DRE, MGM, ORCL, STT, SNA, IVZ, MAS, UHS, TECH, CERN, NWL, PKI, FITB, MGM, ZION, CI, ZION, MGM, MOS, TFC, HIG, ODFL, ZION, MU, UHS, CTXS, BLK, UDR, TER, CI, CSX, PCAR, HWM, SBUX, KEY, IPG, LEN, BLK, IP, KEY, VMC, RL, CI, TJX, HUM, CI, CNP, IP, LEN, JKHY, LNC, MAA, RCL, MTB, KIM, CI, ROST, MU, AIG, IPG, AXP, WHR, AIG, VNO, IPG, KEY, HIG, COF, ZION, TXT, C, FMC, ODFL, KEY, ARE, AIG, BBWI, MAR, WMB, NSC, HIG, FITB, TFC, HUM, DE, AIG, EXPD, RMD, KIM, AIG, KSU, FITB, MOS, AIG, BBY, CMCSA, SPG, AIG, MGM, MSI, AIG, ZION, CMA, VLO, LEG, AIG, RJF, EA, CTAS, MGM, HIG, MCO, YUM, HSY, AIG, BBWI, SCHW, MU, LEN, MU, BWA, LEN, KIM, VLO, LHX, MSI, FITB, DE, LEN, TROW, ROP, UNH, AIG, IVZ, NEM, COF, AIG, PHM, AIG, SWKS, PLD, TXT, ODFL, CTAS, MAS, EL, MOS, DRE, PGR, MOS, CI, COO, BBWI, MGM, EBAY, SWKS, MOS, KLAC, RHI, CMCSA, ODFL, CMA, UNH, LUV, MGM, WHR, NVDA, ODFL, DRI, IVZ, AIG, MGM, FAST, AIG, ODFL, A, WFC, KEY, HIG, ZION, CMI, TFX, MGM, FITB, TXT, VLO, C, ZION, RJF, MGM, HAL, UHS, IVZ, CTXS, PBCT, NVDA, STE, AJG, COO, NEM, LEN, AIG, MGM, MSI, IP, GILD, TXT, JNPR, AIG, TMO, KEY, SRE, IPG, FITB, PCAR, AIG, PVH, AIG, C, PCAR, LNC, MGM, MLM, FDX, ROST, MGM, RCL, KEY, AIG, MGM, AES, MCK, PXD, MGM, ZION, AIG, MGM, J, AIG, VNO, ZION, VMC, MLM, C, ZION, CHRW, TXT, PHM, KSU, UHS, HUM, LHX, RMD, MLM, UDR, GILD, STE, AON, MAS, AIG, TXT, EA, UHS, SEE, BAX, RL, ROST, PXD, BAX, CMCSA, TXT, NTAP, CAH, HSY, HAL, SBAC, PBCT, NEM, ZION, ODFL, LEN, NTAP, AIG, TER, MCO, MTB, KR, NEE, SJM, MCO, MO, ODFL, TXT, BBWI, JNPR, JCI, AES, MCO, AES, MSI, IVZ, PGR, GS, MOS, WY, STT, ABT, FITB, TFX, AIG, ECL, CVS, CTXS, SWK, MOS, UHS, MOS, IFF, PKI, AES, CL, BWA, EL, AEE, CSCO, SWK, TGT, NTAP, INTU, MOS, LEN, LNC, DRI, LNC, ABC, HIG, APH, PVH, GS, NTAP, NLOK, RCL, TER, MU, BLK, MCK, MOS, ORCL, WHR, NVDA, HWM, KLAC, MU, LUV, NEM, CTRA, TSN, BBWI, EA, JNPR, STZ, CI, MGM, MCO, FITB, WMB, CHRW, GPC, WFC, ZION, WFC, FITB, NVDA, TSCO, AME, ODFL, FMC, HAL, MCK, VMC, MGM, DHI, BK, HAL, EOG, HAL, JNPR, CAT, TSN, RCL, HAL, CTRA, HUM, MU, SWKS, HRL, WBA, LOW, MAS, LEN, MGM, TER, JBHT, LNC, AIG, MSI, CTRA, AIG, STZ, MSI, CERN, DRE, RCL, XLNX, TER, AIG, KEY, MOS, GPS, NVDA, LNC, MSI, AIG, NVDA, IPG, NVDA, HES, NVDA, UHS, TER, NOC, LMT, LOW, WY, SEE, SHW, HAL, NWL, HES, AME, SEE, NVDA, IPG, A, HAS, LEN, RL, JNPR, CLX, MRO, IVZ, TER, NVDA, INTU, PEG, CTRA, PKI, VLO, EIX, AES, XLNX, VLO, A, EXC, LUV, NLOK, SBUX, WY, CTRA, F

This sequence of observations constitutes the following sparse frequency matrix in Fig. 3.

8.3.4 Detected groups

Here are the detected groups in model $\hat{\mathbb{P}}$:

$\mathcal{V}_1 =$ CB (Financials), GS (Financials), IBM (Information Technology), AMGN (Health Care), MMM (Industrials), CVX (Energy), FDX (Industrials), COST (Consumer Staples), UNP (Industrials), AVB (Real Estate), SPG (Real Estate), HD (Consumer Discretionary), JNJ (Health Care), KMB (Consumer Staples), JPM (Financials), GD (Industrials), ESS (Real Estate), CSCO (Information Technology), INTC (Information Technology), PSA (Real Estate), MTB (Financials), HON (Industrials), BXP (Real Estate), GWW (Industrials), NOC (Industrials), BA (Industrials), APD (Materials), TRV (Financials), RTX (Industrials), PEP (Consumer Staples), CAT (Industrials), AMAT (Information Technology), BDX (Health Care), PPG (Materials), SHW (Materials), UPS (Industrials), PH (Industrials), PFE (Health Care), NSC (Industrials), ECL (Materials), DE (Industrials), ADP (Information Technology), GE (Industrials), SRE (Utilities), WMT (Consumer Staples), PG (Consumer Staples), DIS (Communication Services), NEE (Utilities), T (Communication Services), ITW (Industrials), XOM (Energy), PNC (Financials), BAC (Financials), AXP (Financials), VZ (Communication Services), SYK (Health Care), CLX (Consumer Staples), BEN (Financials), C (Financials), LLY (Health Care), SNA (Industrials), FRT (Real Estate), KO (Consumer Staples), APA (Energy), WM (Industrials), DHR (Health Care), IFF (Materials), CVS (Health Care), ALL (Financials), TT (Industrials), MDT (Health Care), BMY (Health Care), NKE (Consumer Discretionary), COP (Energy), EFX (Industrials), DTE (Utilities), DUK (Utilities), OXY (Energy), ETR (Utilities), KR (Consumer Staples), YUM (Consumer Discretionary), CL (Consumer Staples), DD (Materials), DGX (Health Care), WBA (Consumer Staples), SO (Utilities), BK (Financials), NTRS (Financials), CAG (Consumer Staples), SYY (Consumer Staples), DOV (Industrials), EIX (Utilities), GIS (Consumer Staples), TFC (Financials), ETN (Industrials), BAX (Health Care), EMR (Industrials), EXC (Utilities), AEP (Utilities), AFL (Financials), ARE (Real Estate), AVY (Materials), OMC (Communication Services), FE (Utilities), D (Utilities), VNO (Real Estate), EQR (Real Estate), NI (Utilities), CPB (Consumer Staples), PEG (Utilities), K (Consumer Staples), LUMN (Communication Services), PPL (Utilities), HAS (Consumer Discretionary), MKC (Consumer Staples), ED (Utilities), EMN (Materials), RSG (Industrials), AEE (Utilities), ES (Utilities), CINF (Financials), UDR (Real Estate), CCL (Consumer Discretionary), XRAY (Health Care), WEC (Utilities), GPC (Consumer Discretionary), L (Financials), OKE (Energy), MAA (Real Estate), O (Real Estate), BLL (Materials), AJG (Financials), PNR (Industrials), GL (Financials), PNW (Utilities)

$\mathcal{V}_2 =$ IP (Materials), ZBH (Health Care), CMI (Industrials), BLK (Financials), LMT (Industrials), MCK (Health Care), CI (Health Care), UNH (Health Care), PXD (Energy), MCD (Consumer Discretionary), TMO (Health Care), INTU (Information Technology), TXN (Information Technology), ORCL (Information Technology), WHR (Consumer Discretionary), QCOM (Information Technology), ROK (Industrials), EOG (Energy), MLM (Materials), RE (Financials), KLAC (Information Technology), RL (Consumer Discretionary), AON (Financials), EA (Communication Services), LOW (Consumer Discretionary), CMCSA (Communication Services), EBAY (Consumer Discretionary), STZ (Consumer Staples), WFC (Financials), HPQ (Information Technology), ROP (Industrials), ABT (Health Care), SWK (Industrials), MS (Financials), CTXS (Information Technology), KSU (Industrials), MCO (Financials), MRK (Health Care), EL (Consumer Staples), SJM (Consumer Staples), ADI (Information Technology), JCI (Industrials), VMC (Materials), MSI (Information Technology), SBUX (Consumer Discretionary), GILD (Health Care), CTAS (Industrials), UHS (Health

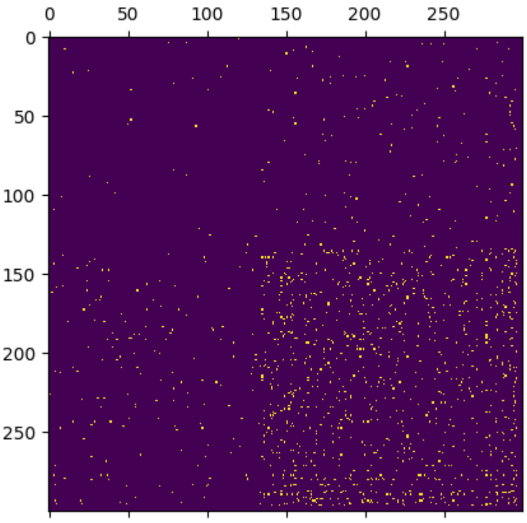

Fig. 3 A plot of the matrix $\{\mathbb{1}[\hat{F}_{ij} > 0]\}_{i,j}$, where the rows and columns are sorted according to the improved clustering. We plotted the matrix like such because $\hat{F}$ is quite sparse due to the trajectory's length $\ell = 2451$ being quite short: the minimum, median, mean, and maximum of the entries of the matrix $\{\hat{F}_{i,j}\}_{i,j}$ are 0, 0, $\ell/n^2 \approx 0.027$, and 14, respectively.

Care), SLB (Energy), MMC (Financials), MCHP (Information Technology), NLOK (Information Technology), HSY (Consumer Staples), TGT (Consumer Discretionary), TROW (Financials), USB (Financials), XLNX (Information Technology), MO (Consumer Staples), DRI (Consumer Discretionary), ROST (Consumer Discretionary), BBY (Consumer Discretionary), TFX (Health Care), LHX (Industrials), PAYX (Information Technology), GPS (Consumer Discretionary), COF (Financials), MRO (Energy), SCHW (Financials), MAR (Consumer Discretionary), JBHT (Industrials), PGR (Financials), TJX (Consumer Discretionary), ADM (Consumer Staples), HWM (Industrials), A (Health Care), STT (Financials), HES (Energy), ABC (Health Care), CSX (Industrials), NUE (Materials), VFC (Consumer Discretionary), JKHY (Information Technology), CAH (Health Care), XEL (Utilities), TECH (Health Care), DVN (Energy), HIG (Financials), NWL (Consumer Discretionary), WY (Real Estate), PCAR (Industrials), MAS (Industrials), POOL (Consumer Discretionary), BBWI (Consumer Discretionary), DHI (Consumer Discretionary), PLD (Real Estate), LNC (Financials), ZION (Financials), APH (Information Technology), CNP (Utilities), CMA (Financials), CTSH (Information Technology), FAST (Industrials), IEX (Industrials), EXPD (Industrials), TSCO (Consumer Discretionary), TXT (Industrials), CHRW (Industrials), CERN (Health Care), PBCT (Financials), RCL (Consumer Discretionary), CTRA (Energy), PEAK (Real Estate), TAP (Consumer Staples), SEE (Materials), KIM (Real Estate), ALB (Materials), KEY (Financials), RMD (Health Care), STE (Health Care), DRE (Real Estate), BWA (Consumer Discretionary), RHI (Industrials), FMC (Materials), J (Industrials), LEG (Consumer Discretionary), CMS (Utilities), VTR (Real Estate), IRM (Real Estate), PKI (Health Care), HRL (Consumer Staples), AME (Industrials), IVZ (Financials), RJF (Financials), LUV (Industrials)

$\mathcal{V}_3 =$ AAPL (Information Technology), NVDA (Information Technology), LRCX (Information Technology), HUM (Health Care), AMT (Real Estate), HAL (Energy), FCX (Materials), COO (Health Care), JNPR (Information Technology), CCI (Real Estate), NTAP (Information Technology), VLO (Energy), FITB (Financials), SBAC (Real Estate), NEM (Materials), GLW (Information Technology), AIG (Financials), WMB (Energy), ATVI (Communication Services), LEN (Consumer Discretionary), TER (Information Technology), MU (Information Technology), MGM (Consumer Discretionary), PVH (Consumer Discretionary), TSN (Consumer Staples), MOS (Materials), PHM (Consumer Discretionary), ODFL (Industrials), SWKS (Information Technology), AES (Utilities), IPG (Communication Services)

References

[1] Van Werde, A., Senen–Cerda, A., Kosmella, G., Sanders, J.: Detection and evaluation of clusters within sequential data (2023)

[2] Sanders, J., Proutière, A., Yun, S.-Y.: Clustering in Block Markov Chains. The

Annals of Statistics (2020)

[3] Sanders, J., Proutière, A., Yun, S.-Y.: Clustering in Block Markov Chains. The Annals of Statistics (2020)

[4] Kullback, S., Leibler, R.A.: On information and sufficiency. The Annals of Mathematical Statistics (1951)

[5] Akaike, H.: A new look at the statistical model identification. IEEE Transactions on Automatic Control (1974)

[6] Bozdogan, H.: Model selection and Akaike's Information Criterion (AIC): The general theory and its analytical extensions. Psychometrika (1987)

[7] Anderson, D., Burnham, K.: Model selection and multi-model inference. Second. NY: Springer-Verlag (2004)

[8] Ding, J., Tarokh, V., Yang, Y.: Model selection techniques: An overview. IEEE Signal Processing Magazine (2018)

[9] Sanders, J., Van Werde, A.: Singular value distribution of dense random matrices with block Markovian dependence. Stochastic Processes and their Applications (2023)

[10] Sanders, J., Senen–Cerda, A.: Spectral norm bounds for Block Markov Chain random matrices. Stochastic Processes and their Applications (2023)

[11] Paulin, D.: Concentration inequalities for Markov chains by Marton couplings and spectral methods. Electronic Journal of Probability (2015)

[12] Bai, Z., Silverstein, J.W.: Spectral Analysis of Large Dimensional Random Matrices, (2010)

[13] Sanders, J., Van Werde, A.: Singular value distribution of dense random matrices with block Markovian dependence. Stochastic Processes and their Applications (2023)

[14] Zhang, X., Zhao, J., LeCun, Y.: Character-level Convolutional Networks for Text Classification. Advances in Neural Information Processing Systems (2015)

[15] Lehmann, J., Isele, R., Jakob, M., Jentzsch, A., Kontokostas, D., Mendes, P.N., Hellmann, S., Morsey, M., Van Kleef, P., Auer, S., Bizer, C.: DBpedia – A Large-scale, Multilingual Knowledge Base Extracted from Wikipedia. Semantic web (2015)

[16] Russel, M.: 10.000 Books and Their Genres standardized. Accessed at https://www.kaggle.com/code/michaelrussell4/gutenberg-book-genre-feature-engineering/data (2021)

[17] Bamman, D., Smith, N.A.: New Alignment Methods for Discriminative Book Summarization. arXiv preprint arXiv:1305.1319 (2013). Accessed at https://www.kaggle.com/datasets/ymaricar/cmu-book-summary-dataset

[18] Lang, K.: NewsWeeder: Learning to Filter Netnews. In: Proceedings of the Twelfth International Conference on Machine Learning (1995)

[19] Gómez Hidalgo, J.M., Bringas, G.C., Sánz, E.P., García, F.C.: Content based SMS spam filtering. In: Proceedings of the 2006 ACM Symposium on Document Engineering (2006)

[20] Cormack, G.V., Hidalgo, J.M.G., Sánz, E.P.: Feature Engineering for Mobile (SMS) Spam Filtering. In: Proceedings of the 30th Annual International ACM SIGIR Conference on Research and Development in Information Retrieval (2007)

[21] Cormack, G.V., Gómez Hidalgo, J.M., Sánz, E.P.: Spam Filtering for Short Messages. In: Proceedings of the Sixteenth ACM Conference on Conference on Information and Knowledge Management (2007)

[22] Almeida, T.A., Hidalgo, J.M.G.: SMS Spam Collection Data Set. https://www.kaggle.com/datasets/uciml/sms-spam-collection-dataset

[23] Lewis, D.D., Yang, Y., Russell-Rose, T., Li, F.: RCV1: A New Benchmark Collection for Text Categorization Research. Journal of machine learning research (2004)
